# Molecular Bidents with Two Electrophilic Warheads as a New Pharmacological Modality

**DOI:** 10.1021/acscentsci.3c01245

**Published:** 2024-02-26

**Authors:** Zhengnian Li, Jie Jiang, Scott B. Ficarro, Tyler S. Beyett, Ciric To, Isidoro Tavares, Yingde Zhu, Jiaqi Li, Michael J. Eck, Pasi A. Jänne, Jarrod A. Marto, Tinghu Zhang, Jianwei Che, Nathanael S. Gray

**Affiliations:** † Department of Chemical and Systems Biology, Stanford Cancer Institute, ChEM-H, Stanford University, Stanford, California 94305, United States; ‡ Lowe Center for Thoracic Oncology, Dana-Farber Cancer Institute, Boston, Massachusetts 02215, United States; § Department of Cancer Biology, Dana-Farber Cancer Institute, Boston, Massachusetts 02215, United States; ∥ Department of Medical Oncology, Dana-Farber Cancer Institute, Boston, Massachusetts 02215, United States; ⊥ Blais Proteomics Center, Center for Emergent Drug Targets, Dana-Farber Cancer Institute, Boston, Massachusetts 02215, United States; # Department of Medicine, Harvard Medical School, Boston, Massachusetts 02215, United States; ¶ Department of Biological Chemistry and Molecular Pharmacology, Harvard Medical School, Boston, Massachusetts 02215, United States; ■ Department of Pathology, Brigham and Women’s Hospital and Harvard Medical School, Boston, Massachusetts 02215, United States

## Abstract

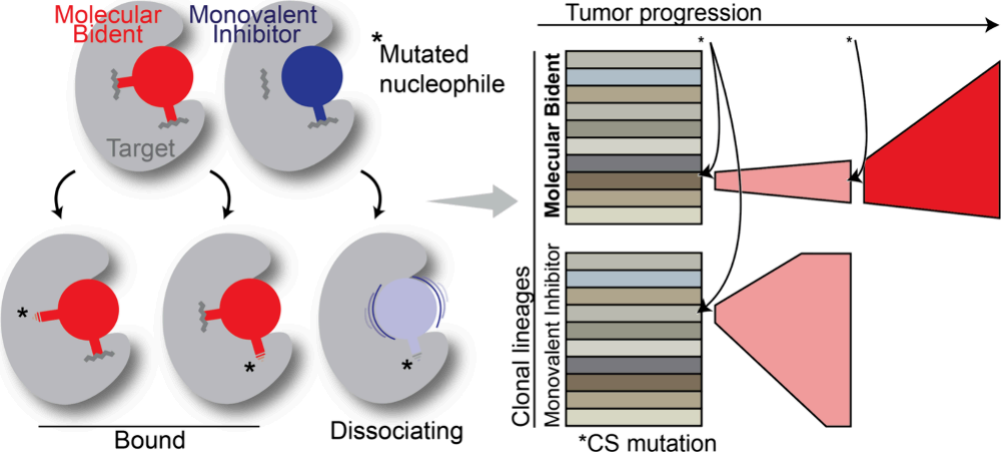

A systematic strategy to develop dual-warhead inhibitors is introduced to circumvent the limitations of conventional covalent inhibitors such as vulnerability to mutations of the corresponding nucleophilic residue. Currently, all FDA-approved covalent small molecules feature one electrophile, leaving open a facile route to acquired resistance. We conducted a systematic analysis of human proteins in the protein data bank to reveal ∼400 unique targets amendable to dual covalent inhibitors, which we term “molecular bidents”. We demonstrated this strategy by targeting two kinases: MKK7 and EGFR. The designed compounds, ZNL-8162 and ZNL-0056, are ATP-competitive inhibitors that form two covalent bonds with cysteines and retain potency against single cysteine mutants. Therefore, molecular bidents represent a new pharmacological modality with the potential for improved selectivity, potency, and drug resistance profile.

## Abstract 2

A systematic approach to target proteins through two simultaneous covalent bonds addresses resistance to traditional covalent inhibitors upon mutation of a single anchoring nucleophilic residue.

## Introduction

Despite decades of small molecule drug discovery efforts, only a small fraction of the human proteome has been successfully drugged.^1,2^ The recent resurgence of covalent drug discovery holds the potential to expand the druggable proteome, as illustrated by a growing list of approved drugs for diverse targets including EGFR, BTK, SARS-Cov2 M_pro_, as well as for KRAS^G12C^, formerly considered to be “undruggable”.^3,4^ Typical covalent inhibitors feature a single warhead that forms a covalent bond with a side chain of a specific nucleophilic residue, most commonly cysteine.^5^ This covalent mode of action results in inhibitors that exhibit significant improvements in potency and efficacy compared to traditional noncovalent drugs due to sustained target engagement. Importantly, they have the ability to bind and inhibit targets that lack well-defined noncovalent ligand binding sites, thus opening broader opportunities for targeting proteins classified as “undruggable”.^6^ These advantages have led to a surge in the exploration of covalent modulators, with almost 9000 binders reported across many protein families, including transcription factors.^7^ Despite the success, covalent inhibitors have several limitations, one of which is acquired resistance due to the mutation of the nucleophilic residue involved in covalent bond formation. For example, mutation of Cys797 of EGFR is a major resistance mechanism to osimertinib, a covalent inhibitor that is currently prescribed as first-line therapy for mutant EGFR-dependent non-small cell lung cancer (NSCLC).^8,9^ Likewise, Cys481 mutation is a common resistance mechanism to the covalent BTK inhibitor ibrutinib in chronic lymphocytic leukemia (CLL).^9^ A similar mechanism of resistance has also been documented for the recently approved covalent inhibitors of KRAS^G12C^.^10,11^ Therefore, there is an urgent need to develop new chemical approaches that can circumvent single cysteine mutation-based resistance mechanisms.

**Figure 1:**
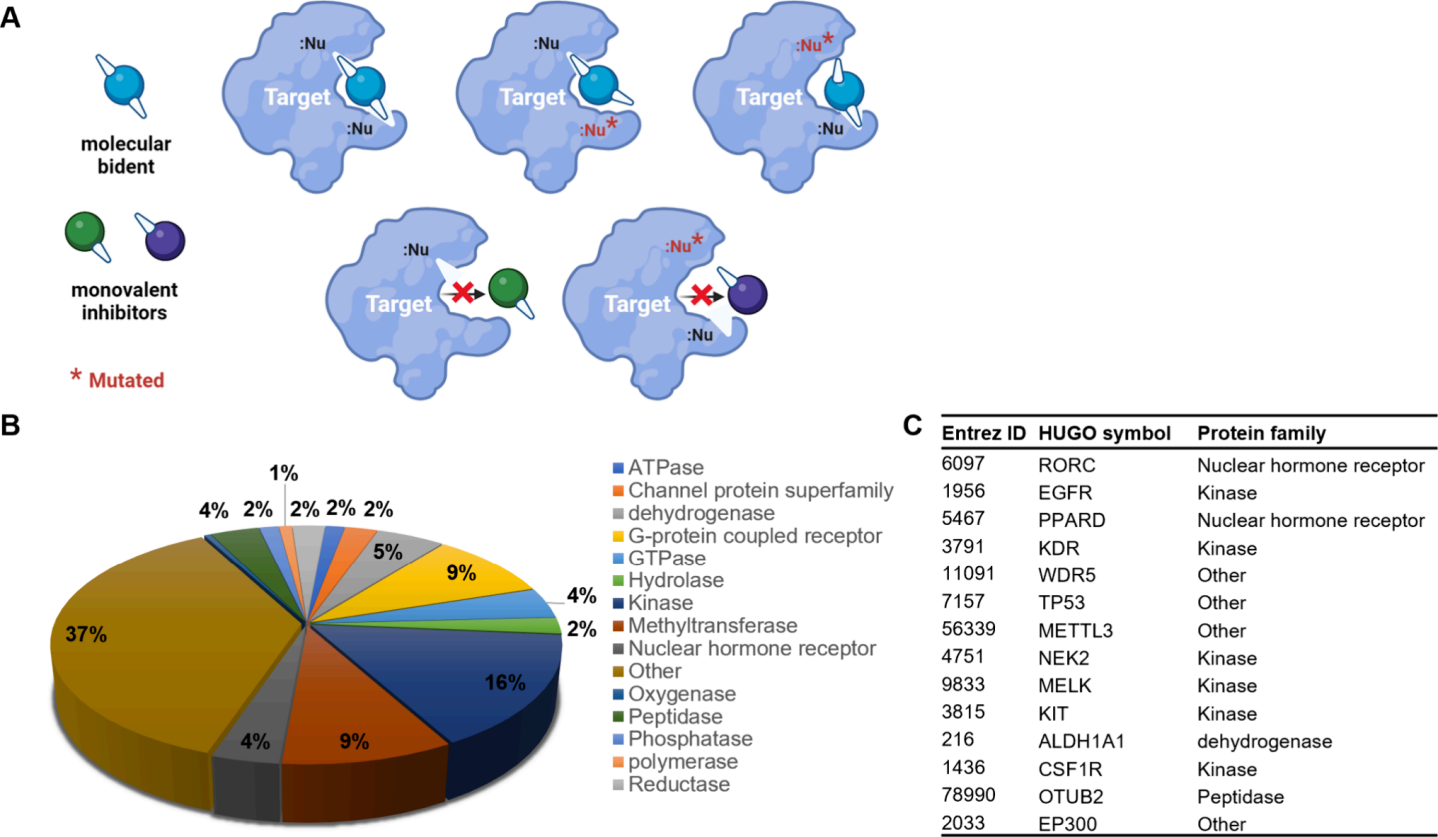
**Molecular bident concept**. (**A**) Schematic showing molecular bidents and monovalent covalent inhibitors (left) and their interactions with target proteins (right). (**B**) Distribution of protein classes from PDB database in which multiple cysteines are in close proximity to bound small molecule ligands. (**C**) Representative targets for molecular bidents according to structural informatics (see Methods).

Here, we report a general strategy to delay or prevent acquired resistance by developing dual covalent inhibitors that simultaneously react with two distinct cysteine residues. We call this type of covalent inhibitor a “molecular bident” (Figure 1A). Dual covalent targeting has recently been described as critical step in signaling cascade triggered by a plant hormone, strigolactone,^12^ illustrating a unique biological effect of the strategy. A recent dual-warhead FGFR4 inhibitor was reported,^13^ where Chen et.al. reported dual covalency in a cocrystal structure and indirectly inferred from *in vitro* assays. However, rationally designed small molecules to form designated double covalent bonds have not been proposed systemically across the proteome as a pharmacological modality.

We used computational screening, synthetic chemistry, and protein mass spectrometry to enable and validate this strategy. A systematic analysis of publicly accessible ligand-bound protein structures identified pairs of cysteines that are within an appropriate distance from a ligand to enable dual covalent targeting. The analysis revealed approximately 400 such proteins with existing ligands which could be leveraged to rapidly transform into molecular bidents. To implement the strategy, we first tried mitogen-activated protein kinase kinase 7 (MKK7), which phosphorylates JNK to initiate downstream signaling in response to inflammatory cytokines. We developed ZNL-8162, an ATP-competitive kinase inhibitor with two chemically reactive groups simultaneously targeting Cys218 and Cys276 of MKK7.

To determine whether this strategy can be generalized and to identify potential prototype therapeutics, we developed a second molecular bident for mutant EGFR, a well-established driver of cancer signaling. Guided by structural analysis, we designed ZNL-0056, an ATP-competitive inhibitor that targets both the Cys797 and Cys775 in the ATP binding site of EGFR. ZNL-0056 is the first compound capable of targeting a nonconserved and partially buried Cys775 (behind the so-called gatekeeper residue) located at the back of the ATP-binding pocket in EGFR. Taken together, the design and synthesis of these small molecule inhibitors illustrates a novel “molecular bidents” strategy for inhibiting kinases. The corresponding structure-based database presents a wealth of novel targeting opportunities and is provided for the community for further exploration of targets.

## Results and Discussion

### Systematic Analysis of Structural Information Identifies Targets for Molecular Bident Development

To identify candidates for molecular bident design, we started with analysis of available protein ligand complex structures with two or more cysteines in the binding pocket. From structures of human protein ligand complexes in the Protein Data Bank (PDB) database, we identified 2136 protein ligand complexes that contain multiple cysteines within close proximity (7 Å) to their bound ligands (data file S1). This represents 31% of the total human proteins in the PDB. These structures represent 404 unique proteins, including kinases, GTPases, nuclear hormone receptors, and hydrolases (Figure 1B). To gain more structural insights, we use protein kinases to illustrate the co-occurring cysteines in ATP binding sites due to their well-characterized folding structures and functions of key regions. The characteristic kinase structure allows inference of the residue locations and conservation even for the ones with no crystal structures. Chaikuad et al.^14^ and Leproult et al.^15^ showed that many protein kinases (over 200) have cysteines in and around the ATP binding site with strong preference at certain “hotspots” such as hinge and DFG regions, in which more than 40 have 2 or more potentially ligandable cysteines. The pairs also mostly distribute in the way that at least one member is at those “hotspots”. This indicates the occurrence of cysteine pairs in kinase domains is likely the reflection of cysteine abundance at certain locations. Structural elements such as disulfides and zinc fingers are not considered here. Among all of the structures our analysis identified, only 226 (representing 34 unique proteins) contain a covalent cysteine-bound small molecule; therefore, ∼90% of the identified potential targets have either no structurally characterized or reported covalent binders highlighting the unique targeting opportunities uncovered by our analysis. Some examples include ALDH1A2 (Cys319 and Cys320, PDB: 6ALJ), RORγt (Cys285 and Cys320, PDB: 6BR2), KDR (Cys917 and Cys1043, PDB: 1Y6A), and mutant P53 (Cys220 and Cys229, PDB: 3ZME). All these proteins have been implicated as disease-relevant targets.^16−21^ In addition to these, many known drug targets are identified in our list of hits, including EGFR, KIT, and peroxisome proliferator-activated receptor (PPAR; Figure 1C). The complete list can be found in the associated data files (data file S2). Taken together, our results illustrate that a large number of proteins with defined pockets may be amenable to the development of site specific molecular bidents. It should be noted that the existence of a cysteine does not guarantee its ligandability. For example, many cysteines are known to be involved in post-translational-modification and redox sensing and may not be accessible to a new covalent inhibitor. For example, cysteines involved in stable disulfide bonds or involved metal coordination may not be reactive toward small molecule electrophiles. In addition, the intrinsic reactivity of the cysteine thiol is sensitive to its p*K*_a_ which is controlled by the local microenvironment. These additional factors will contribute toward the feasibility of generating molecular bidents targeting a particular pair of cysteines.

### Dual-Covalent Inhibitor ZNL-8162 Exhibits Covalency-Driven Inhibition of MKK7 Kinase Activity and Downstream Signaling in Cells

**Figure 2:**
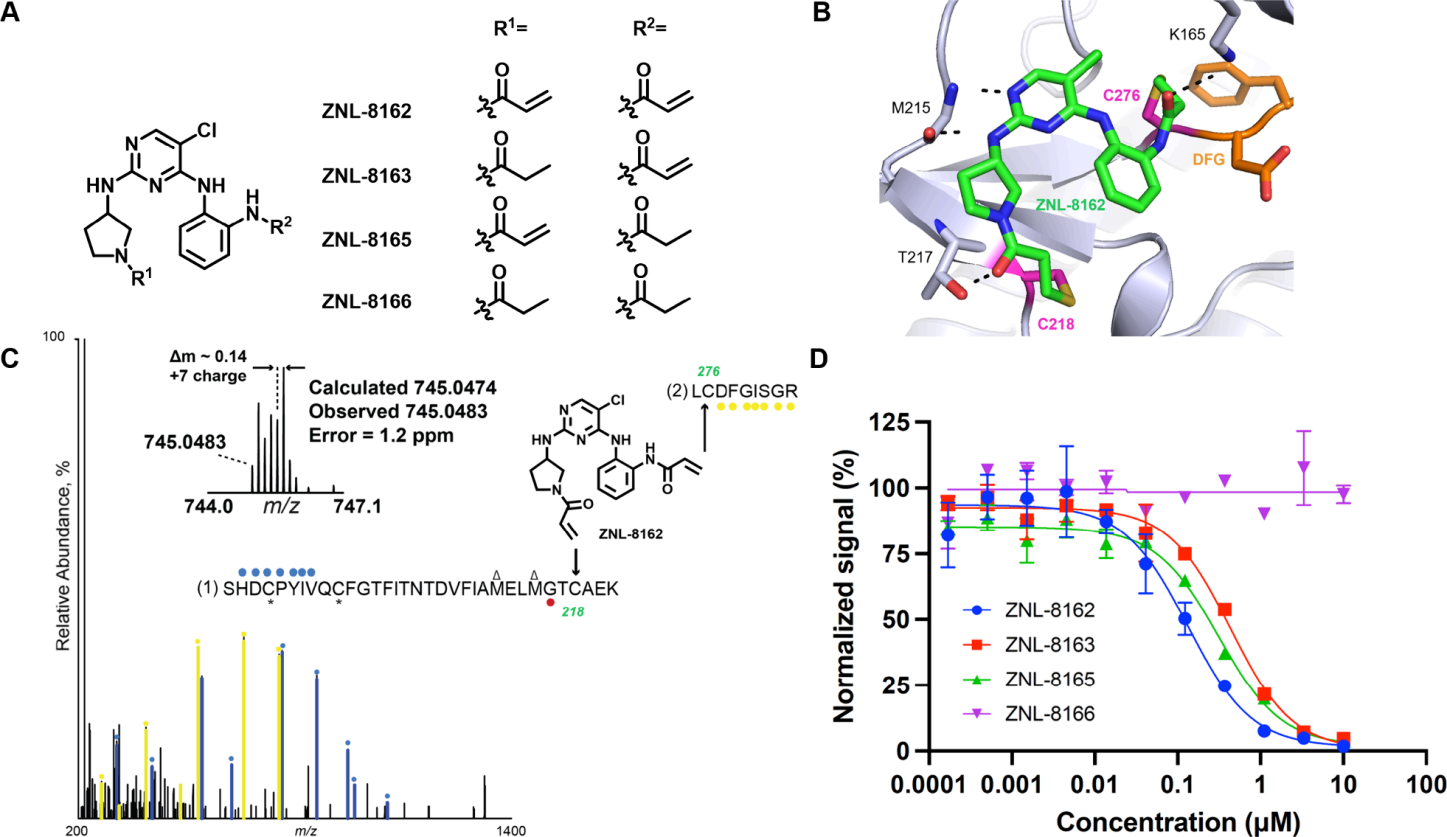
**ZNL-8162 is a molecular bident for MKK7**. (**A**) Compound structures for MKK7-targeting molecular bident (ZNL-8162) and its relatives lacking one or both cysteine-reactive groups. (**B**) Covalent modeling for ZNL-8162 engagement of MKK7(PDB: 6YG3). (**C**) MS/MS spectrum of ZNL-8162 covalently modifying Cys218 and Cys276 of MKK7. Red and yellow circles indicate ions of type y for sequences marked (1) and (2), respectively. Blue circles indicate ions of b for sequence (1). *, carbamidomethyl cysteine, Δ, oxidized methionine. (**D**) MKK7 binding data were obtained from KdELECT assays (DiscoverX). Data are presented as mean ± s.d. of *n* = 2 biologically independent samples.

To demonstrate the molecular bident strategy, we chose to target MKK7 as a proof-of-concept example identified through the structural analysis. MKK7 is a member of the mitogen-activated kinase kinase (MAP2K) subfamily, and it is an activator of c-Jun N-terminal kinase (JNK) signaling.^22,23^ In the ATP binding site, MKK7 has three cysteines, Cys276 at DFG-1 that is immediately before the activation DFG motif, Cys147 at the flexible glycine rich loop connecting β_1_ and β_2_ strands, and Cys218 at the lower hinge.^24,25^ First, we analyzed published covalent MKK7 inhibitors and their binding modes. The 2-aminophenyl acrylamide of SM1-71^26^ and BSJ-04-122^27^ has been previously reported to covalently label Cys276 (DFG-1 cysteine), while the pyrrolidine acrylamide of Type II covalent inhibitor TL-10-105^28^ labels Cys218 of MKK7. The binding modes of these compounds suggested that the aminopyrimidine group, one of most common moieties for kinase hinge binding, could be used to design a molecular bident targeting Cys218 and Cys276 simultaneously while keeping key hinge hydrogen bonds intact. We examined the distance between Cys218 and Cys276 and the orientation of the sulfhydryl groups with respect to the bound ligands to design ZNL-8162 (Figure 2A). Docking of ZNL-8162 into the MKK7 crystal structure (PDB: 6YG3) suggested a nearly identical binding mode for the aminopyrimidine core, with covalent linkages predicted with Cys218 and Cys276, as designed (Figure 2B). ZNL-8162 was synthesized along with a corresponding single warhead and fully reversible counterparts (ZNL-8163, ZNL-8165, and ZNL-8166, respectively). These control compounds served as tools to deconvolute the contributions of the dual electrophiles.

To determine whether ZNL-8162 can form two covalent bonds simultaneously with Cys218 and Cys276 of MKK7, we used mass spectrometry to identify the existence of the cross-linked peptides. After compound treatment and trypsin digestion,^29^ a new species corresponding to a two-peptide cross-link at Cys218 and Cys276 was identified with a mass shift consistent with the expected molecular weight of ZNL-8162 (Figure 2C), indicating that the compound bridges two cysteines with a single MKK7 molecule.

Next, we characterized the inhibitory effects of the dual-covalent compound and its relatives on enzymatic activity. ZNL-8162 was the most potent MKK7 inhibitor (IC_50_ of 133 nM; Figure 2D). Both monocovalent inhibitors ZNL-8163 (IC_50_ = 437 nM) and ZNL-8165 (IC_50_ = 313 nM) showed an approximate ∼3-fold loss of potency. The reversible control ZNL-8166 was completely inactive against MKK7 (IC_50_ > 10 000 nM), indicating a strong dependency on covalent binding. We next evaluated their inhibition on the downstream p-JNK1/2, a downstream target of MKK7, in cell. ZNL-0162 exhibited strong inhibition of p-JNK1/2, while the monocovalent inhibitors ZNL-8163 and ZNL-8165 exhibited less potency. In contrast, the reversible compound ZNL-8166 did not exhibit any inhibition on p-JNK1/2 up to a concentration of 10 μM, consistent with biochemical activity. The cellular signaling inhibition demonstrated that the bident ZNL-8162 had superior cellular activity over its monocovalent counterpart (Figure S1).

These data indicate that molecular bident compounds such as these would remain active against targets that have undergone mutations at either one of the targeted nucleophilic residues. Therefore, the molecular bident drugs are expected to remain effective for longer time periods when compared to current covalent drugs, as simultaneous mutation of both nucleophilic residues is less likely to emerge. Our data using ZNL-8163 and ZNL-8165 indicate that the loss of one covalent bond does not result in inactive compounds. We used the ZNL-8163 and ZNL-8165 IC_50_ data to estimate the potency of ZNL-8162; thus, assuming independent contributions from both covalent bonds, we derived IC_50_ ≈ 182 nM (supp. method), suggesting that two warheads are largely independent for contributing toward inhibition. Taken together, we showed that we can use existing structural information to guide development of molecular bidents that inhibit a kinase by covalently reacting with two different cysteine residues located within the active site.

### Structure-Based Design of Potent Molecular Bident for EGFR

**Figure 3:**
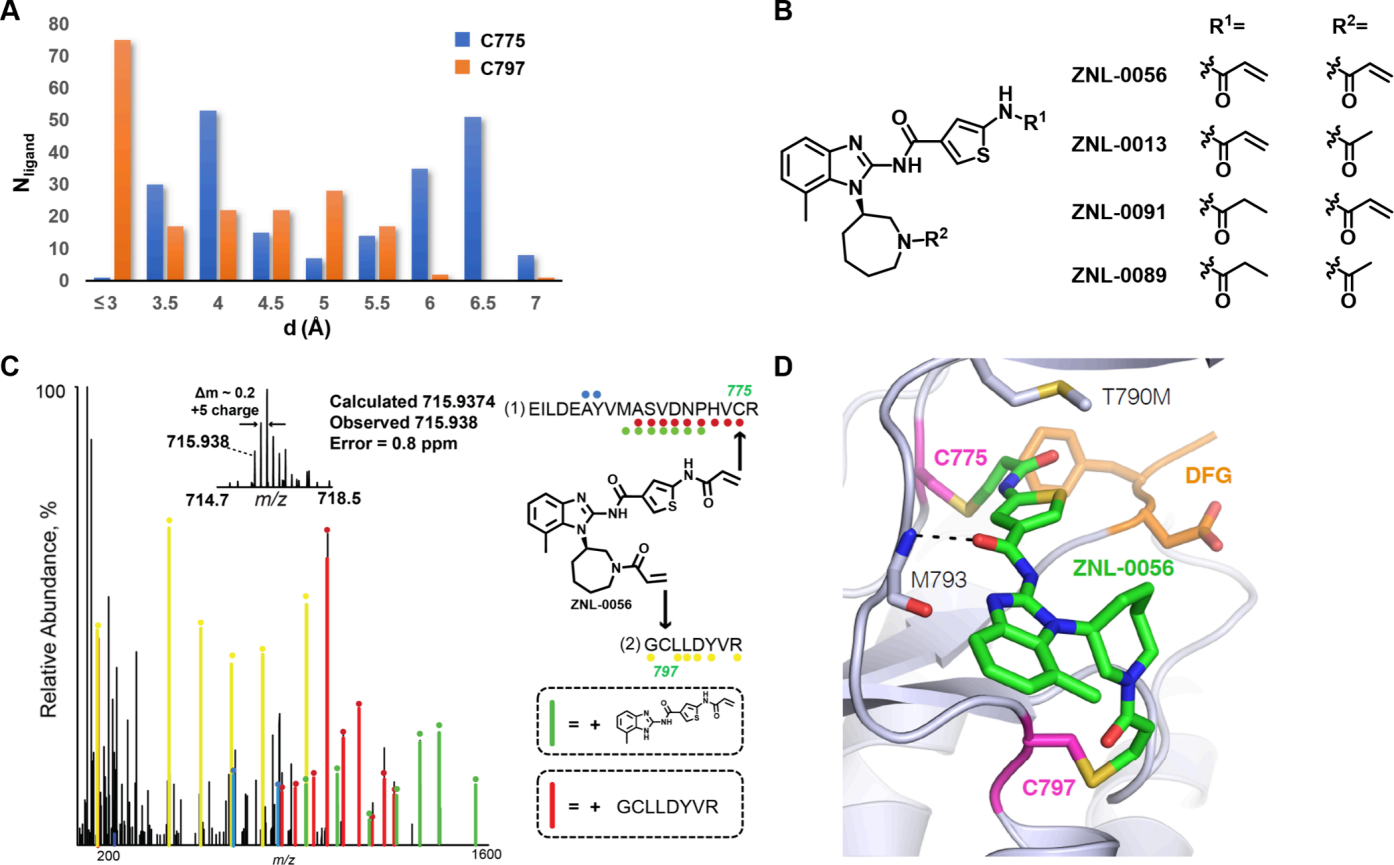
**ZNL-0056 is a molecular bident for EGFR**. (**A**) Count of cocrystal ligands to the respective residues at various distance between the ligands and cysteines. (**B**) Compound structures of ZNL-0056 (molecular bident), ZNL-0013 (front-warhead reversible compound), ZNL-0089 (double-warhead reversible compound), and ZNL-0091 (back-warhead reversible compound). (**C**) MS/MS spectrum of ZNL-0056 covalently modifying Cys 775 and Cys 797 of EGFR^L858R^. Ions of type y are shown with red and yellow circles for peptide sequences (1) and (2), respectively. Ions of type b are shown with blue circles for peptide sequence (1). Sequence ions containing a cleavage product of ZNL-0056 are shown with green circles (27). (**D**) ZNL-0056 co-crystal structure with EGFR^T790M/V948R^ (PDB 8EME).

To demonstrate the generality of the molecular bident approach, we applied it to the tyrosine kinase EGFR (Figure 1C). EGFR is an extensively studied therapeutic target especially in the context of non-small cell lung cancer (NSCLC) where mutations in EGFR such as the exon 19 and the single amino acid substitution L858R in exon 21 result in activation of EGFR and confer an “oncogenic” addiction to EGFR in the tumor cells. Covalent Cys797-targeting covalent inhibitors are a mainstay for the treatment of EGFR-dependent non-small cell lung cancer.^30,31^ In addition to clinically validated targeting of Cys797, our analysis showed that EGFR contains another cysteine residue (Cys775) potentially targetable using a molecular bident strategy. Cys775 is located in the back of the ATP pocket behind the gatekeeper residue Thr790 and resides in a restricted, less polar chemical microenvironment. Cys775 is less solvent accessible than Cys797 in EGFR and the cysteines in MKK7 and has not been targeted with covalent inhibitors to date^45^ (Figure S2A). Analysis of crystal structures of EGFR bound to Cys797-targeting covalent inhibitors provided suitable chemical starting points for introducing a second reactive warhead directed toward Cys775. As shown in Figure 3A, EGFR-inhibitor crystal structures showed a wide range of distance distributions between the bound ligand and Cys775 starting from 3 Å (measured by closest distance between heavy atoms of ligand and Cys775 e.g. in PDB 5GTY chain E).^32^ We also examined the orientation of the Cys775 side chain with respect to the ligand (Figure S2B) in order to identify the proper trajectory to enable a Michael addition reaction. Considering all these factors, the benzimidazole amide compound nazartinib^33,34^ emerged as one of the promising scaffolds for molecular bident design (Figure S2C). A virtual library of compounds containing the benzimidazole amide scaffold with various ring structures and warhead positions was designed and docked into the EGFR crystal structure (PDB: 5FED). This predicted that attaching acrylamides directly to the meta position of an aromatic ring would produce a compound that could reach Cys775 while maintaining the overall binding mode of the benzimidazole core. Representative molecules and docking score are shown in Figure S3.

To confirm the covalent bond formation with Cys797 and Cys775 by the compounds, we synthesized a set of such compounds (Figure S3) and tested their abilities to act as molecular bidents. We performed mass spectrometry with trypsin digestion of purified recombinant EGFR^L858R^ kinase domain treated by the compounds (Figure S3). Mass shifts consistent with the addition of single molecule to EGFR were observed indicating a single molecular adduct was formed. ZNL-0056 (Figure 3B) showed high *in vitro* labeling efficacy of both Cys797 and Cys775 of EGFR^L858R^ protein (Figure S3). Next, we were also able to identify two cross-linked peptides from Cys797 and Cys775, supporting the conclusion that ZNL-0056 forms simultaneous covalent bonds with both these residues intramolecularly (Figure 3C).

To visualize cross-linking, we determined a crystal structure of ZNL-0056 bound to the EGFR^T790M/V948R^ kinase domain at a resolution of 3.3 Å (Figure 3D, Table S1, Figure S4). The structure was obtained by soaking AMP-PNP crystals with ZNL-0056, and density for the inhibitor was only observed in one copy of the resulting structure. The structure revealed covalent bonds to both Cys797 and Cys775, though the electron density for the C775 adduct was weaker suggesting reduced occupancy (Figure S4A). As expected, the benzimidazole amide core forms a hydrogen bond with the backbone of Met793. The thiophene ring tucks beneath the gatekeeper residue Met790 to form hydrophobic interactions as well as a polarization interaction between the sulfur atom of Met790 and the Pi-system in the ligand. In summary, both mass spectrometry and cocrystal structure confirmed that ZNL-0056 targets both Cys775 and Cys797 in EGFR.

### ZNL-0056 Exhibits Covalency-Dependent Inhibition of EGFR Kinase Activity in Cells

**Figure 4:**
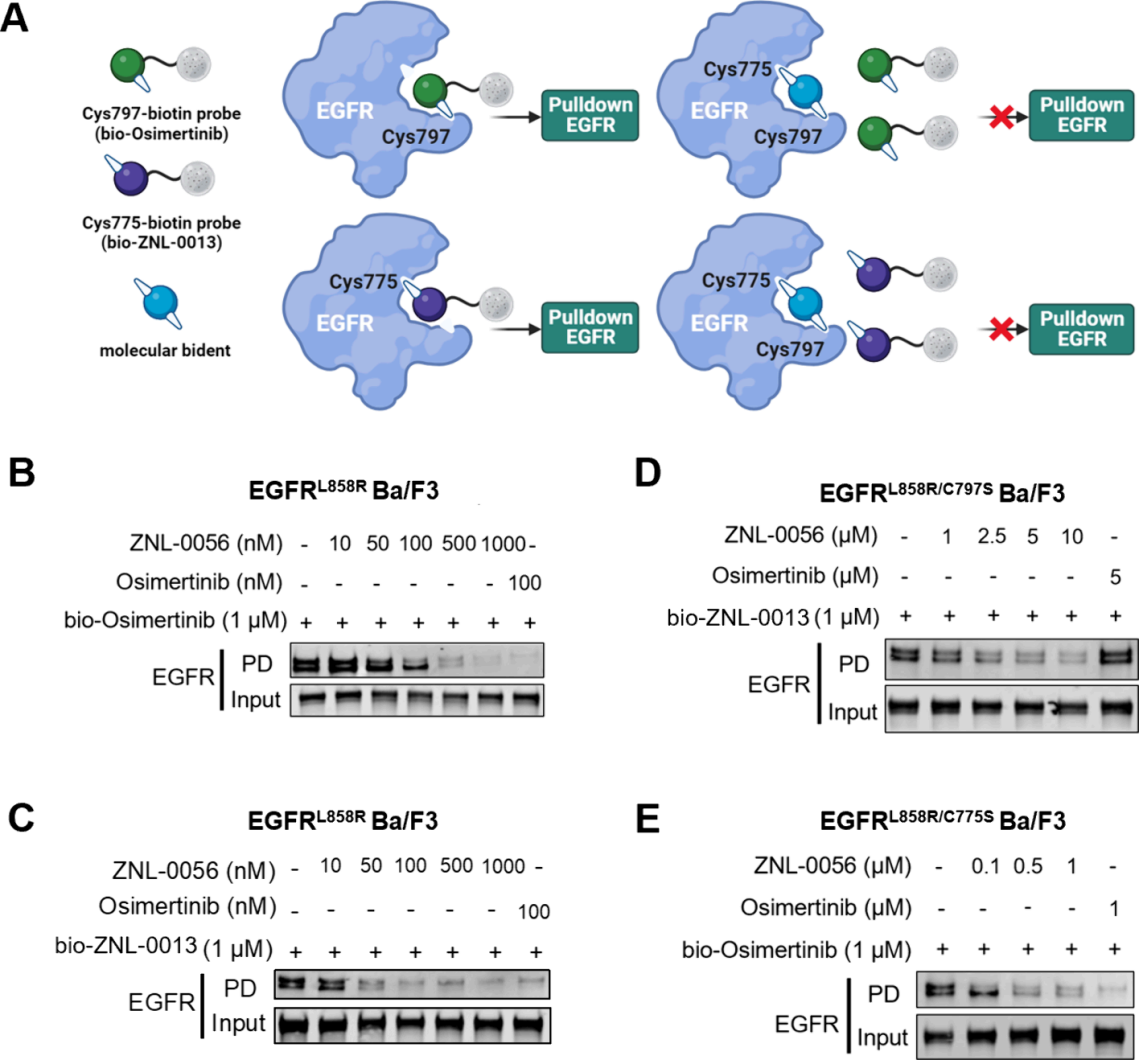
**ZNL-0056 covalently engages Cys775 and Cys797 in EGFR Ba/F3 cells**. (**A**) Carton of competition EGFR pulldown strategy to test covalent EGFR engagement in cells. (**B**) Competitive pulldown assay in EGFR^L858R^ Ba/F3 cells treated with ZNL-0056 at the indicated concentrations for 6 h. Cell lysates were incubated with bio-osimertinib. (**C**) Competitive pulldown assay in EGFR^L858R^ Ba/F3 cells treated with ZNL-0056 at the indicated concentrations for 6 h. Cell lysates were incubated with bio-ZNL-0013, which competes for the binding pocket of the target kinase. (**D**) Competitive pulldown assay in EGFR^L858R/C797S^ Ba/F3 cells treated with ZNL-0056 at the indicated concentrations for 6 h. Cell lysates were incubated with bio-ZNL-0013, which competes for the binding pocket of the target kinase. Western blotting showing the pulldown (PD) or input (total lysate) of EGFR. (**E**) Competitive pulldown assay in EGFR^L858R/C775S^ Ba/F3 cells treated with ZNL-0056 at the indicated concentrations for 6 h. Cell lysates were incubated with bio-osimertinib, which competes for the binding pocket of the target kinase. Western blotting showing the pulldown (PD) or input (total lysate) of EGFR.

We next examined the mode of target engagement in EGFR-mutated Ba/F3 cells. Pulldown experiments showed that pretreating cells with ZNL-0056 prevented EGFR interaction with a cysteine-directed EGFR probe (Figure 4A). For these experiments, we prepared non-cysteine-reactive analogues for either Cys797 (ZNL-0013), Cys775 (ZNL-0091), or both Cys797 and Cys775 (ZNL-0089) (Figure 3B). Pretreating cells expressing EGFR^L858R^ with ZNL-0056 prevented EGFR from binding to an osimertinib-biotin target-engagement probe^35^ in a dose-dependent manner (Figure 4B). Similarly, we developed a biotinylated probe of ZNL-0013 (bio-ZNL-0013; Figure S5) that was only capable of labeling Cys775 (Figure 4C). At a concentration of 1 μM, bio-ZNL-0013 showed significant enrichment of EGFR from cell lysate under denaturing conditions, indicating covalent bond formation.

We used these covalent probe compounds to test covalent target engagement. For this, we developed EGFR double mutant L858R/C797S (Figure 4D) and L858R/C775S (Figure 4E) cell lines to deconvolute the dependence of ZNL-0056 on Cys775 and C797S. In the presence of ZNL-0056, EGFR enrichment by probe bio-ZNL-0013 was reduced in the C797S mutant cells in a dose-dependent manner (Figure 4D). EGFR enrichment by the osimertinib-biotin probe was also diminished in the C775S mutant cells (Figure 4E). Taken together, these data showed that ZNL-0056 can outcompete covalent probes targeting Cys797 and Cys775, thus providing strong evidence that it covalently labels both Cys797 and Cys775 in EGFR mutant cells simultaneously.

### ZNL-0056 Demonstrates Antiproliferative Activity and Downstream Signaling in Mutant EGFR Ba/F3 Cells

**Figure 5:**
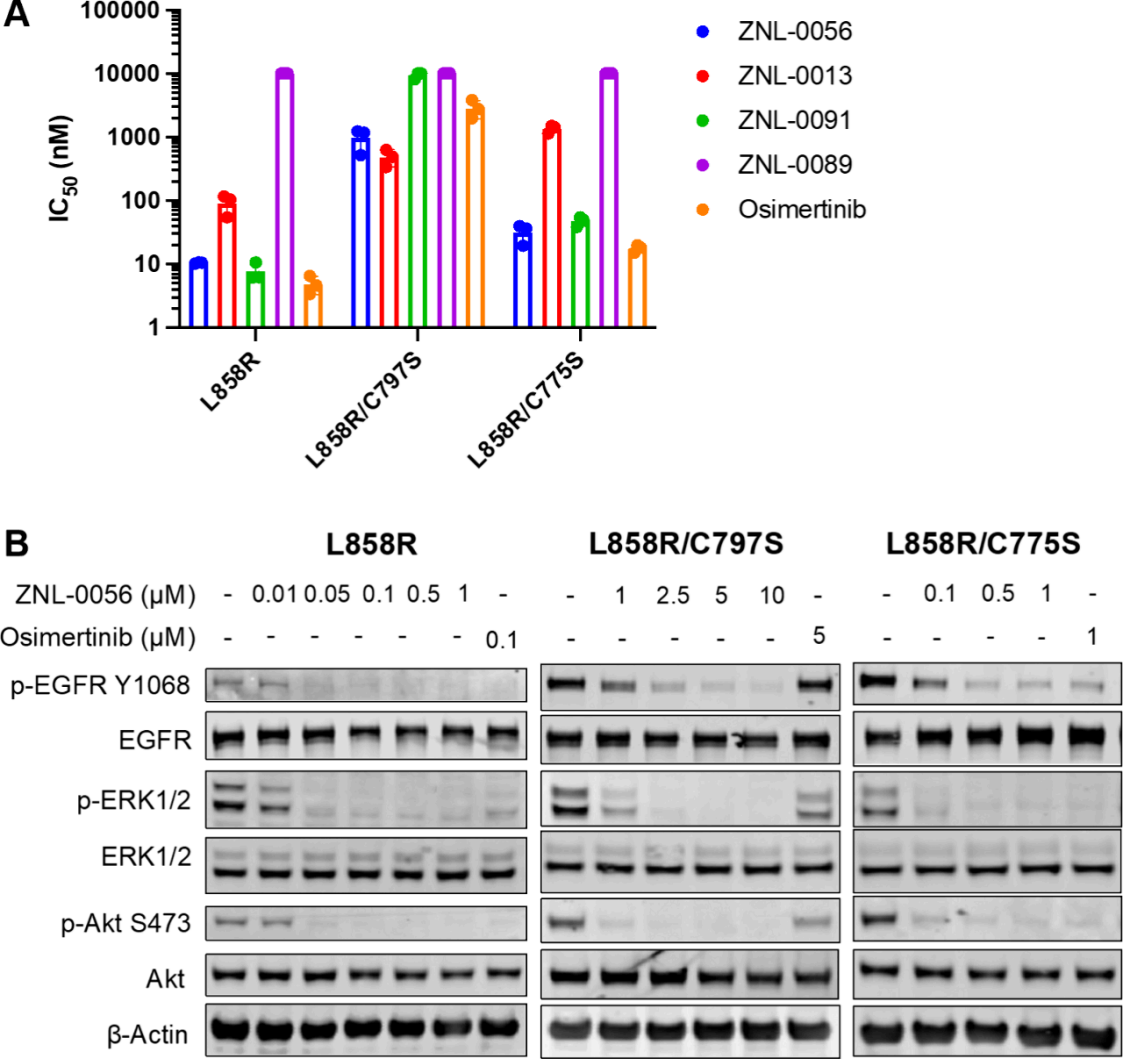
**ZNL-0056 covalently engages Cys775 and Cys797 in EGFR Ba/F3 cells**. (**A**) The average IC_50_ values (mean ± S.D.) of the indicated compounds in EGFR^L858R^ Ba/F3, EGFR^L858R/C797S^ Ba/F3, and EGFR^L858R/C775S^ Ba/F3 cells after 6 h treatment. The graph was generated from three independent experiments, each performed with four replicates. (**B**) Immunoblot assessment of the effect of ZNL-0056 on the EGFR signaling pathway in EGFR^L858R^ Ba/F3, EGFR^L858R/C797S^ Ba/F3, and EGFR^L858R/C775S^ Ba/F3 cells after 6 h treatment. Representative data from two independent experiments are shown.

We used EGFR-dependent cell lines to examine the antiproliferative activity of ZNL-0056 and its analogues. In EGFR^L858R^-dependent cells, ZNL-0056 had similar potency as ZNL-0091, whereas the cell growth inhibition activity of ZNL-0013 was about 5-fold weaker. Likewise, growth inhibition by ZNL-0056 was markedly different in EGFR^L858R/C775S^ and EGFR^L858R/C797S^ mutant cell lines (Figures 5A and S6). These results suggest that, though it engages EGFR as a molecular bident, ZNL-0056 reacts with its target cysteines with differing efficiencies, leading to differing dependencies on these residues for its cellular activity. Nevertheless, ZNL-0056 was superior to single warhead compounds such as ZNL-0091 and osimertinib when used in cells dependent on EGFR^C797S^ for growth; the activity loss for osimertinib and ZNL-0056 was 300- and 50-fold, respectively. Likewise, the single Cys775 targeting compound ZNL-0013 lost 20-fold potency in the EGFR^L858R/C775S^ mutant, while ZNL-0056 only shifted 2-fold. Therefore, the dual covalency of ZNL-0056 retains much of its cellular activity even when a single targeted cysteine is mutated, and this is not the case for traditional monovalent covalent inhibitors. It is noted that ZNL-0056 had similar activity as monovalent counterpart ZNL-0091 and ZNL-0013 in mutants where they can still form covalent bonds with the corresponding cysteine, i.e., ZNL-0091 in EGFR^L858R/C775S^ and ZNL-0013 in EGFR^L858R/C797S^. This is expected from the reciprocal combination equation derived from enzymatic inhibition kinetics (Materials and Methods). A fully optimized analogue of ZNL-0056 is expected to have an improved activity profile over monocovalent molecules. One of the key objectives for medicinal chemistry optimization for molecular bidents is to obtain a balanced covalent reactivity for two electrophiles in order to achieve maximal benefits.

To correlate the antiproliferative effects with on-target activity of ZNL-0056 against EGFR, we examined the phosphorylation levels of EGFR, ERK, and AKT upon compound treatment. The trends in covalent bond dependency seen in cell proliferation experiments discussed above matched the inhibition of EGFR, ERK, and AKT phosphorylation with ZNL-0056 in the EGFR^L858R^ Ba/F3 cells, EGFR^L858R/C797S^ Ba/F3 cells, and EGFR^L858R/C775S^ Ba/F3 cells (Figure 5B). Consistent with its effects on cell proliferation, ZNL-0056 significantly inhibited EGFR, ERK1/2, and Akt phosphorylation in all three EGFR-mutated Ba/F3 cells.

### ZNL-0056 Demonstrates Antiproliferative Activity in a Cancer Cell Line Derived from Primary Human Tumor Harboring the EGFR^L858R^ Mutation

**Figure 6:**
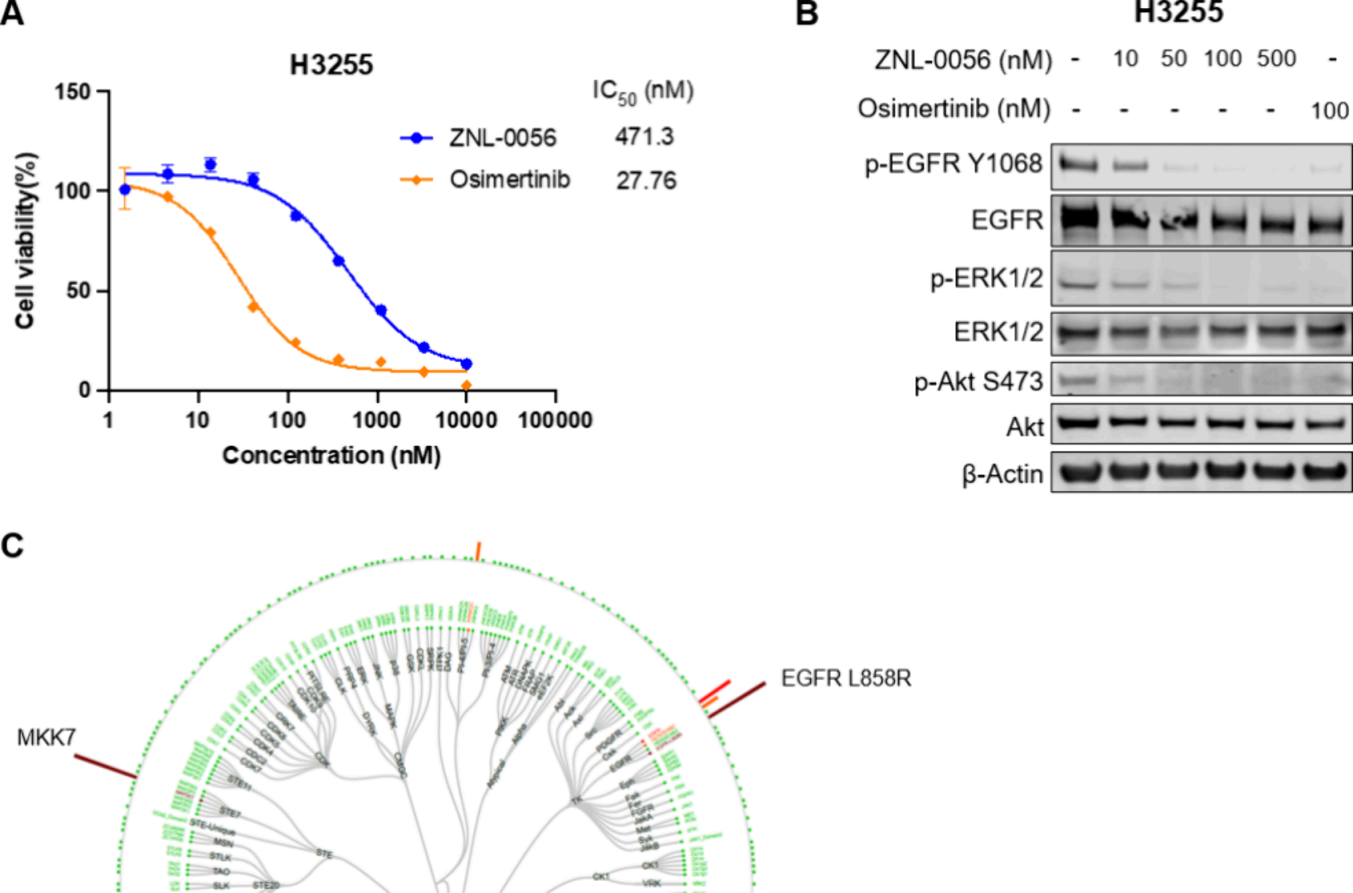
**ZNL-0056 selectively inhibits EGFR and its downstream signaling in H3255 cells**. (**A**) Dose–response curves for ZNL-0056 and osimertinib in H3255 cells following 72 h of treatment. Cell viability was assessed with CellTiter-Glo. Data are presented as the mean ± SEM (*n* = 4). Representative data from three independent experiments are shown. (**B**) Immunoblot assessment of the effect of ZNL-0056 on EGFR signaling pathway in H3255 cells after 6 h treatment. Representative blots from two independent experiments are shown. (**C**) KiNativ profiling in H3255 cell lysates treated with 2.5 μM of ZNL-0056 for 2 h.

H3255 is a human NSCLC cell line harboring the EGFR^L858R^ mutations.^36,37^ These cells also express other erythroblastic leukemia viral oncogene homologue (erbB) family kinases and other somatic mutations in cancer genes^38^ (https://cancer.sanger.ac.uk/cell_lines/sample/overview?id=1247873#muts accessed 2023–09–25) as well as drug efflux pumps and thus closely resemble primary human cancers. Cell viability assay showed that ZNL-0056 inhibited proliferation of H3255 cells at low micromolar concentrations (Figure 6A). The shift of potency from Ba/F3 cells is common due to multiple factors such as the different construct of EGFR and high expression of efflux drug pumps in human cancer cell lines.

Next, we examined the EGFR signaling pathway in H3255 cells. ZNL-0056 inhibited phosphorylation of EGFR, ERK1/2, and AKT phosphorylation at concentrations as low as 0.1 μM (Figure 6B). To evaluate the kinome-wide inhibition profile of ZNL-0056 in the context of human NSCLC cells, we performed activity-based proteomic profiling against 193 kinases using osimertinib as a reference compound. ZNL-0056 engaged EGFR^L858R^ and MKK7 in H3255 cells (Figures 6C, S7A, data file S3). In addition to this, we performed KINOMEscan profiling^39^ to evaluate *in vitro* kinase selectivity across a panel of 468 human kinases. EGFR and MKK7 were confirmed as targets (Figure S7B). Additional potential targets included BTK, BLK, JAK3, ITK, and MKK7. These targets all carry a cysteine analogous to EGFR^Cys797^, indicating ZNL-0056 likely labels this residue on multiple targets. Biochemical experiments confirmed that ZNL-0056 inhibited the activity of these targets (Table S2).

### ZNL-0056 Demonstrates Reasonable in Vivo Pharmacokinetic (PK) Profile in Mice

Even though covalent inhibitors have been traditionally perceived as intrinsically less stable and can potentially react with additional “off-target” proteins, acrylamide-containing covalent inhibitors have been developed into successful drugs including inhibitors of BTK, EGFR, and G12C KRAS such as ibrutinib, osimertinib, and sotorasib. As an unprecedented dual-warhead covalency strategy, it is critical to examine whether the second acrylamide inevitably decreases its stability in vivo.

To address this question, we tested the GSH reactivity of ZNL-0056 (Table S3) and its in vivo pharmacokinetic (PK) properties using three routes of administration including: 1 mg/kg IV, 3 mg/kg IP, and 10 mg/kg PO in male C57BL/6 mice (Table S4). While ZNL-0056 is relatively reactive toward conjugation with glutathione (GSH), its half-life of 36 min is similar to the half-life of 49 min measured for osimertinib measured in the same assay. ZNL-0056 had a short in vivo half-life of 0.33 h, which was likely due to poor metabolic stability, and low exposure with an area under the curve of 21 820 min·ng/mL following 1 mg/kg intravenous (IV) dose. However, a 3 mg/kg intraperitoneal (IP) dose of ZNL-0056 achieved an average maximal plasma concentration of 1.3 μmol/L and a bioavailability of 42%. At a 10 mg/kg oral (PO) dose, ZNL-0056 reached an average maximal plasma concentration of 1.4 μmol/L and promising exposure with an area under the curve of 63 341 min·ng/mL and exhibited a reasonableoral bioavailability of 24%. While ZNL-0056 does not have ideal PK properties, it showed promising potential to achieve desired metabolic and in vivo stability and is a suitable starting point for further medicinal chemical optimization.

## Conclusions

Traditionally, covalent inhibitors use a single warhead to target a specific nucleophilic residue, in a target protein. Here, we developed a systematic structure-based workflow to develop molecules that site-specifically target cysteine pairs located within the ligand binding pocket of a target. These molecular bident compounds provide a new modality for modulating target activity. Principally, the molecular bidents retain potency upon mutation of a single reactive cysteine. This provides a unique advantage for potential cancer therapeutics over typical covalent compounds.

Structural informatics analysis showed that many biologically important proteins can be potentially targeted by molecular bidents, and some of these are valuable drug targets. For example, among nonkinase targets, the WDR5 protein, which regulates transcription and is a cancer therapeutic target,^40^ has two cysteines within striking distance of some inhibitors, making it a potential candidate for site specific cross-linking. TP53, one of the most important tumor suppressors, also contains multiple cysteines in proximity to bound ligands. Therefore, based on the two demonstrated examples reported here and previously reported FGFR4, molecular bidents may represent a generalizable and practical strategy to target many proteins in the proteome.

We imagine that structurally designed molecular bidents may play different roles in regulating target functions for different proteins. While it is possible to confer covalent drug resistance through mutations other than covalent bonded nucleophilic residues and bypass mechanisms, bispecific covalent compounds are likely to produce selective pressures for different sets of resistant mutations. This is an active research area being pursued. In the current study, ZNL-8162 was shown to have two electrophiles that are equally efficient to react with respective cysteines in MKK7. As a result, it retained high potency despite the loss of one covalent bond due to mutation of the cysteine residue.

In addition to resistance prevention, molecular bidents may provide a route to achieve enhanced selectivity. This has previously been demonstrated for monovalent covalent kinase inhibitors.^41^ ZNL-0056, which engages both the well-studied Cys797 and unexplored Cys775, showed great selectivity in H3255 cells despite an additional electrophilic reactive group. Unlike ZNL-8162 for MKK7, the unbalanced contribution from the two warheads in ZNL-0056 presented a noticeable drawback as a lead molecule. We believe that two key factors were responsible for the observed differences. First, Cys775 is intrinsically less reactive with a more restricted access vector than Cys797 as discussed above. Second, the benzyl imidazole scaffold has been extensively optimized for labeling Cys797, and further optimization or an alternative scaffold is needed to achieve the comparable contributing profile on Cys775. In therapeutic settings where resistance mutations do not produce a selective advantage (i.e., nonproliferative diseases), one can harness the first covalent bond to increase the residence time to enable the covalency for the second covalent bond formation where the second cysteine is functionally important but difficult to target. Therefore, the desired reactivity profile depends on target and disease relevance.

In the current study, we demonstrate the concept of molecular bidents through cysteine modification. This approach can be extended to other nucleophilic residues; lysines, histidines, serines, threonines, and tyrosines are all substrates for chemical covalent modification.^42,43^ Doing so will greatly expand the number of potential targets for new molecular bidents. For example, structural analysis of ligand bound BTK reveals that in addition to Cys481, which forms a covalent bond to several BTK covalent inhibitors including ibrutinib, this kinase contains several additional residues (Tyr476, Ser538, Thr474, and the catalytic Lys430) that are in relevant proximity to the bound ligand (PDB: 7L5P).^44^ Molecular bidents targeting BTK and other similar drug targets might be achievable even in the absence of multiple suitable nearby cysteines. Another aspect of the molecular bident concept is its potential to modify the conformational stability of a target. Conceptually, molecular bident compounds may have an effect analogous to the role of disulfide bonds in protein folding. Going forward, these intermolecular bidents could enable on-demand formation of neo-complexes to modify protein function, substrate binding, half-life, and localization. Our study presents evidence that molecular bidents represent a new strategy to explore the functional consequence of modulating proteins of interests.

## Data Availability

All data and code to understand and assess the conclusions of this research are available in the main text, Supporting Information, and the Protein Data Bank via accession codes 8EME.

## Supporting Information Available

A detailed method section as well as all of the supplemental figures and tables referenced in the manuscript (PDF)
Data file S1: The available protein-ligand complex structures with multiple cysteines in the binding pocket (XLSX)
Data file S2: The complete list of targets amendable to molecular bidents (XLSX)
Data file S3: ZNL-0056 and Osimertinib of KiNativ profiling in H3255 cell lysates (XLSX)
Data file S4: The sequences of EGFR and MKK7 (PDF)
