# Supplementary material for: Molecular Bidents with Two Electrophilic Warheads as a New Pharmacological Modality
Source: ACS Cent Sci. 2024 Feb 26;10(6):1156–66. doi: 10.1021/acscentsci.3c01245 (PMC11212140; doi:10.1021/acscentsci.3c01245)
Supplement: Supplementary file 1 — oc3c01245_si_001.pdf [file oc3c01245_si_001.pdf]

Supporting Information

Molecular bidents with two electrophilic warheads as a new pharmacological modality

Zhengnian Li,[#] Jie Jiang,[#] Scott B. Ficarro, Tyler S. Beyett, Ciric To, Isidoro Tavares, Yingde Zhu, Jiaqi Li, Michael J. Eck, Pasi A. Jänne, Jarrod A. Marto, Tinghu Zhang*, Jianwei Che*, Nathanael S. Gray*

The file includes:

Figures S1 to S7
Tables S1 to S4
Materials and Methods
References
NMR spectra

Other Supplementary Materials for this manuscript include the following:

Data file S1 to S4

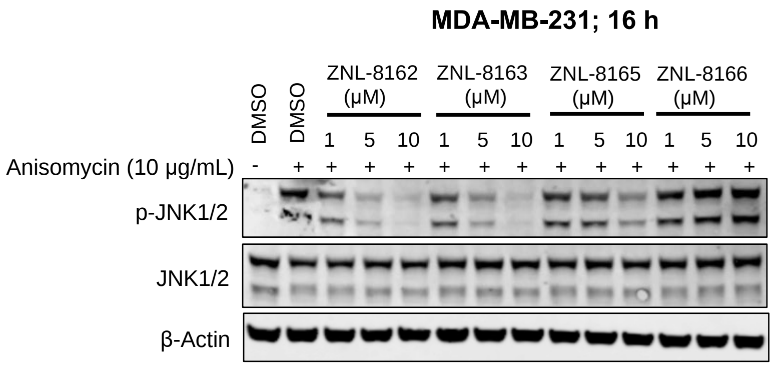

Figure S1. Effects of the indicated compounds on the MKK7-JNK pathway in MDA-MB-231 cells after 16 h treatment. MDA-MB-231 cells were pretreated with anisomycin for 45 min prior to the addition of the indicated compounds for 16 h. Representative data from two independent experiments are shown.

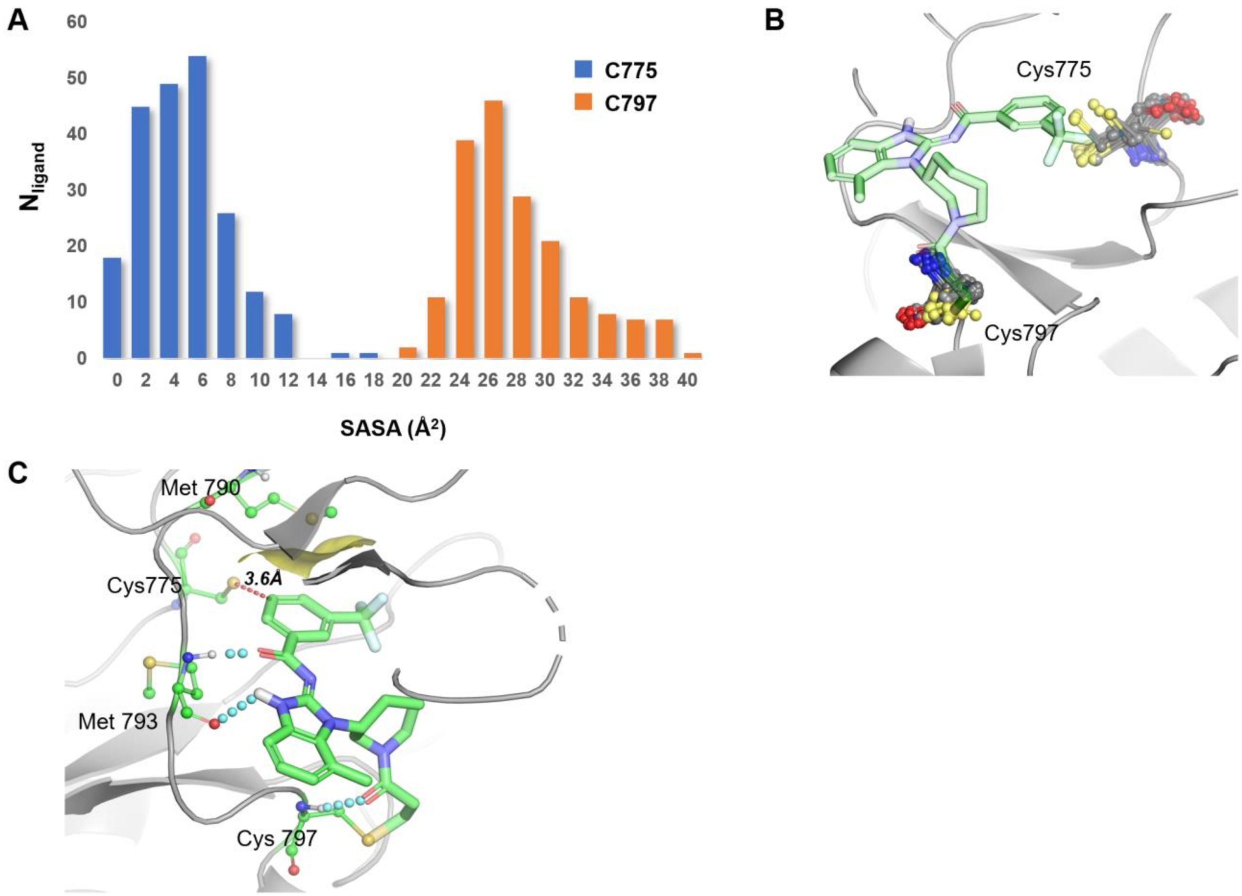

Figure S2. Cysteine map and Cysteines in the EGFR.

(**A**) Solvent accessible surface area (SASA) for C775 and C797 in co-crystal structures of EGFR. (**B**) Rotameric states of Cys775 and Cys797 in various crystal structures. (**C**) Crystal structure of human EGFR^{T790M} in complex with Nazartinib analog (5FEE), the distant between cys775 and phenyl ring highlight with 3.6Å.

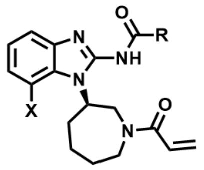

Compound ID	R	X	Cys797 (%)	Cys775 (%)	Docking score[a]
ZNL-0245	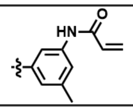	Methyl	62 ± 1	69 ± 2	-8.8
ZNL-0102	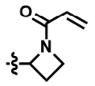	Cl	21 ± 9	67 ± 7	-5.4[b]
ZNL-0178	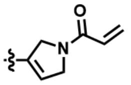	Cl	66 ± 4	49 ± 13	-7.3
ZNL-0166	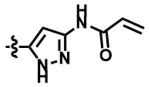	Cl	66 ± 5	51 ± 1	-6.5
ZNL-0056	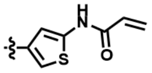	Methyl	71 ± 6	81 ± 6	-6.3

Figure S3. Structures of crosslinking compounds, covalent docking score, and MS digestion for each Cysteine in EGFR^{L858R}.

Digestion data are presented as mean ± s.e.m. of n ≥ biologically independent samples. [a] The covalent docking studies were performed in EGFR (PDB: 5FED) with representative compounds, and the docking scores were for covalent docking to Cys775 calculated by CovDock from Schrödinger. [b] The score is for R form, S isomer scored less

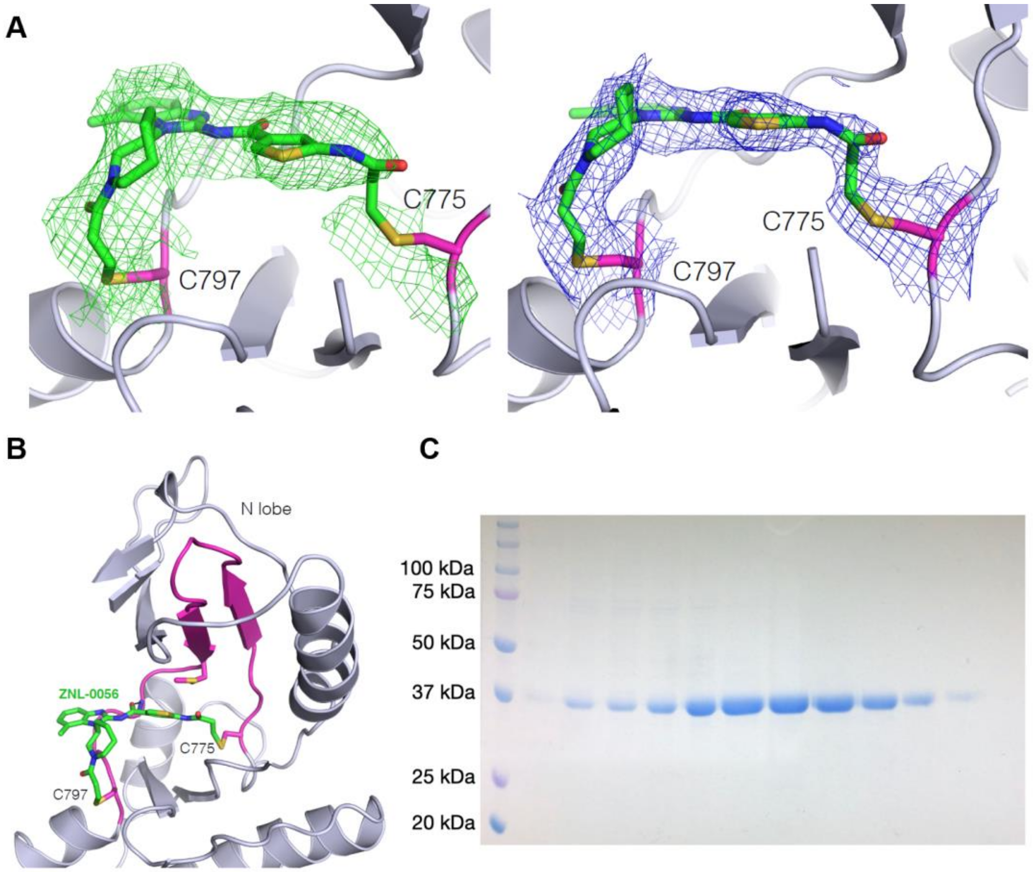

Figure S4. Cocrystal of ZNL-0056 with EGFR$^{T790M/V948R}$.

(**A)** Electron density for ZNL-0056. The left panel shows green positive F_o-F_c omit map density at 2.5 σ. The right panel shows blue refined $2F_o$-F_c electron density at 1 σ. (**B**) Depiction of intramolecular crosslinking within the N lobe of the kinase domain. The peptide segment between C775 and C797 is shown in magenta. (**C**) SDS-PAGE of purified EGFRT790M/V948R used to grow crystals with ZNL-0056.

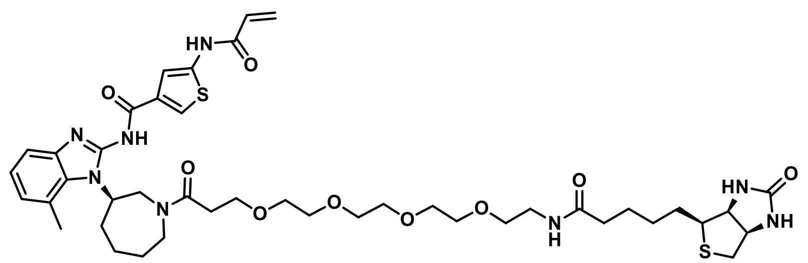

bio-ZNL-0013

Figure S5. Structure of biotinylated ZNL-0013 probe.

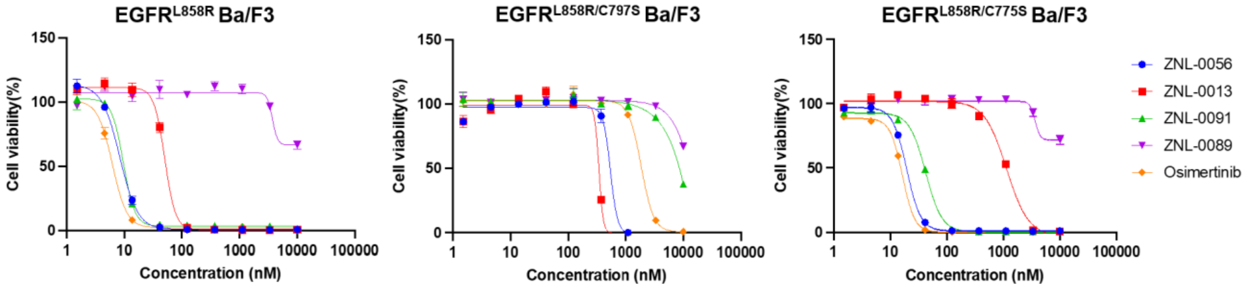

Cmpd ID	L858R IC$_{50}$ (nM)	L858R/C797S IC$_{50}$ (nM)	L858R/C775S IC$_{50}$ (nM)
ZNL-0056	10.25	516.2	19.4
ZNL-0013	54.36	335.2	1135
ZNL-0091	10.62	8162	38.64
ZNL-0089	>10000	>10000	>10000
Osimertinib	6.527	1964	15.1

Figure S6 Dose-response curves for the indicated compounds in EGFR^{L858R}, EGFR$^{L858R/C797S}$, and EGFR$^{L858R/C775S}$ Ba/F3 cells following 72 h of treatment. Cell viability was assessed with CellTiter-Glo. Data are presented as the mean ± SEM (n = 4). Representative data from three independent experiments are shown.

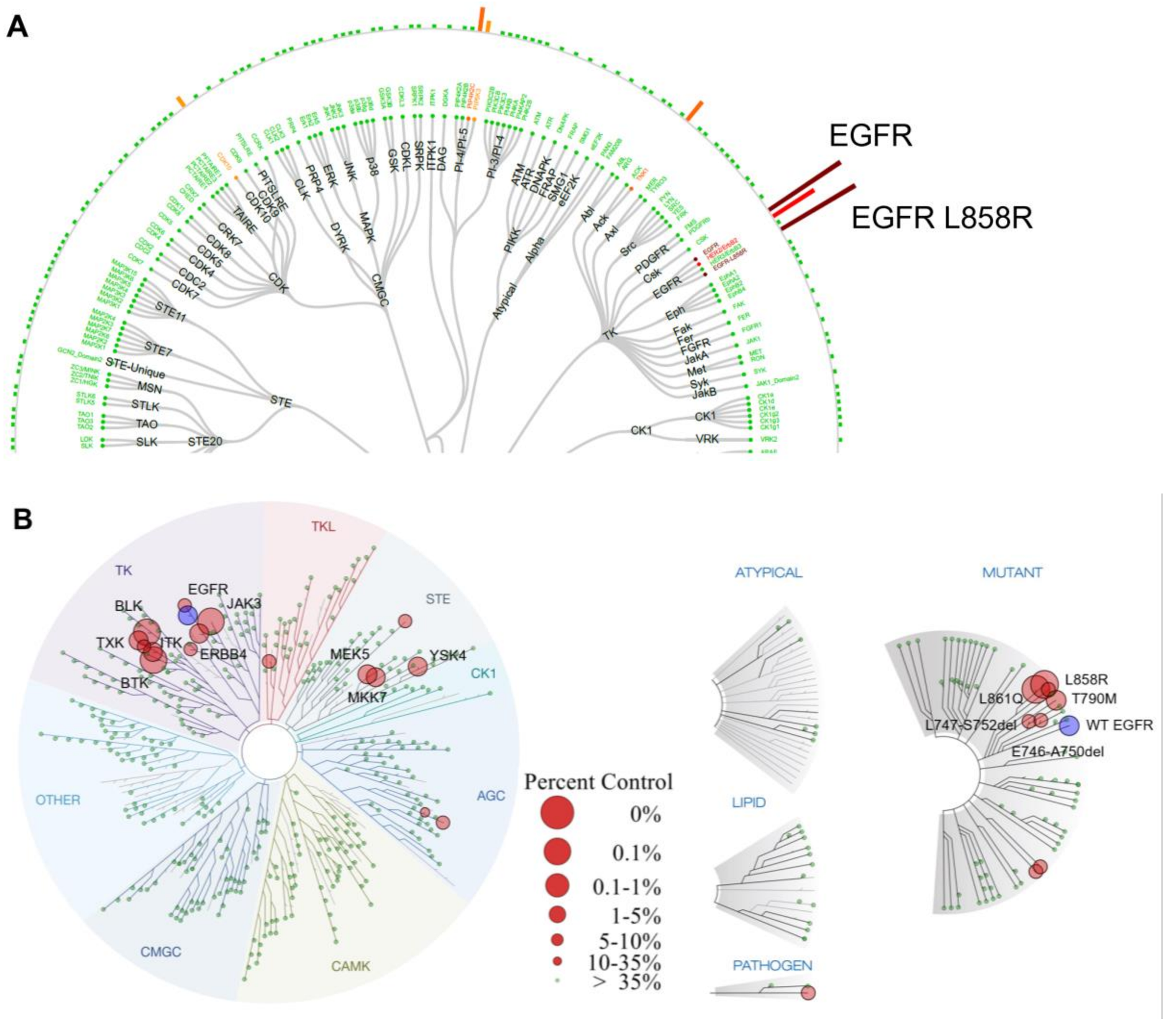

Figure S7. Selectivity profiling of osimertinib and ZNL-0056

A) KiNativ profiling in H3255 cell lysates treated with 2.5 μM of osimertinib for 2 hours. B) KinomeScan profiling for ZNL-0056 at 1 μM against a panel of 468 human kinases. The results for the binding interactions are reported as "% Ctrl", where larger circles indicate stronger hits. The selectivity score was defined as the ratio of the number of kinases inhibited to a specified percentage versus the total number of kinases. For this experiment, specified percent inhibition was set at 10%, resulting in S (10) values of 0.05 for ZNL-0056.

Table S1. Data collection and refinement statistics

PDB Accession	8EME
**Data Collection**	
**Resolution range**	50 - 3.32 (3.439 - 3.32)
**Space group**	P 1 2_1 1
**Unit cell**	
**a, b, c (Å)**	34.87 99.99 87.02
**α, β, γ (°)**	90 100.8 90
**Total reflections**	33205 (3427)
**Unique reflections**	8668 (876)
**Multiplicity**	3.8 (3.9)
**Completeness (%)**	98.73 (98.86)
**Mean I/sigma(I)**	3.61 (0.71)
**Wilson B-factor**	87.37
**R-merge**	0.2971 (1.542)
**R-meas**	0.3457 (1.788)
**R-pim**	0.1742 (0.8919)
**$CC_{1/2}$**	0.976 (0.635)
**Refinement**	
**Reflections used in refinement**	8634 (868)
**Reflections used for R-free**	497 (48)
**R_{work}**	0.2837 (0.3633)
**R_{free}**	0.3150 (0.3745)
**Number of non-hydrogen atoms**	4461
**macromolecules**	4427
**ligands**	34
**solvent**	0
**Protein residues**	549
**RMS (bonds)**	0.003
**RMS (angles)**	0.67
**Ramachandran favored (%)**	91.93
**Ramachandran allowed (%)**	6.38
**Ramachandran outliers (%)**	1.69
**Rotamer outliers (%)**	1.43
**Clashscore**	6.47
**Average B-factor**	101.4
**macromolecules**	101.6
**ligands**	73.7

Statistics for the highest-resolution shell are shown in parentheses.

Table S2. Biochemical activities against a panel of kinase targets

	ZNL-0056
[a] EGFR ^{L858R}	6.21
JAK3	5.79
BTK	1.13
BLK	3.17
ITK	7.81
[b] MEK5	2500
[c] MKK7	36

[a]IC_{50} (nM) against EGFR ^{L858R}, BTK, JAK3, ITK and BLK were obtained with Z'-LYTE biochemical assay. [b]IC_{50} (nM) against MEK5 was obtained with LanthaScreen Binding activity assay. [c] Kd (nM) of MKK7 were obtained from KdELECT assays (DiscoverX).

Table S3. Glutathione (GSH) reactivity of ZNL-0056

Compound ID	GSH Reaction Assay				
	R^2	$T_{1/2}$ (min)	Remaining (T=1440min)	Remaining (*NGSH=1440min)	Glutathione conjugation (Aa at T1440)
ZNL-0056	0.9044	35.6	0.4%	99.7%	5190
Osimertinib	0.9969	48.9	0.3%	104.5%	12605718
Afatinib	0.9761	33.3	0.8%	105.8%	2743618

*NGSH1440: the abbreviation of no glutathione. No glutathione is added into NGSH sample (replaced by buffer) during the 24 hour-incubation R^2 is the correlation coefficient of the linear regression for the determination of kinetic constant.

Table S4. Effect of ZNL-0056 *in vivo*- PK Parameters

Subject	$T_{1/2}$	T_{max}	C_{max}	C_{mpax}	AUC_{last}	AUC_{last}	AUC_{INF_obs}	AUC	Cl_obs	MRT_{INF_obs}	Vss_obs
	hr	hr	ng/mL	μM	min*ng/mL	μM.hr	min*ng/mL	%Extrap	mL/min/kg	hr	L/kg
IV Mouse-1	0.32	0.08	505	1.06	11461	0.40	11559	0.84	86.5	0.30	1.56
IV Mouse-2	0.33	0.08	1280	2.68	29612	1.03	29913	1.01	33.4	0.32	0.64
IV Mouse-3	0.32	0.08	1110	2.33	24386	0.85	24566	0.73	40.7	0.28	0.69
Avg.	0.33	0.08	965	2.02	21820	0.76	22013	0.86	53.6	0.30	0.96

Subject	$T_{1/2}$	T_{max}	C_{max}	C_{max}	AUClast	AUC_{last}	AUC_{INF_obs}	$AUC_{\%Extrap}$	Cl_obs	F%	CL/F
	hr	hr	ng/mL	μM	min*ng/mL	μM.hr	min*ng/mL		mL/min/kg		
IP Mouse-4	0.49	0.25	668	1.40	33201	1.16	33325	0.37	90.0	50.7	45.7
IP Mouse-5	0.41	0.08	913	1.91	37472	1.31	37521	0.13	80.0	57.2	45.8
IP Mouse-6	0.56	0.25	251	0.53	11523	0.40	11600	0.67	258.6	17.6	45.5
Avg.	0.49	0.19	611	1.28	27398	0.96	27482	0.39	142.9	41.9	45.7

Subject	$T_{1/2}$	T_{max}	C_{max}	C_{max}	AUClast	AUC_{last}	AUC_{INF_obs}	$AUC_{\%Extrap}$	Cl_obs	F%	CL/F
	hr	hr	ng/mL	μM	min*ng/mL	μM.hr	min*ng/mL		mL/min/kg		
PO Mouse-7	3.3	0.08	565	1.18	47838	1.67	57756	17.2	173.1	21.9	38.0
PO Mouse-8	5.6	0.08	786	1.65	53685	1.88	69902	23.2	143.1	24.6	35.2
PO Mouse-9	2.5	0.25	623	1.31	58135	2.03	62367	6.8	160.3	26.6	42.7
Avg.	3.8	0.14	658	1.38	53219	1.86	63341	15.7	158.8	24.4	38.6

Dose: 1mg/Kg IV, 3mg/Kg IP, 10mg/Kg PO. Formulation: 0.1mg/mL(IV) and 0.3mg/mL (IP) and 1mg/mL (PO) solution in 5/25/70 EtOH/PEG300/D5W. Mice: C57Bl/6 male

Materials and Methods

Cell culture

H3255 were cultured in ACL-4 media[1] containing 10% fetal bovine serum (GeminiBio, Cat #100-106), 100 units/mL Penicillin and 100 μg/mL Streptomycin (gibco, cat #15140-22). The EGFR mutant L858R and L858R/C797S Ba/F3 cells were generated and characterized as described previously[2], and cultured in in RPMI media (Life technologies, Cat# 11875119) containing 10% fetal bovine serum, 100 units/mL Penicillin and 100 μg/mL Streptomycin. All the cell lines were cultured at 37°C in 5% CO_2 humidified air and tested for mycoplasma negative.

Generation of $EGFR^{L858R/C775S}$ Ba/F3 cells

Oncogenic kinase transformed Ba/F3 cell lines were established as previously described[3]. In brief, 3 μg of $EGFR^{L858R/C775S}$ expression vector was co-transfected with 3 μg of psPAX2 and 0.3 μg of pMD2.G (the packaging plasmids) into 70%-80% confluent 293T cells maintained in 3 mL of RPMI in a 60 mm dish by Lipofectamine 2000 (Thermo Fisher Scientific, cat# 11668-019) mediated transfection. 48 hours post-transfection, the viral supernatant was harvested and filtered with 0.45 μm membrane.

For infection of Ba/F3 cells, 2.5 mL of viral supernatant and 8 μg/mL polybrene was added to 2.5 million cells per well in six-well plates. The plate was centrifuged at 2500 rpm for 90 minutes at 37 ˚C for the spin infection. Then, virus-containing medium was removed 48 hours later and successfully infected cells were selected with the addition of 1 μg/mL puromycin to the medium. After 3 days, cells were transferred into the fresh medium without IL-3 and puromycin. IL-3-independent transformed cells were maintained in RPMI medium 1640 supplemented with 10% FBS. Expression of EGFR was examined by Western blot.

Immunoblotting and antibodies

Cells were lysed in RIPA buffer (150 mM NaCl, 1.0% IGEPAL® CA-630, 0.5% sodium deoxycholate, 0.1% SDS, 50 mM Tris, pH 8.0) (Sigma, Cat# R0278) with protease inhibitor and phosphatase inhibitor (Roche). The protein concentrations were measured by BCA analysis (Thermo Fisher Scientific, Cat # PI23225). Equal amounts of protein were resolved by 4-12% Bis-Tris gels (Life Technologies), and then transferred to the Immuno-Blot PVDF membrane (BioRad, cat # 1620177). The membranes were blocked by Intercept blocking buffer (LI-COR, cat #927-60001) for 1 hour at room temperature. Proteins were probed with appropriate primary antibodies at 4 °C overnight and then with IRDye®800-labeled goat anti-rabbit IgG (LICOR Biosciences, cat # 926-32211), IRDye®800-labeled goat anti-mouse IgG (LICOR Biosciences, cat # 926-32210) or IRDye 680RD goat anti-Mouse IgG (LICOR Biosciences , Cat # 926-68070) secondary antibodies at room temperature for 1 hour. The membranes were detected on Odyssey CLx system.

Antibodies used in this study include anti-following proteins: EGFR (Cell signaling Technology, 4267S, 1:1000), p-EGFR (Tyr1068) (Cell signaling Technology, #3777S, 1:1000), ERK1/2 (Cell signaling Technology, 4696S, #1:0000), p-ERK1/2 (Cell Signaling Technology, 4370S, 1;1000), Akt (Cell signaling Technology, # 9272L, 1:1000), p-Akt (Cell Signaling Technology, #4060S, 1;1000) and β-Actin (Cell Signaling Technology, #3700, 1:1000), p-JNK1/2 (Cell Signaling Technology, 9251S, 1:1000), JNK1/2 (Cell Signaling Technology, 9252S, 1:1000).

Antiproliferation assay

Cells were seeded at the density of 1000 cells/well for 384-well plates. For adherent cell lines, cells were cultured for 16 hours before compounds were added into the media for 72 hours

treatment. Cell viability was determined by using CellTiter-Glo (Promega #G7571) according to the manufacturer's instructions, measuring luminescence using an Envision plate-reader (PerkinElmer Inc.). Dose-response curves were generated using non-linear regression curve fit in GraphPad Prism 9.2.0(GraphPad Software).

Cellular target engagement assays

After 6 hours treatment, cells were pelleted, washed with PBS once and lysed with IP lysis buffer (25mMTris-HCl pH 7.4, 150mM NaCl, 1 mM EDTA, 1% NP-40 and 5% glycerol) (Thermo Fisher Scientific, Cat#87788) containing protease/phosphatase inhibitor cocktail (Roche). The protein concentrations were measured by BCA analysis (Pierce). Cell lysates were incubated with 1 μM of biotin conjugated probe (bio-Osimertinib or bio-ZNL-0013) at 4 ℃ overnight, and incubated for 3 more hours at room temperature. Lysates with probe were then incubated with streptavidin beads (Thermo Fisher, #20349) for 2 hours at 4 ℃. The protein-probe complexes on the beads were then subjected to immunoblotting.

KiNativ Profiling

H3255 cells were plated with $6.5x10^{\wedge 6}$ per condition in fresh media in 15 cm plates and treated the 80% confluency for 2 hours with DMSO or 2.5 μM of ZNL-0056. To harvest cells, plates were washed 3 times with cold PBS, then collected by scraping into 4 mL of cold PBS containing protease and phosphatase inhibitors. Cells were pelleted by centrifugation at 1350 g at 4 ℃ for 5 minutes and immediately frozen in liquid nitrogen. The frozen samples were sent to ActivX Biosciences (La Jolla, CA) for the remainder of the KiNativ profiling experiment[4].

Protein expression and purification

Human EGFR kinase domain (residues 696-1022) with T790M and V948R mutations was cloned with an N-terminal, protease cleavable His-GST tag. Recombinant protein was expressed in SF9 insect cells using baculovirus generated from the pFastBac system (Thermo Fisher). Briefly, cells at a density of ~$2x10^6$ cells/mL were infected with 1% (v/v) virus stock and grown for ~72 hours before harvesting and freezing the cell pellet. Infected cells were lysed in buffer A (50 mM Tris pH 8.0, 500 mM NaCl, 5% glycerol, 0.5 mM Tris(2-carboxyethyl)phosphine [TCEP]), ultracentrifuged at >200,000 rcf for 1 h, and the supernatant flowed through Ni-NTA resin by gravity. The resin was washed with buffer A supplemented with 40 mM imidazole by

flowing through the gravity column. EGFR kinase was eluted with buffer A containing 200 mM imidazole by flowing through the gravity column. A stoichiometric excess of TEV protease that was purified in-house was added to the eluted protein and dialyzed overnight at 4 ºC in buffer A. Following cleavage, kinase domain was obtained by flowing the mixture through Ni-NTA resin before purification by size exclusion chromatography with an S200 column (Cytiva) in buffer A. Eluted protein with purity ≥95% by SDS-PAGE was concentrated to ~3 mg/mL.

Structure determination

Purified $EGFR^{T790M/V948R}$ kinase domain at ~3 mg/mL was incubated with 10 mM $MgCl_2$ and 1 mM adenosine 5′-(β,γ-imido)triphosphate (AMP-PNP). The protein was crystallized using the hanging drop vapor diffusion method above wells containing 0.1 M Bis-Tris pH 5.5 and 28-30% (w/v) PEG 3350. Crystals formed in 1-3 days at room temperature. Crystals were soaked overnight with 0.5 mM ZNL-0056 before being cryoprotected in well solution supplemented with 30% ethylene glycol and flash frozen. Diffraction data were collected at 100 K at the Advanced Photon Source at the Argonne National Lab using beamline ID-24-E. Data were indexed, integrated, scaled, and merged using xia2 and Dials[5, 6]. The structure was phased by molecular replacement in Phaser using an inactive conformation EGFR kinase structure from the PDB[7, 8]. Model building was accomplished using Coot and iterative refinements were performed with Phenix Refine[7, 9]. The final structure has been deposited in the Protein Data Bank with the accession code 8EME.

Estimation of IC_{50} for ZNL-8162 based on independent covalent reactions

Assuming the covalent reactions for both warheads can proceed independently after reversible complex formation as illustrated below,

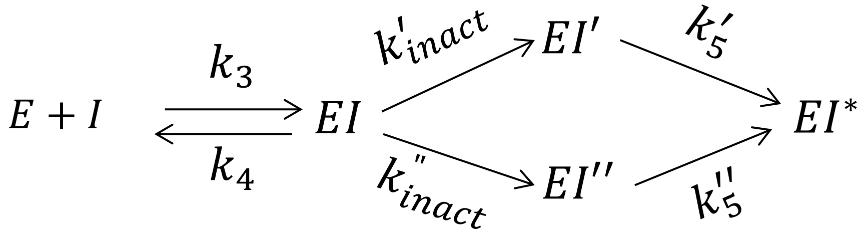

(1)

where k_3 and k_4 are the forward and reverse rate constant, respectively, for EI which represents the reversible complex. k'_{inact} and k''_{inact} represent the rate constants for forming the front and back covalent bonds as the first step, respectively, and k'_5 and k''_5 represent the rate constants for forming the second covalent bond following either the front or the back covalent bond formation.

EI* represents the final complex where both front and back covalent bonds are formed. For the above inhibition diagram, the rate equations are,

$$\frac{d[EI]}{dt} = k_3[E][I] - k_4[EI] - k'_{inact}[EI] - k''_{inact}[EI] \quad (2)$$

$$\frac{d[EI']}{dt} = k'_{inact}[EI] - k'_5[EI'] \quad (3)$$

$$\frac{d[EI'']}{dt} = k''_{inact}[EI] - k''_5[EI''] \quad (4)$$

$$\frac{d[EI^*]}{dt} = k'_5[EI'] + k''_5[EI''] \quad (5)$$

Defining $[EI^{\emptyset}] = [EI'] + [EI''] + [EI^*]$, which represents the total concentration of inactivated enzyme inhibitor complex, we arrive at,

$$\frac{d[EI^{\emptyset}]}{dt} = k'_{inact}[EI] + k''_{inact}[EI] \quad (6)$$

As one can expect intuitively, the inactivated complex depends only on k'_{inact} and k''_{inact} because the second covalent bond formation does not modify the activity of the complex. It only shifts the concentrations of the intermediate complexes EI' and EI'' towards final EI^*, and the total inactivated $[EI^{\emptyset}]$ depends only on the influx from $[EI]$. Therefore, the inhibition diagram is equivalent to a standard two step irreversible inhibition where $EI^{\emptyset}$ represents the collective species of irreversible complexes.

$$E + I \underset{k_4}{\overset{k_3}{\rightleftarrows}} EI \xrightarrow{k_{inact}} EI^{\emptyset} \quad (7)$$

Where $k_{inact} = k'_{inact} + k''_{inact}$. All the standard single warhead irreversible inhibitor formulation can be applied straightforwardly, where one can view the two pathway covalency provides independent conduits to inactivate the complex, and the outflux of reversible complex EI toward irreversible complex is the sum of two pathways.

In the case where reversible complex forms much faster than covalent complex, the steady states of $[EI]$ satisfies the following equation,

$$\frac{[E][I]}{[EI]} = \frac{k_4 + k_{inact}}{k_3} = k_i \quad (8)$$

$$\frac{k_{inact}}{k_i} = \frac{k_3 k_{inact}}{k_4 + k_{inact}} \quad (9)$$

Although IC_{50} is a time dependent variable for irreversible inhibitors, it has been shown to be linearly correlated[10] with $\frac{k_i}{k_{inact}}$ under the condition that $IC_{50}(0) \gg IC_{50}(t)$,

$$IC_{50}(t) = ln2\left(1 + \frac{[S]}{K_m}\right) / \left(t \cdot \frac{k_{inact}}{k_i}\right). \quad (10)$$

Therefore,

$$\frac{1}{IC_{50}(t)} = t \cdot \left(\frac{k'_{inact}}{k_i} + \frac{k''_{inact}}{k_i}\right) \Big/ \left[ln2\left(1 + \frac{[S]}{K_m}\right)\right], \quad (11)$$

Assuming the covalent reaction rates (i.e. k'_{inact} and k''_{inact}) are significantly slower than dissociation rate k_4, we arrive at the reciprocal relationship of IC_{50} for dual warhead molecular bident with two single warhead irreversible counterpart,

$$\frac{1}{IC_{50}(t)} \approx \frac{1}{IC'_{50}(t)} + \frac{1}{IC''_{50}(t)}, \quad (12)$$

where IC'_{50} and IC''_{50} represent IC_{50} of corresponding single warhead compounds with front and back warheads, respectively. With the above equation, IC_{50} of ZNL-8162 was estimated from ZNL-8163 (437 nM) and ZNL-8165 (313 nM) to be ~182nM.

Data processing of crystal structures

Cocrystal structures were retrieved from PDB database (www.rcsb.org). Each cocrystal structure was processed by PLIP[11] to detect ligands and calculate ligand protein interactions. Only small molecules were considered for subsequent distance calculations between ligand and cysteine residues. In addition to definition from PLIP, molecular weight filter of 150 – 1000 was used identify small molecules. The distance is defined between the closest distance between heavy atoms in ligand and corresponding cysteine residues. The distribution (Figure. 3A) was obtained by binning the distances observed in EGFR cocrystal pdb files with bin size of 0.5 Å. Multiple chains were considered separately, and only interactions among the same chain were used for searching potential cysteine pairs. Solvent accessible surface area (SASA) for cysteines was calculated using PYMOL (https://pymol.org) with default parameter values and binned with step size of 2 $Å^2$ (Figure. S2A). Accessible cysteines were identified if their SASA was larger than 10 $Å^2$ and not involved in disulfide bonds.

Molecular modeling

Covalent docking (CovDock in Schrodinger software suite) was used to predict binding modes and scores of designed bident and mono-valent molecules. Thorough mode was used for docking and top 5 poses for each molecule were inspected visually to identify the most likely binding conformation based on the known core scaffold binding mode. Hinge hydrogen bonds of the core scaffolds were used as constraints in docking calculations. Since CovDock does not allow multiple reactive residues, only one cysteine was specified for covalent bond formation during docking of molecular bidents. While CovDock did not provide final binding modes with dual covalent bonds for bident molecules, a proper binding mode required the other warhead in close proximity with its intended reactive cysteine. To obtain the final predicted binding mode in Figure 2B, the pre-reaction cysteine and warhead were manually linked with proper chemical bond, and the complex was optimized using receptor-ligand complex optimization protocol in Schrodinger suite with default settings. The final optimized complex was compared against the original binding mode to ensure minimal changes to the ligand and receptor residue conformations. To predict and compare binding modes for analogs of a same scaffold (Figure S3), covalent docking to single cysteine (Cys775) was performed, and the scores were chosen from poses that most resembled the parent molecule in the crystal structure (5FED).

Determining sites of probe modification sites by MS

EGFR protein was treated with indicated probe for 2 hours at 30 °C in 50 mM HEPES buffer 100 mM NaCl pH 7.5. Protein was reduced (10 mM dithiothreitol, 37 °C, 30 minutes), alkylated (22.5 mM iodoacetamide, 30 minutes, room temperature, protected from light), and digested overnight with trypsin (1:20 trypsin:EGFR; PROMEGA, Madison, WI) at 37 °C. Peptides were desalted by C18 and analyzed by nanoLC-MS using a Waters NanoAcquity UPLC (Waters, Milford, MA) interfaced to a Orbitrap QExactive HF mass spectrometer (ThermoFisher Scientific, San Jose, CA). Peptides were trapped on a self-packed pre-column (150 µm I.D. packed with 5 cm 7 µm SYMMETRY, Waters), resolved on an analytical column (30 µm I.D. packed with 50 cm of 5 µm Monitor, Orochem)[12] and gradient eluted (1-50% B in 60 minutes; A=0.1% formic acid, B=0.1% formic acid in acetonitrile) to the mass spectrometer (ESI spray voltage = 5 kV). The mass spectrometer was operated in data dependent mode such that the 10 most abundant ions in each

MS scan (*m/z* 300-2000, resolution=120k, target=1E6) were subjected to MS/MS (resolution=15k, AGC target=1E5, max fill time=100 ms, isolation width=1.4, fixed first mass=110, normalized collision energy=28, charge states 2-7). Dynamic exclusion was enabled with a repeat count of 1 and an exclusion duration of 30 seconds. Modified peptides were identified using Mascot version 2.6.2 searching against a custom database of lab proteins that included EGFR and MKK7 sequences (data file S4). Search parameters included 10 ppm and 25 mmu precurson and product ion tolerances, respectively as well as variable oxidation of methionine, fixed carbamidomethylation of cysteine, and variable probe modification (cysteine). Dual warhead targets were identified by calculating *m/z* values for expected cross-linked peptides using the PepCalc tool in mzStudio[13]. This tool was then used to make extracted ion chromatograms (XICs) for expected charge states of the cross-linked peptides (typically +4-+7). We then examined MS/MS spectra within XIC elution profiles that were identified with the "Find Precursors" function of PepCalc. We identified the cross-linked peptides by mapping y and b ions onto MS spectra (overlay button in PepCalc toolbar) and looking for high quality matches. Spectra were exported as svg from mzStudio, further annotated in Adobe Illustrator, and appear as Figures 2C and 3C.

Determining labeling stoichiometry by nanoLC-MS

Proteins were labeled, digested, and peptides desalted as described above. Peptides were then analyzed by nanoLC-MS using a NanoElute UPLC interfaced to a tims-TOF Pro 2 mass spectrometer (Bruker, Billerica, MA). Peptides were resolved on a 10 cm PepSep column and eluted (2-25% B in 6 minutes, 25-37% B in 6 minutes, 37-95% B in 6 minutes; A=0.1% formic acid, B=0.1% formic acid in acetonitrile; flow rate 400 nL/min) to the mass spectrometer (1.5kV, captive spray needle). The mass spectrometer performed cycles of PASEF with up to 10 ramps of 100 ms (1/k0 0.6-1.6; m/z 100-1600). Method parameters specified a target intensity of 14.5k and an intensity threshold of 1750. Active exclusion was enabled with an exclusion duration of 0.4 minutes.

Peak areas of Cys775 and Cys797 containing peptides, as well as non-cysteine containing normalization peptides were derived from extracted ion chromatograms generated with the Multiplierz toolkit[14]. (Note the current version on Github supports access to timsTOF data files: https://github.com/BlaisProteomics/multiplierz/tree/master). Labeling is assessed as reduction in

signal of cysteine containing peptides vs. DMSO control according to: 1-[(normalized peak area target Cys sequence - treated)/(normalized peak area target Cys sequence – DMSO)] X 100% +/- s.e.m. At least 2 replicates were performed for each condition.

Chemistry

General Methods.

Starting materials, reagents, and solvents were purchased from commercial suppliers and were used without further purification unless otherwise noted. All reactions were monitored using a Waters Acquity UPLC/MS system (Waters PDA eλ Detector, QDa Detector, Sample manager - FL, Binary Solvent Manager) using Acquity UPLC® BEH C18 column (2.1 x 50 mm, 1.7 µm particle size): solvent gradient = 85% A at 0 min, 1% A at 1.7 min; solvent A = 0.1% formic acid in Water; solvent B = 0.1% formic acid in Acetonitrile; flow rate : 0.6 mL/min. Reaction products were purified by flash column chromatography using CombiFlash®Rf with Teledyne Isco RediSep® normal-phase silica flash columns (4 g, 12 g, 24 g, 40 g or 80 g) and Waters HPLC system using SunFireTM Prep C18 column (19 x 100 mm, 5 µm particle size): solvent gradient = 80% A at 0 min, 10% A at 55 min; solvent A = 0.035% TFA in Water; solvent B = 0.035% TFA in MeOH; flow rate : 40 mL/min. ^{1}H NMR spectra were recorded on 400 and 500 MHz Bruker Avance III spectrometers. Chemical shifts are reported in parts per million (ppm, δ) downfield from tetramethylsilane (TMS). Coupling constants (J) are reported in Hz. Spin multiplicities are described as br (broad), s (singlet), d (doublet), t (triplet), q (quartet), m (multiplet).

Abbreviations Used

Ac_2O, acetic anhydride; DIEA, N,N-diisopropylethylamine; MeOH, methanol; DCM, dichloromethane; DMSO, dimethyl sulfoxide; DMF, dimethylformamide; EtOAc, ethyl acetate; HATU, hexafluorophosphate azabenzotriazole tetramethyl uronium; HCl, hydrogen chloride; LiOH, lithium hydroxide; Na_2SO_4, sodium sulfate; $NaHCO_3$, sodium bicarbonate; TEA, triethylamine; TFA, trifluoroacetic acid, THF, tetrahydrofuran.

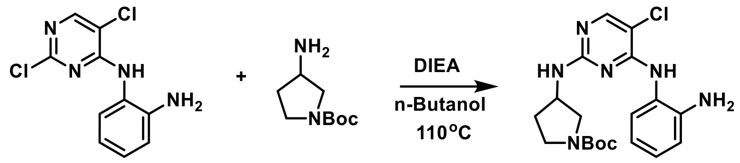

1 2 3

Synthesis of *tert*-butyl 3-((4-((2-aminophenyl)amino)-5-chloropyrimidin-2-yl)amino)pyrrolidine-1-carboxylate (3)

To a solution of N^1-(2,5-dichloropyrimidin-4-yl)benzene-1,2-diamine (200 mg, 1 eq, 784 μmol) in n-Butanol (10 mL) was added tert-butyl 3-aminopyrrolidine-1-carboxylate (292 mg, 2 eq, 1.57 mmol) and DIEA (683 μL, 5 eq, 3.92 mmol), the mixture was stirred at 110 °C for 2 hours. The reaction mixture was concentrated under reduced pressure to give a residue. Then the residue was purified by silica gel chromatography (10% MeOH in DCM) to give *tert*-butyl 3-((4-((2-aminophenyl)amino)-5-chloropyrimidin-2-yl)amino)pyrrolidine-1-carboxylate (300 mg, 94.5% yield). LC/MS (ESI) m/z calculated $[M+H]^+$ 405.90, found 405.43.

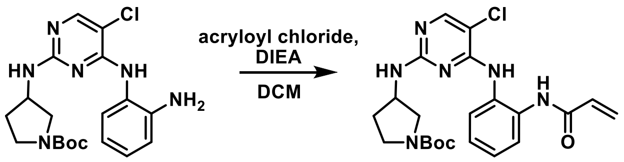

3 4

Synthesis of *tert*-butyl 3-((4-((2-acrylamidophenyl)amino)-5-chloropyrimidin-2-yl)amino)pyrrolidine-1-carboxylate (4)

To a solution of *tert*-butyl 3-((4-((2-aminophenyl)amino)-5-chloropyrimidin-2-yl)amino)pyrrolidine-1-carboxylate (150 mg, 1 eq, 370 μmol) and DIEA (195 μL, 3 eq, 1.11 mmol), in DCM (4 mL), was added a solution of acryloyl chloride (33.5 mg, 1 eq, 370 μmol) in 0.5 mL DCM within 10 min at 0 °C and the mixture was stirred for another 30 min. Evaporation of the reaction mixture to give the oil-like residue. Then the residue was purified by silica gel chromatography (10% MeOH in DCM) to give *tert*-butyl 3-((4-((2-acrylamidophenyl)amino)-5-chloropyrimidin-2-yl)amino)pyrrolidine-1-carboxylate **4** (110 mg, 65% yield). LC/MS (ESI) m/z calculated $[M+H]^+$ 459.95, found 459.64.

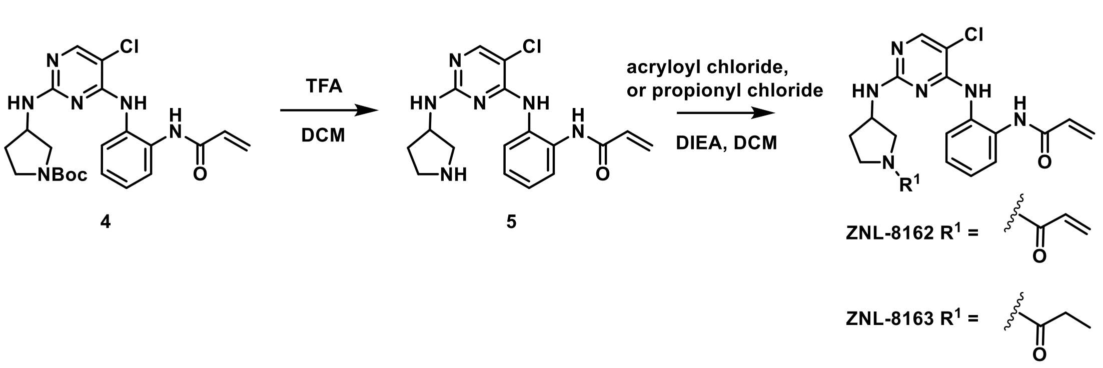

Synthesis of *N*-(2-((2-((1-acryloylpyrrolidin-3-yl)amino)-5-chloropyrimidin-4-yl)amino)phenyl)acrylamide (ZNL-8162)

A mixture of *tert*-butyl 3-((4-((2-acrylamidophenyl)amino)-5-chloropyrimidin-2-yl)amino)pyrrolidine-1-carboxylate (110 mg, 140 µmol, 1.0 eq) in DCM (4mL) and TFA (1 mL), and then the mixture was stirred at 25°C for 1 hr. Evaporation of the reaction mixture to give *N*-(2-((5-chloro-2-(pyrrolidin-3-ylamino)pyrimidin-4-yl)amino)phenyl)acrylamide **5** (90 mg) as yellow oil. LC/MS (ESI) m/z calculated $[M+H]^+$ 359.13, found 359.24.

To a solution of *N*-(2-((5-chloro-2-(pyrrolidin-3-ylamino)pyrimidin-4-yl)amino)phenyl)acrylamide **5** (45 mg, 1 eq, 130 µmol) and DIEA (66 µL, 3 eq, 380 µmol) in DCM (2 mL), was added a solution of acryloyl chloride (11 mg, 1 eq, 130 µmol) in 0.5 mL DCM within 10 min at 0 °C and the mixture was stirred for another 30 min. The reaction mixture was purified by prep-HPLC (mobile phase: [H_2O (0.035% TFA)/MeOH (0.035% TFA)]; gradient:80% - 10% over 60 min) to give *N*-(2-((2-((1-acryloylpyrrolidin-3-yl)amino)-5-chloropyrimidin-4-yl)amino)phenyl)acrylamide **ZNL-8162** (28 mg, 54% yield). LC/MS (ESI) m/z calculated $[M+H]^+$ 413.88, found 413.25.

^{1}H NMR (500 MHz, DMSO) δ 10.20 (s, 1H), 8.97 (s, 1H), 8.11 – 8.07 (m, 1H), 7.79 – 7.71 (m, 1H), 7.45 (dd, *J* = 7.8, 1.7 Hz, 1H), 7.32 – 7.20 (m, 2H), 6.64 – 6.39 (m, 2H), 6.33 (dd, *J* = 16.9, 2.0 Hz, 1H), 6.12 (ddd, *J* = 16.8, 5.9, 2.4 Hz, 1H), 5.82 (dd, *J* = 10.1, 1.9 Hz, 1H), 5.66 (ddd, *J* = 10.6, 8.1, 2.4 Hz, 1H), 3.74 – 3.24 (m, 4H), 2.15 – 1.78 (m, 2H), 1.26 – 1.23 (m, 1H).

Synthesis of *N*-(2-((5-chloro-2-((1-propionylpyrrolidin-3-yl)amino)pyrimidin-4-yl)amino)phenyl)acrylamide (ZNL-8163)

To a solution of *N*-(2-((5-chloro-2-(pyrrolidin-3-ylamino)pyrimidin-4-yl)amino)phenyl)acrylamide **5** (45 mg, 1 eq, 130 µmol) and DIEA(66 µL, 3 eq, 380 µmol) in DCM

(2 mL), was added a solution of propionyl chloride (12 mg, 1 eq, 130 μmol) in 0.5 mL DCM within 10 min at 0 °C and the mixture was stirred for another 30 min. The reaction mixture was purified by prep-HPLC (mobile phase: [H_2O (0.035% TFA)/MeOH (0.035% TFA)]; gradient:80% - 10% over 60 min) to give *N*-(2-((5-chloro-2-((1-propionylpyrrolidin-3-yl)amino)pyrimidin-4-yl)amino)phenyl)acrylamide **ZNL-8163** (35 mg, 67% yield). LC/MS (ESI) m/z calculated $[M+H]^+$ 415.89, found 415.30.

^{1}H NMR (500 MHz, DMSO) δ 10.20 (d, *J* = 24.4 Hz, 1H), 9.34 (s, 1H), 8.19 – 8.15 (m, 1H), 7.71 (ddd, *J* = 11.6, 8.6, 3.9 Hz, 1H), 7.56 – 7.47 (m, 1H), 7.28 (ddq, *J* = 8.7, 6.7, 2.7 Hz, 2H), 6.53 (ddd, *J* = 17.0, 10.2, 4.0 Hz, 1H), 6.33 (dt, *J* = 17.0, 2.0 Hz, 1H), 5.82 (dt, *J* = 10.2, 1.7 Hz, 1H), 4.03 (s, 1H), 3.58 – 3.17 (m, 4H), 2.51 (p, *J* = 1.9 Hz, 2H), 2.21 (q, *J* = 7.4 Hz, 1H), 2.17 – 1.77 (m, 2H), 0.96 (dt, *J* = 8.6, 7.4 Hz, 3H).

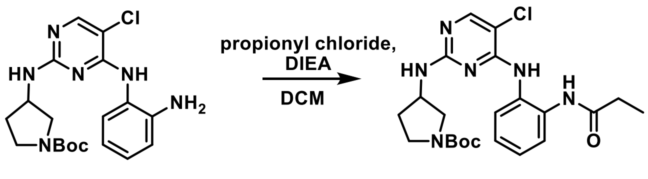

3 6

Synthesis of *tert*-butyl 3-((5-chloro-4-((2-propionamidophenyl)amino)pyrimidin-2-yl)amino)pyrrolidine-1-carboxylate (6)

To a solution of *tert*-butyl 3-((4-((2-aminophenyl)amino)-5-chloropyrimidin-2-yl)amino)pyrrolidine-1-carboxylate (150 mg, 1 eq, 370 μmol) and DIEA (195 μL, 3 eq, 1.11 mmol), in DCM (4 mL), was added a solution of propionyl chloride (34mg, 1 eq, 370 μmol) in 0.5 mL DCM within 10 min at 0 °C and the mixture was stirred for another 30 min. The reaction mixture was concentrated under reduced pressure to give the residue. Then the residue was purified by silica gel chromatography(10% MeOH in DCM) to give *tert*-butyl 3-((5-chloro-4-((2-propionamidophenyl)amino)pyrimidin-2-yl)amino)pyrrolidine-1-carboxylate **6** (135 mg, 79% yield). LC/MS (ESI) m/z calculated $[M+H]^+$ 461.96, found 461.19.

^{1}H NMR (500 MHz, DMSO) δ 10.18 (s, 1H), 9.34 (s, 1H), 8.29 (s, 1H), 8.19 – 8.15 (m, 1H), 7.71 (ddd, *J* = 11.6, 8.6, 3.9 Hz, 1H), 7.56 – 7.47 (m, 1H), 7.28 (dt, *J* = 8.7, 2.7 Hz, 2H), 6.53 (ddd, *J* = 17.0, 10.1, 4.0 Hz, 1H), 6.33 (dt, *J* = 17.0, 2.0 Hz, 1H), 5.82 (dt, *J* = 10.2, 1.7 Hz, 1H), 4.03 (s, 1H), 3.55 – 3.19 (m, 4H), 2.21 (q, *J* = 7.4 Hz, 1H), 2.14 (q, *J* = 7.7 Hz, 1H), 2.02 – 1.77 (m, 2H), 0.96 (dt, *J* = 8.6, 7.4 Hz, 3H).

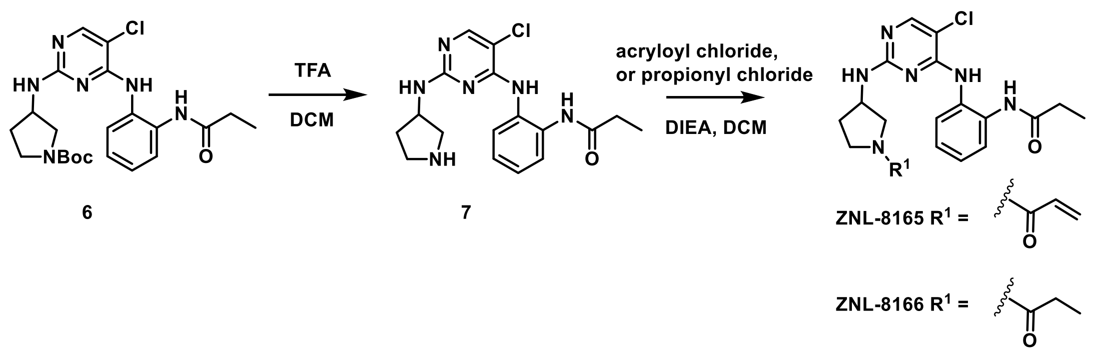

Synthesis of *N*-(2-((2-((1-acryloylpyrrolidin-3-yl)amino)-5-chloropyrimidin-4-yl)amino)phenyl)propionamide (ZNL-8165)

A mixture of *tert*-butyl 3-((5-chloro-4-((2-propionamidophenyl)amino)pyrimidin-2-yl)amino)pyrrolidine-1-carboxylate (160 mg, 395 µmol, 1.0 eq) in DCM (5mL) and TFA (1 mL), and then the mixture was stirred at 25°C for 1 hr. The reaction mixture was concentrated under reduced pressure to give *N*-(2-((5-chloro-2-(pyrrolidin-3-ylamino)pyrimidin-4-yl)amino)phenyl)acrylamide **7** as yellow oil without further purification.

To a solution of *N*-(2-((5-chloro-2-(pyrrolidin-3-ylamino)pyrimidin-4-yl)amino)phenyl)acrylamide **7** (52 mg, 1 eq, 140 µmol) and DIEA(76 µL, 3 eq, 430 µmol) in DCM (2 mL), was added a solution of acryloyl chloride (13 mg, 1 eq, 140 µmol) in 0.5 mL DCM within 10 min at 0 °C and the mixture was stirred for another 30 min. The reaction mixture was purified by prep-HPLC (mobile phase: [H_2O (0.035% TFA)/MeOH (0.035% TFA)]; gradient:80% - 10% over 60 min) to give *N*-(2-((2-((1-acryloylpyrrolidin-3-yl)amino)-5-chloropyrimidin-4-yl)amino)phenyl)propionamide **ZNL-8165** (30 mg, 50% yield). LC/MS (ESI) m/z calculated $[M+H]^+$ 415.89, found 415.25.

^{1}H NMR (500 MHz, DMSO) δ 9.99 (d, *J* = 22.4 Hz, 1H), 9.47 (s, 1H), 8.53 (s, 1H), 8.23 (s, 1H), 7.71 (td, *J* = 7.7, 4.3 Hz, 1H), 7.44 – 7.38 (m, 1H), 7.27 (qd, *J* = 5.8, 4.0 Hz, 2H), 6.51 (ddd, *J* = 62.8, 16.7, 10.3 Hz, 1H), 6.13 (ddd, *J* = 16.8, 5.1, 2.4 Hz, 1H), 5.67 (ddd, *J* = 10.4, 4.5, 2.4 Hz, 1H), 4.07 (s, 1H), 3.69 – 3.53 (m, 1H), 3.52 – 3.32 (m, 2H), 2.39 (qd, *J* = 7.6, 2.6 Hz, 2H), 2.18 – 1.79 (m, 2H), 1.11 (td, *J* = 7.6, 2.5 Hz, 3H).

Synthesis of *N*-(2-((5-chloro-2-((1-propionylpyrrolidin-3-yl)amino)pyrimidin-4-yl)amino)phenyl)propionamide (ZNL-8166)

To a solution of *N*-(2-((5-chloro-2-(pyrrolidin-3-ylamino)pyrimidin-4-yl)amino)phenyl)acrylamide **7** (50 mg, 1 eq, 140 μmol) and DIEA(73 μL, 3 eq, 430 μmol) in DCM (2 mL), was added a solution of propionyl chloride (13 mg, 1 eq, 140 μmol) in 0.5 mL DCM within 10 min at 0 °C The reaction mixture was purified by prep-HPLC (mobile phase: [H_2O (0.035% TFA)/MeOH (0.035% TFA)]; gradient:80% - 10% over 60 min) to give *N*-(2-((5-chloro-2-((1-propionylpyrrolidin-3-yl)amino)pyrimidin-4-yl)amino)phenyl)propionamide **ZNL-8166** (36 mg, 62% yield). LC/MS (ESI) m/z calculated $[M+H]^+$ 417.91, found 417.30.

^1H NMR (500 MHz, DMSO) δ 10.01 (s, 1H), 9.71 (s, 1H), 8.93 (s, 1H), 8.32 (s, 1H), 7.71 – 7.63 (m, 1H), 7.50 – 7.41 (m, 1H), 7.28 (ddd, *J* = 9.3, 7.3, 5.6 Hz, 2H), 4.05 (s, 1H), 3.60 – 3.21 (m, 3H), 2.39 (qd, *J* = 7.5, 2.3 Hz, 2H), 2.28 – 1.76 (m, 4H), 1.11 (td, *J* = 7.6, 2.6 Hz, 3H), 0.96 (q, *J* = 7.2 Hz, 3H).

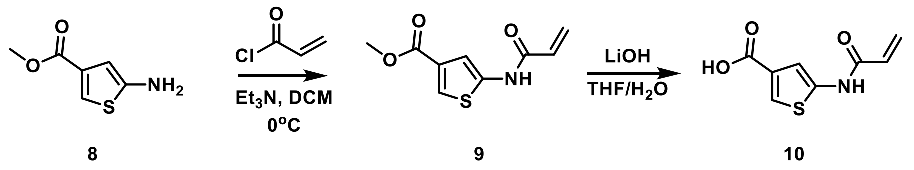

Synthesis of 5-acrylamidothiophene-3-carboxylic acid (10)

To a solution of compound methyl 5-aminothiophene-3-carboxylate **8** (1.5 g, 1 eq, 9.54 mmol) and Et_3N (1.93 g, 2 eq, 19.09 mmol) in dichloromethane (20 mL) was added dropwise of acryloyl chloride (1.3 g, 1.5 eq, 14.3 mmol) at 0 °C. The reaction was stirred for another1 hour. The reaction was quenched by water (20 mL) and extracted with EtOAc (2 x 50 mL). The organic layer was dried over Na_2SO_4 and filtered. The filtrate was concentrated and the crude was purified by silica gel chromatography (30% EtOAc in hexane) to afford methyl 5-acrylamidothiophene-3-carboxylate **9** (700 mg, 35%) as a yellow solid). LC/MS (ESI) m/z calculated $[M+H]^+$ 212.03, found 212.08.

To a solution of methyl 5-acrylamidothiophene-3-carboxylate **9** (150 mg, 1 eq, 0.71 mmol) in THF (8 mL) was added a solution of $LiOH{\cdot}H_2O$ (89 mg, 3 eq, 2.13 mmol) in H_2O (2 mL). The solution was stirred at room temperature for 3 hours. Then the reaction solution was acidified to pH 6~7 using 1M HCl and extracted by EtOAc (2 x 20 mL). The organic layer was dried over Na_2SO_4 and filtered. The filtrate was concentrated to afford 5-acrylamidothiophene-3-carboxylic acid **10** (135 mg, 96%) as a yellow solid without further purification. LC/MS (ESI) m/z calculated $[M+H]^+$ 198.01, found 198.04.

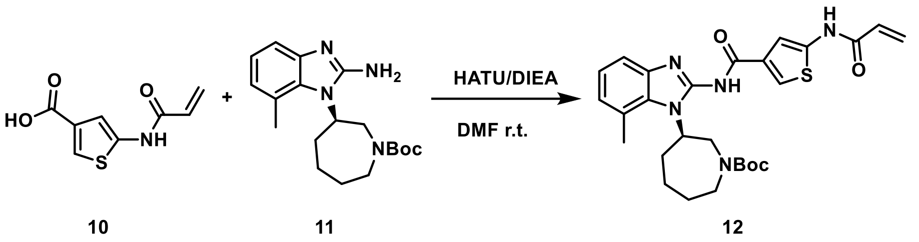

Synthesis of *tert*-butyl (*R*)-3-(2-(5-acrylamidothiophene-3-carboxamido)-7-methyl-1*H*-benzo[*d*]imidazol-1-yl)azepane-1-carboxylate (12)

tert-butyl (*R*)-3-(2-amino-7-methyl-1*H*-benzo[*d*]imidazol-1-yl)azepane-1-carboxylate **11** is prepared followed the procedure as previously described (*31*). A solution of *tert*-butyl (*R*)-3-(2-amino-7-methyl-1*H*-benzo[*d*]imidazol-1-yl)azepane-1-carboxylate (180 mg, 1 eq, 0.52 mmol), compound 5-acrylamidothiophene-3-carboxylic acid (112 mg, 1.1 eq, 0.57 mmol), HATU (237 mg, 1.2 eq, 0.62 mmol) and DIEA (201 mg, 3 eq, 1.56 mmol) in DMF (5 mL) was stirred at room temperature for 8 hours. The reaction was diluted with EtOAc (50 mL) and washed water and brine. The organic layer was dried over Na_2SO_4, filtered. The filtrate was concentrated and the residue was purified by silica gel chromatography (10% MeOH in DCM) to afford compound *tert*-butyl (*R*)-3-(2-(5-acrylamidothiophene-3-carboxamido)-7-methyl-1*H*-benzo[*d*]imidazol-1-yl)azepane-1-carboxylate (150 mg, 55%). LC/MS (ESI) m/z calculated $[M+H]^+$ 524.65, found 524.44.

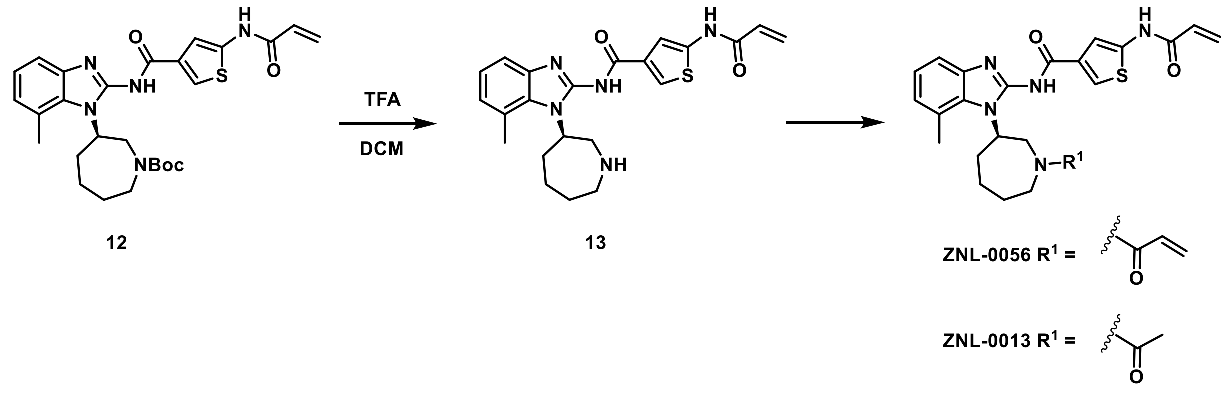

Synthesis of (*R*)-5-acrylamido-*N*-(1-(1-acryloylazepan-3-yl)-7-methyl-1*H*-benzo[*d*]imidazol-2-yl)thiophene-3-carboxamide (ZNL-0056)

A solution of *tert*-butyl (*R*)-3-(2-(5-acrylamidothiophene-3-carboxamido)-7-methyl-1*H*-benzo[*d*]imidazol-1-yl)azepane-1-carboxylate (150 mg, 0.29 mmol) in DCM (4 mL) and TFA (1 mL) was stirred at room temperature for 2 hours. The reaction mixture was concentrated under

reduced pressure to give (*R*)-5-acrylamido-*N*-(1-(azepan-3-yl)-7-methyl-1*H*-benzo[*d*]imidazol-2-yl)thiophene-3-carboxamide **13** as yellow oil without further purification.

To a solution of (*R*)-5-acrylamido-*N*-(1-(azepan-3-yl)-7-methyl-1*H*-benzo[*d*]imidazol-2-yl)thiophene-3-carboxamide **13** (100 mg, 1 eq, 0.24 mmol), TEA (48 mg, 2 eq, 0.47 mmol) in DCM (5 mL) was added acryloyl chloride (28 mg, 1.2 eq, 0.31 mmol) at 0°C. The solution was then stirred at room temperature for 30 min. The organic layer was concentrated and the residue was purified by prep-HPLC (mobile phase: [H_2O (0.035% TFA)/MeOH (0.035% TFA)]; gradient:80% - 10% over 60 min) to give (*R*)-5-acrylamido-*N*-(1-(1-acryloylazepan-3-yl)-7-methyl-1*H*-benzo[*d*]imidazol-2-yl)thiophene-3-carboxamide **ZNL-0056** (21.6 mg, 19%) as a white solid. LC/MS (ESI) m/z calculated $[M+H]^+$ 478.58, found 478.25.

^{1}H NMR (400 MHz, DMSO-d_6) δ 12.81 (s, 0.3H), 12.78 (s, 0.7H), 11.43 (s, 1H), 7.70 (s, 0.7H), 7.67 (s, 0.3H), 7.40 (d, J = 7.4 Hz, 1H), 7.24 (d, *J* = 1.6 Hz, 0.7H), 7.22 (d, *J* = 1.7 Hz, 0.3H), 7.11 (dd, *J* = 8.6, 7.0 Hz, 1H), 7.02 (s, 0.7H), 7.00 (s, 0.3H), 6.93 (dd, *J* = 16.6, 10.4 Hz, 0.3H), 6.86 (dd, *J* = 16.6, 10.4 Hz, 0.7H), 6.46 (d, *J* = 9.9 Hz, 0.3H), 6.42 (d, *J* = 10.0 Hz, 0.7H), 6.35 (d, *J* = 2.1 Hz, 0.7H), 6.31 (d, *J* = 2.1 Hz, 0.3H), 6.22 (ddd, *J* = 16.7, 7.8, 2.4 Hz, 1H), 5.85 (dd, *J* = 9.9, 2.1 Hz, 1H), 5.75 (dd, *J* = 10.4, 2.4 Hz, 0.7H), 5.68 (dd, *J* = 10.3, 2.5 Hz, 0.3H), 4.91 – 4.59 (m, 1H), 4.36 – 4.15 (m, 1H), 4.08 (dt, *J* = 14.3, 7.0 Hz, 0.3H), 3.96 (dt, *J* = 14.1, 6.9 Hz, 0.7H), 3.58 (dd, J = 13.8, 5.8 Hz, 1H), 2.82 – 2.63 (m, 4H), 2.07 – 1.89 (m, 4H), 1.43 – 1.28 (m, 1H).

Synthesis of (*R*)-*N*-(1-(1-acetylazepan-3-yl)-7-methyl-1*H*-benzo[*d*]imidazol-2-yl)-5-acrylamidothiophene-3-carboxamide (ZNL-0013)

To a solution of compound (*R*)-5-acrylamido-*N*-(1-(azepan-3-yl)-7-methyl-1*H*-benzo[*d*]imidazol-2-yl)thiophene-3-carboxamide **13** (50 mg, 1 eq, 0.12 mmol), TEA (24 mg, 2 eq, 0.24 mmol) in DCM (3 mL) was added Ac_2O (15 mg, 1.2 eq, 0.14 mmol). The reaction solution was stirred at room temperature for 30 min. The organic layer was concentrated and the residue was purified by prep-HPLC (mobile phase: [H_2O (0.035% TFA)/MeOH (0.035% TFA)]; gradient:80% - 10% over 60 min) to give (*R*)-*N*-(1-(1-acetylazepan-3-yl)-7-methyl-1*H*-benzo[*d*]imidazol-2-yl)-5-acrylamidothiophene-3-carboxamide **ZNL-0013** (25 mg, 46%) as a white solid. LC/MS (ESI) m/z calculated $[M+H]^+$ 466.57, found 466.20.

^{1}H NMR (500 MHz, DMSO) δ 12.76 (s, 1H), 11.45 (s, 0.3H), 11.43 (s, 0.7H), 7.68 (d, *J* = 1.5 Hz, 0.7H), 7.66 (d, *J* = 1.6 Hz, 0.3H), 7.42 (dd, *J* = 11.0, 7.8 Hz, 1H), 7.22 (d, *J* = 1.6 Hz, 0.7H), 7.20

(d, J = 1.6 Hz, 0.3H), 7.10 (q, J = 7.9 Hz, 1H), 7.02 (d, J = 7.5 Hz, 0.3H), 6.99 (d, J = 7.5 Hz, 0.7H), 6.42 (dd, J = 17.0, 10.0 Hz, 1H), 6.32 (dd, J = 17.0, 2.0 Hz, 1H), 5.84 (dd, J = 10.0, 2.0 Hz, 1H), 4.79 (dtt, J = 13.5, 9.9, 5.0 Hz, 1H), 4.24 (dd, J = 13.1, 10.2 Hz, 1H), 4.11 (dd, J = 13.1, 3.1 Hz, 1H), 3.72 (ddd, J = 14.7, 8.7, 6.4 Hz, 1H), 3.55 (ddd, J = 13.7, 6.4, 4.3 Hz, 1H), 2.74 – 2.63 (m, 4H), 2.08 (d, J = 20.1 Hz, 3H), 1.96 (td, J = 12.2, 5.1 Hz, 3H), 1.41 (dd, J = 15.3, 9.8 Hz, 1H).

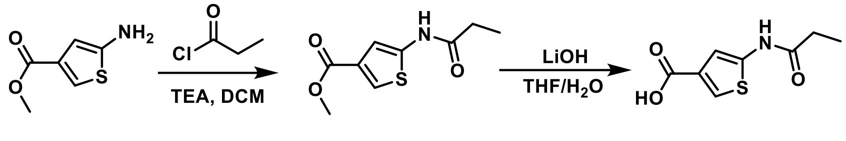

14 15 16

Synthesis of 5-propionamidothiophene-3-carboxylic acid (16)

To a solution of methyl 5-aminothiophene-3-carboxylate (1 g, 1 eq, 6.36 mmol) and TEA (1.93 g, 3 eq, 19.09 mmol) in DCM (20 mL) was added dropwise of propionyl chloride (706 mg, 1.2 eq, 7.63 mmol) at 0 °C, then the solution was stirred at 0 °C for 1 hour. The reaction was quenched by H_2O (20 mL) and extracted with EtOAc (2 x 30 mL). The organic layer was dried over Na_2SO_4 and filtered. The filtrate was concentrated and the crude was purified by silica gel chromatography (30% EtOAc in hexane) to afford methyl 5-propionamidothiophene-3-carboxylate (**15**) (1.1 g, 81%) as a yellow solid. LC/MS (ESI) m/z calculated [M+H]+ 214.05, found 214.21.

^{1}H NMR (400 MHz, DMSO-d_6) δ 11.23 (s, 1H), 7.72 (d, J = 1.8 Hz, 1H), 6.91 (t, J = 1.4 Hz, 1H), 3.76 (d, J = 1.2 Hz, 3H), 2.36 (qd, J = 7.6, 1.2 Hz, 2H), 1.09 (td, J = 7.5, 1.1 Hz, 3H).

To a solution of methyl 5-propionamidothiophene-3-carboxylate **15** (450 mg, 1 eq, 2.11 mmol) in THF (25 mL) was added a solution of LiOH·H_2O (270 mg, 3 eq, 6.4 mmol) in H_2O (6 mL). The reaction solution was stirred at room temperature for 16 hours. The solution was acidified to pH 6~7 using 1M HCl and then extracted with EtOAc (2 x 40 mL). The organic layer was dried over Na_2SO_4 and filtered. The filtrate was concentrated to afford 5-acrylamidothiophene-3-carboxylic acid **16** (380 mg, 91%) as a yellow solid without further purification. LC/MS (ESI) m/z calculated $[M+H]^+$ 200.03, found 200.21.

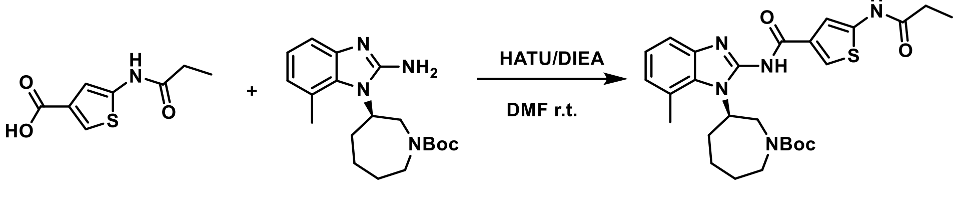

16 11 17

Synthesis of *tert*-butyl (*R*)-3-(7-methyl-2-(5-propionamidothiophene-3-carboxamido)-1*H*-benzo[*d*]imidazol-1-yl)azepane-1-carboxylate (17)

A solution of *tert*-butyl (*R*)-3-(2-amino-7-methyl-1*H*-benzo[*d*]imidazol-1-yl)azepane-1-carboxylate (360 mg, 1 eq, 1.04 mmol), 5-acrylamidothiophene-3-carboxylic acid **16** (225 mg, 1.1 eq, 1.14 mmol), HATU (475 mg, 1.2 eq, 1.24 mmol) and DIEA (400 mg, 3 eq, 3.12 mmol) in DMF (10 mL) was stirred at room temperature for 8 hours. The reaction was diluted with EtOAc (50 mL) and washed by water and brine. The organic layer was dried over Na_2SO_4 and filtered. The filtrate was concentrated and the residue was purified by silica gel chromatography (10% MeOH in DCM) to afford (*R*)-3-(7-methyl-2-(5-propionamidothiophene-3-carboxamido)-1*H*-benzo[*d*]imidazol-1-yl)azepane-1-carboxylate (340 mg, 62%). LC/MS (ESI) m/z calculated $[M+H]^+$ 526.67, found 526.25.

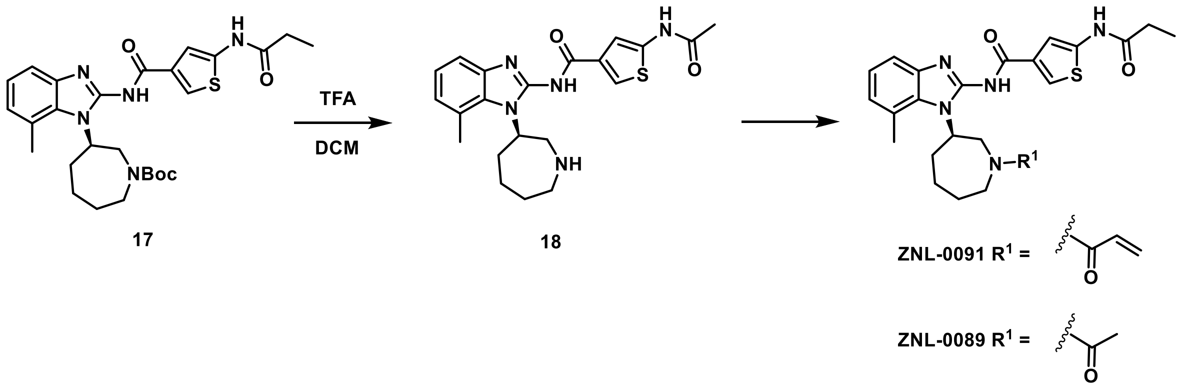

Synthesis of (*R*)-*N*-(1-(1-acryloylazepan-3-yl)-7-methyl-1*H*-benzo[*d*]imidazol-2-yl)-5-propionamidothiophene-3-carboxamide (ZNL-0091)

A solution of (*R*)-3-(7-methyl-2-(5-propionamidothiophene-3-carboxamido)-1*H*-benzo[*d*]imidazol-1-yl)azepane-1-carboxylate **17** (340 mg, 0.65 mmol) in DCM (10 mL) and TFA (2 mL) was stirred at room temperature for 2 hours. The reaction mixture was concentrated under reduced pressure to give (*R*)-*N*-(1-(azepan-3-yl)-7-methyl-1*H*-benzo[*d*]imidazol-2-yl)-5-propionamidothiophene-3-carboxamide **18** as yellow oil without further purifcation. LC/MS (ESI) m/z calculated [M+H]+ 426.55, found 426.25.

To a solution of (*R*)-*N*-(1-(azepan-3-yl)-7-methyl-1*H*-benzo[*d*]imidazol-2-yl)-5-propionamidothiophene-3-carboxamide **18** (120 mg, 1 eq, 0.28 mmol) and TEA (57 mg, 2 eq, 0.56 mmol) in DCM (5 mL) was added acryloyl chloride (30.6 mg, 1.2 eq, 0.34 mmol) at 0 ^{o}C. The

reaction was stirred at 0°C for 30 min. The organic layer was concentrated and the residue was purified by prep-HPLC (mobile phase: [H_2O (0.035% TFA)/MeOH (0.035% TFA)]; gradient:80% - 10% over 60 min) to give (*R*)-*N*-(1-(1-acryloylazepan-3-yl)-7-methyl-1*H*-benzo[*d*]imidazol-2-yl)-5-propionamidothiophene-3-carboxamide **ZNL-0091** (23 mg, 17%) as a white solid. LC/MS (ESI) m/z calculated $[M+H]^+$ 480.20, found 480.25.

^{1}H NMR (500 MHz, DMSO) δ 12.76 (s, 1H), 11.09 (s, 1H), 7.60 (d, *J* = 15.3 Hz, 1H), 7.41 (t, *J* = 7.7 Hz, 1H), 7.14 – 6.98 (m, 2H), 6.88 (ddd, *J* = 37.0, 16.5, 10.4 Hz, 1H), 6.21 (ddd, *J* = 16.8, 10.4, 2.4 Hz, 1H), 5.71 (ddd, *J* = 33.9, 10.3, 2.4 Hz, 1H), 4.97 – 3.80 (m, 5H), 3.64 – 3.50 (m, 1H), 2.89 – 2.53 (m, 4H), 2.38 (q, *J* = 7.5 Hz, 2H), 2.18 – 1.66 (m, 4H), 1.33 (d, *J* = 11.0 Hz, 1H), 1.12 (t, *J* = 7.5 Hz, 2H).

Synthesis of (*R*)-*N*-(1-(1-acetylazepan-3-yl)-7-methyl-1*H*-benzo[*d*]imidazol-2-yl)-5-propionamidothiophene-3-carboxamide (ZNL-0089)

To a solution of (*R*)-*N*-(1-(azepan-3-yl)-7-methyl-1*H*-benzo[*d*]imidazol-2-yl)-5-propionamidothiophene-3-carboxamide **18** (100 mg, 1 eq, 0.23 mmol) and TEA (48 mg, 2 eq, 0.47 mmol) in DCM (5 mL) was added acetic anhydride (29 mg, 1.1 eq, 0.28 mmol) at 0 °C. The reaction was stirred at room temperature for another 30 min. LCMS showed reaction completed. The reaction was stirred at 0°C for 30 min. The organic layer was concentrated and the residue was purified by prep-HPLC (mobile phase: [H_2O (0.035% TFA)/MeOH (0.035% TFA)]; gradient:80% - 10% over 60 min) to give (*R*)-*N*-(1-(1-acetylazepan-3-yl)-7-methyl-1*H*-benzo[*d*]imidazol-2-yl)-5-propionamidothiophene-3-carboxamide **ZNL-0089** (42 mg, 39%) as a white solid. LC/MS (ESI) m/z calculated $[M+H]^+$ 468.59, found 468.30.

^{1}H NMR (500 MHz, DMSO) δ 12.77 (d, *J* = 26.2 Hz, 1H), 11.10 (d, *J* = 10.6 Hz, 1H), 7.62 – 7.56 (m, 1H), 7.40 (d, *J* = 8.1 Hz, 1H), 7.14 – 7.05 (m, 2H), 7.00 (dd, *J* = 17.3, 7.5 Hz, 1H), 4.86 – 4.58 (m, 2H), 4.33 – 4.04 (m, 2H), 3.78 – 3.62 (m, 1H), 3.61 – 3.46 (m, 1H), 2.67 (d, *J* = 19.9 Hz, 4H), 2.38 (q, *J* = 7.5 Hz, 2H), 2.20 – 1.68 (m, 7H), 1.40 (t, *J* = 12.0 Hz, 1H), 1.12 (t, *J* = 7.6 Hz, 2H).

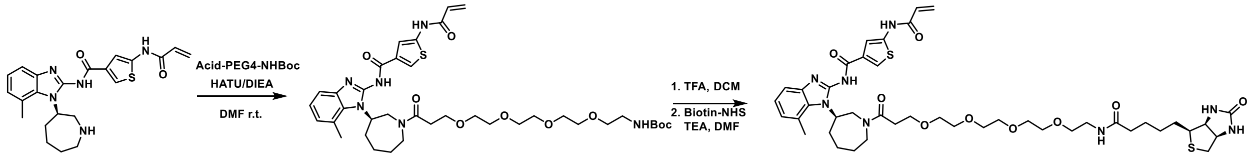

13 19 bio-ZNL-0013

Synthesis of 5-acrylamido-*N*-(7-methyl-1-((*R*)-1-(17-oxo-21-((3a*S*,4*S*,6a*R*)-2-oxohexahydro-1*H*-thieno[3,4-d]imidazol-4-yl)-4,7,10,13-tetraoxa-16-azahenicosanoyl)azepan-3-yl)-1*H*-benzo[*d*]imidazol-2-yl)thiophene-3-carboxamide (bio-ZNL-0013)

A solution of (*R*)-5-acrylamido-*N*-(1-(azepan-3-yl)-7-methyl-1*H*-benzo[*d*]imidazol-2-yl)thiophene-3-carboxamide (140 mg, 1 eq, 0.3 mmol), 2,2-dimethyl-4-oxo-3,8,11,14,17-pentaoxa-5-azaicosan-20-oic acid (acid-PEG4-NHBoc) (133 mg, 1.1 eq, 0.36 mmol), HATU (150 mg, 1.2 eq, 0.4 mmol) and DIEA (115 mg, 3 eq, 0.9 mmol) in DMF (5 mL) was stirred at room temperature for 8 hours. The reaction was diluted with EtOAc (50 mL) and washed water and brine. The organic layer was dried over Na_2SO_4, filtered. The filtrate was concentrated and the residue was purified by silica gel chromatography (10% MeOH in DCM) to afford *tert*-butyl (*R*)-(15-(3-(2-(5-acrylamidothiophene-3-carboxamido)-7-methyl-1*H*-benzo[*d*]imidazol-1-yl)azepan-1-yl)-15-oxo-3,6,9,12-tetraoxapentadecyl)carbamate (160 mg, 63%). LC/MS (ESI) m/z calculated $[M+H]^+$ 771.94, found 771.40.

To a solution of (*R*)-3-(7-methyl-2-(5-propionamidothiophene-3-carboxamido)-1*H*-benzo[*d*]imidazol-1-yl)azepane-1-carboxylate **17** (160 mg) in DCM (5 mL) was added TFA (1 mL) was stirred at room temperature for 2 hours. The reaction mixture was concentrated under reduced pressure to give (*R*)-5-acrylamido-*N*-(1-(1-(1-amino-3,6,9,12-tetraoxapentadecan-15-oyl)azepan-3-yl)-7-methyl-1*H*-benzo[*d*]imidazol-2-yl)thiophene-3-carboxamide without further purification. LC/MS (ESI) m/z calculated $[M+H]^+$ 671.83, found 671.42.

To a solution of (*R*)-5-acrylamido-*N*-(1-(1-(1-amino-3,6,9,12-tetraoxapentadecan-15-oyl)azepan-3-yl)-7-methyl-1*H*-benzo[*d*]imidazol-2-yl)thiophene-3-carboxamide (40 mg, 0.06 mmol) and Biotin NHS (22 mg, 0.06 mmol) in DMF (2 mL) at room temperature was added TEA (16 mg, 0.36 mmol) with stirring for 1 hour. The reaction mixture The organic layer was concentrated and the residue was purified by prep-HPLC (mobile phase: [H_2O (0.035% TFA)/MeOH (0.035% TFA)]; gradient:70% - 10% over 60 min) to give 5-acrylamido-N-(7-methyl-1-((R)-1-(17-oxo-21-((3aS,4S,6aR)-2-oxohexahydro-1H-thieno[3,4-d]imidazol-4-yl)-4,7,10,13-tetraoxa-16-azahenicosanoyl)azepan-3-yl)-1H-benzo[d]imidazol-2-yl)thiophene-3-carboxamide **bio-ZNL-0013** (7.2 mg, yield 14%) as white solid. LC/MS (ESI) m/z calculated [M+H]+ 897.39, found 897.20.

^{1}H NMR (400 MHz, DMSO-d_6) δ 12.75 (s, 1H), 11.46 (s, 1H), 7.80 (q, *J* = 4.4, 3.1 Hz, 1H), 7.66 (dd, *J* = 8.9, 1.5 Hz, 1H), 7.39 (d, *J* = 8.2 Hz, 1H), 7.20 (dd, *J* = 7.6, 1.6 Hz, 1H), 7.09 (q, *J* = 7.7,

7.1 Hz, 1H), 6.99 (t, J = 9.1 Hz, 1H), 6.48 – 6.25 (m, 4H), 5.83 (dd, J = 9.9, 2.1 Hz, 1H), 4.82 – 4.73 (m, 1H), 4.69 – 4.19 (m, 2H), 4.15 – 3.96 (m, 2H), 3.85 – 3.75 (m, 1H), 3.72 – 3.63 (m, 1H), 3.60 – 3.42 (m, 16H), 3.20 – 3.12 (m, 2H), 3.10 – 3.03 (m, 1H), 2.84 – 2.52 (m, 8H), 2.11 – 1.86 (m, 6H), 1.65 – 1.54 (m, 1H), 1.53 – 1.36 (m, 4H), 1.34 – 1.20 (m, 2H).

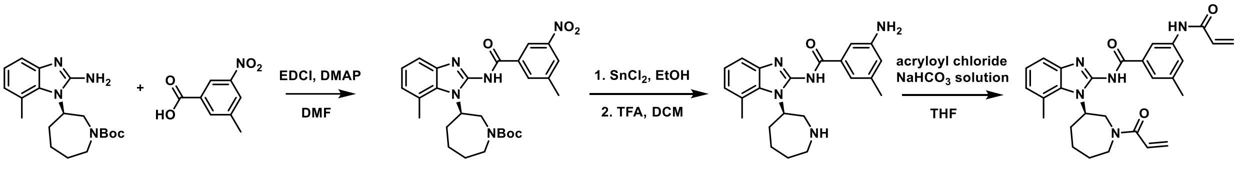

Synthesis of (*R*)-3-acrylamido-*N*-(1-(1-acryloylazepan-3-yl)-7-methyl-1*H*-benzo[*d*]imidazol-2-yl)-5-methylbenzamide (ZNL-0245)

A solution of *tert*-butyl (*R*)-3-(2-amino-7-methyl-1*H*-benzo[*d*]imidazol-1-yl)azepane-1-carboxylate (1.6 g, 1 eq, 4.6 mmol), 3-methyl-5-nitrobenzoic acid (1.0 g, 1.2 eq, 5.52 mmol), EDCI (1.76 g, 2 eq, 9.17 mmol) and DMAP (280 mg, 0.5 eq, 2.29 mmol) in 20 mL of DMF was stirred at room temperature for 2 hours. The reaction was poured into water (200 mL), extracted with EtOAc (50 mL x 3), washed with H_2O (40 mL) and brine (40 mL), dried with Na_2SO_4. The solvent was removed under reduced pressure then the residue was purified by silica gel chromatography (20% EtOAc in hexane) to afford *tert*-butyl (*R*)-3-(7-methyl-2-(3-methyl-5-nitrobenzamido)-1*H*-benzo[*d*]imidazol-1-yl)azepane-1-carboxylate **21** (1.8 g, 64.3 %). LC/MS (ESI) m/z calculated $[M+H]^+$ 508.59, found 508.28.

To a solution of *tert*-butyl (*R*)-3-(7-methyl-2-(3-methyl-5-nitrobenzamido)-1*H*-benzo[*d*]imidazol-1-yl)azepane-1-carboxylate **21** (300 mg, 0.6 mmol) in ethanol (15 mL) was added $SnCl_2.H_2O$ (660 mg, 3.0 mmol). The reaction was heated at 70°C for 2 hours. The reaction was quenched with saturated $NaHCO_3$ (20 mL) aqueous in water, extracted with EtOAc (20 mL x 3). The combined organic phase was dried over Na_2SO_4, concentrated under reduce pressure. The crude product was purified by silica gel chromatography (30% EtOAc in hexane) to afford *tert*-butyl (*R*)-3-(2-(3-amino-5-methylbenzamido)-7-methyl-1*H*-benzo[*d*]imidazol-1-yl)azepane-1-carboxylate (210 mg, 74.5%) as yellow solid. The solid was dissovled in DCM (3 mL), then TFA (1 mL) was added, the mixture was stirred at room temperature for 1 hour. The mixture was concentrated to give 120 mg crude (*R*)-3-amino-*N*-(1-(azepan-3-yl)-7-methyl-1*H*-

benzo[*d*]imidazol-2-yl)-5-methylbenzamide **22**. LC/MS (ESI) m/z calculated $[M+H]^+$ 378.22, found 378.26.

To a solution of (*R*)-3-amino-*N*-(1-(azepan-3-yl)-7-methyl-1*H*-benzo[*d*]imidazol-2-yl)-5-methylbenzamide **22** (120 mg, 0.32 mmol) in THF (10 mL)/saturated aqueous $NaHCO_3$ (2 mL) at 0°C was added dropwise acryloyl chloride (1 M in DCM, 636 µL). The reaction was warmed to room temperature and stirred for 15 min. The reaction was purified by prep-HPLC (mobile phase: [H_2O (0.035% TFA)/MeOH (0.035% TFA)]; gradient:80% - 10% over 60 min) directly to afford (*R*)-3-acrylamido-*N*-(1-(1-acryloylazepan-3-yl)-7-methyl-1*H*-benzo[*d*]imidazol-2-yl)-5-methylbenzamide **ZNL-0245** (40 mg, 26%) as white solid. LC/MS (ESI) m/z calculated $[M+H]^+$ 486.59, found 486.40.

^{1}H NMR (500 MHz, DMSO) δ 12.89 (s, 0.3H), 12.86 (s, 0.7H), 10.20 (d, *J* = 3.1 Hz, 1H), 8.49 (s, 0.7H), 8.47 (s, 0.3H), 7.78 (s, 0.7H), 7.76 (s, 0.3H), 7.53 (t, *J* = 1.9 Hz, 1H), 7.46 (t, *J* = 7.6 Hz, 1H), 7.11 (td, *J* = 7.7, 1.6 Hz, 1H), 7.01 (d, *J* = 7.4 Hz, 1H), 6.93 (dd, *J* = 16.5, 10.4 Hz, 0.3H), 6.85 (dd, *J* = 16.7, 10.4 Hz, 0.7H), 6.47 (ddd, *J* = 16.9, 10.0, 1.5 Hz, 1H), 6.32 – 6.17 (m, 2H), 5.82 – 5.64 (m, 2H), 4.95 – 4.82 (m, 0.7H), 4.72 – 4.63 (m, 0.3H), 4.42 (dd, *J* = 12.9, 10.5 Hz, 1H), 4.25 (ddd, *J* = 34.7, 13.7, 3.3 Hz, 1H), 4.03 (ddt, *J* = 86.5, 14.3, 7.4 Hz, 1H), 3.86 – 3.63 (m, 1H), 2.82 (q, *J* = 11.8 Hz, 1H), 2.69 (s, 2H), 2.62 (s, 1H), 2.38 (d, *J* = 5.0 Hz, 3H), 2.08 – 1.90 (m, 4H), 1.37 – 1.26 (m, 1H).

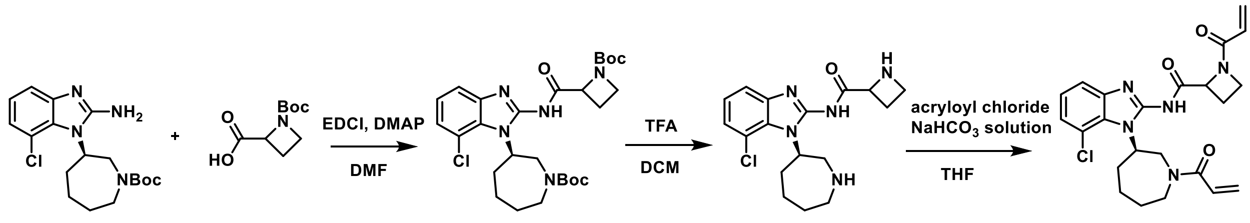

Synthesis of 1-acryloyl-*N*-(1-((*R*)-1-acryloylazepan-3-yl)-7-chloro-1*H*-benzo[*d*]imidazol-2-yl)azetidine-2-carboxamide (ZNL-0102)

tert-butyl (*R*)-3-(2-amino-7-chloro-1*H*-benzo[*d*]imidazol-1-yl)azepane-1-carboxylate **23** is prepared followed the procedure as previously described *(31)*. solution of *tert*-butyl (*R*)-3-(2-amino-7-chloro-1*H*-benzo[*d*]imidazol-1-yl)azepane-1-carboxylate **23** (100 mg, 1 eq, 0.27 mmol), 1-(*tert*-butoxycarbonyl)azetidine-2-carboxylic acid **24** (55 mg, 1 eq, 0.27 mmol), EDCI (83 mg, 2 eq, 0.54 mmol) and DMAP (16 mg, 0.5 eq, 0.13 mmol) in 3 mL of DMF was stirred at room

temperature for 2 hours. The reaction was poured into water (20 mL), extracted with EtOAc (10 mL x 3), washed with brine, dried with Na_2SO_4. The solvent was removed under reduced pressure then the residue was purified by column chromatography eluted (30% EtOAc in hexane) to afford *tert*-butyl (3*R*)-3-(2-(1-(*tert*-butoxycarbonyl)azetidine-2-carboxamido)-7-chloro-1*H*-benzo[*d*]imidazol-1-yl)azepane-1-carboxylate **25** (132 mg, 90 %). LC/MS (ESI) m/z calculated $[M+H]^+$ 548.26, found 548.35.

A mixture of *tert*-butyl (3*R*)-3-(2-(1-(*tert*-butoxycarbonyl)azetidine-2-carboxamido)-7-chloro-1*H*-benzo[*d*]imidazol-1-yl)azepane-1-carboxylate **25** (132 mg, 0.21 mmol) in TFA (1 mL) and DCM (3 mL) was stirred at room temperature for 1 hour. The mixture was concentrated to give crude *N*-(1-((*R*)-azepan-3-yl)-7-chloro-1*H*-benzo[*d*]imidazol-2-yl)azetidine-2-carboxamide **26** (80 mg) without further purification. LC/MS (ESI) m/z calculated $[M+H]^+$ 348.15, found 348.35.

To a solution of crude *N*-(1-((*R*)-azepan-3-yl)-7-chloro-1*H*-benzo[*d*]imidazol-2-yl)azetidine-2-carboxamide **26** (80 mg, 0.23 mmol) in THF (10 mL)/ saturated aqueous $NaHCO_3$ (2 mL) at 0^oC was added dropwise acryloyl chloride (1 M in DCM, 636 uL). The reaction was warmed to room temperature and stirred for 15 min. The reaction was purified by prep-HPLC (mobile phase: [H_2O (0.035% TFA)/MeOH (0.035% TFA)]; gradient:80% - 10% over 60 min) directly to afford 1-acryloyl-*N*-(1-((*R*)-1-acryloylazepan-3-yl)-7-chloro-1*H*-benzo[*d*]imidazol-2-yl)azetidine-2-carboxamide **ZNL-0102** (31 mg, yield 30%) as white solid. LC/MS (ESI) m/z calculated $[M+H]^+$ 456.94, found 456.30.

^{1}H NMR (500 MHz, DMSO-d_6) δ 12.88 (s, 1H), 7.53 (d, *J* = 7.7 Hz, 1H), 7.29 – 7.18 (m, 2H), 6.79 (dt, *J* = 16.7, 10.5 Hz, 1H), 6.46 – 6.01 (m, 3H), 5.76–5.49 (m, 2H), 5.30 (dtd, *J* = 39.0, 10.8, 5.5 Hz, 1H), 4.97 (dd, *J* = 9.8, 5.0 Hz, 1H), 4.57 – 3.71 (m, 5H), 2.76 – 2.60 (m, 1H), 2.18 (tdd, *J* = 12.5, 5.9, 2.9 Hz, 1H), 1.94 (td, *J* = 13.8, 6.0 Hz, 4H), 1.84 – 1.68 (m, 2H), 1.33 – 1.18 (m, 1H).

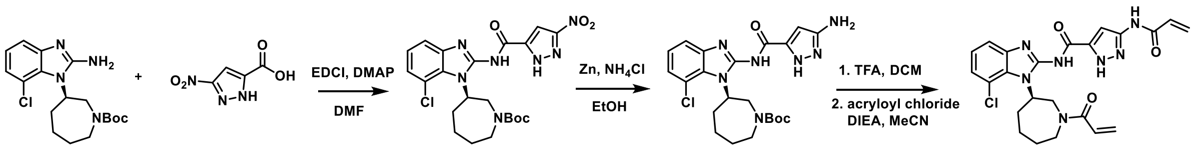

Synthesis of (*R*)-3-acrylamido-*N*-(1-(1-acryloylazepan-3-yl)-7-chloro-1*H*-benzo[*d*]imidazol-2-yl)-1*H*-pyrazole-5-carboxamide (ZNL-0166)

A solution of *tert*-butyl (*R*)-3-(2-amino-7-chloro-1*H*-benzo[*d*]imidazol-1-yl)azepane-1-carboxylate **23** (440 mg, 1 eq, 1.2 mmol), 3-nitro-1*H*-pyrazole-5-carboxylic acid (210 mg, 1.1 eq, 1.3 mmol), EDCI (370 mg, 2 eq, 2.4 mmol) and DMAP (74 mg, 0.5 eq, 0.6 mmol) in 12 mL of DMF was stirred at room temperature for 16 hours. The reaction was poured into water (40 mL), extracted with EtOAc (40 mL x 3), washed with brine, dried with Na_2SO_4. The solvent was removed under reduced pressure then the residue was purified by column chromatography eluted (30% EtOAc in hexane) to afford *tert*-butyl (*R*)-3-(7-chloro-2-(3-nitro-1*H*-pyrazole-5-carboxamido)-1*H*-benzo[*d*]imidazol-1-yl)azepane-1-carboxylate **28** (340 mg, 56 %). LC/MS (ESI) m/z calculated $[M+H]^+$ 504.94, found 504.19.

To a solution of *tert*-butyl (*R*)-3-(7-chloro-2-(3-nitro-1*H*-pyrazole-5-carboxamido)-1*H*-benzo[*d*]imidazol-1-yl)azepane-1-carboxylate **28** (340 mg, 1 eq, 0.675 mmol) in EtOH (2 mL)/ saturated aqueous $NaHCO_3$ (in water 2 mL) at 70°C was added Zn powder (440 mg, 10 eq, 6.75 mmol) with stirring for 30 min. The reaction was cooled to room temperature and filtered. The filtrate was concentrated under reduce pressure. The residue was dissolved in EtOAc (50 mL)/brine (30 mL), the organic layer was concentrated. The crude product was purified through prep-HPLC to afford *tert*-butyl (*R*)-3-(2-(3-amino-1*H*-pyrazole-5-carboxamido)-7-chloro-1*H*-benzo[*d*]imidazol-1-yl)azepane-1-carboxylate **29** (110 mg, 34%) as brown solid. LC/MS (ESI) m/z calculated $[M+H]^+$ 474.96, found 474.15.

A mixture of *tert*-butyl (*R*)-3-(2-(3-amino-1*H*-pyrazole-5-carboxamido)-7-chloro-1*H*-benzo[*d*]imidazol-1-yl)azepane-1-carboxylate **29** (110 mg, 0.23 mmol) in TFA (1mL) and DCM (3 mL) was stirred at room temperature for 1 hour. The mixture was concentrated to give crude (*R*)-3-amino-*N*-(1-(azepan-3-yl)-7-chloro-1*H*-benzo[*d*]imidazol-2-yl)-1*H*-pyrazole-5-carboxamide (78 mg). To a solution of (*R*)-3-amino-*N*-(1-(azepan-3-yl)-7-chloro-1*H*-benzo[*d*]imidazol-2-yl)-1*H*-pyrazole-5-carboxamide (78 mg, 1 eq, 0.21 mmol) and DIEA (110 µL, 3 eq, 0.63 mmol) in acetonitrile (3 mL) at room temperature was added dropwise acryloyl chloride (0.5 M in DCM, 110 µL, 2.2 eq) during 10 min. The reaction was stirred for 5 min at room temperature. The reaction was purified through prep-HPLC (mobile phase: [H_2O (0.035% TFA)/MeOH (0.035% TFA)]; gradient:70% - 30% over 60 min) to afford (*R*)-3-acrylamido-*N*-(1-(1-acryloylazepan-3-yl)-7-chloro-1*H*-benzo[*d*]imidazol-2-yl)-1*H*-pyrazole-5-carboxamide **ZNL-0166** (16 mg, 16%) as white solid. LC/MS (ESI) m/z calculated $[M+H]^+$ 482.94, found 482.25.

^{1}H NMR (400 MHz, DMSO-d_6) δ 12.98 (s, 1H), 10.75 (d, *J* = 10.9 Hz, 1H), 7.63 – 7.52 (m, 1H), 7.40 – 7.03 (m, 3H), 6.86 (dt, *J* = 16.7, 9.7 Hz, 1H), 6.49 (dd, *J* = 17.0, 10.2 Hz, 1H), 6.35 – 6.04 (m, 2H), 5.86 – 5.62 (m, 2H), 5.40 (d, *J* = 55.0 Hz, 1H), 4.88 – 4.53 (m, 1H), 4.29 – 4.12 (m, 1H), 3.90 – 3.58 (m, 1H), 3.23 (dd, *J* = 12.7, 6.9 Hz, 1H), 2.74 – 2.58 (m, 1H), 2.21 – 1.69 (m, 4H), 1.37 – 1.23 (m, 2H).

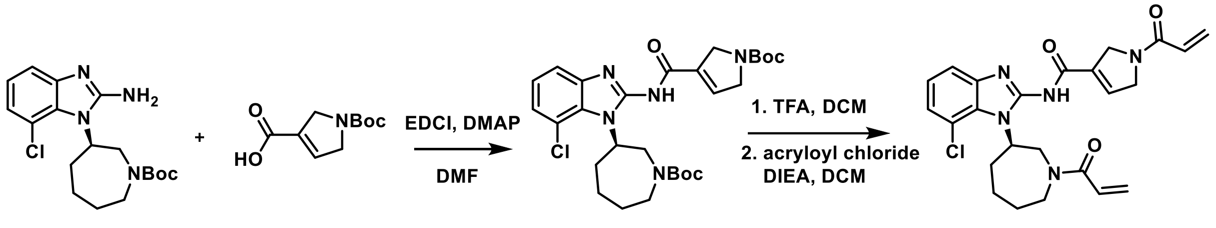

Synthesis of (*R*)-1-acryloyl-*N*-(1-(1-acryloylazepan-3-yl)-7-chloro-1*H*-benzo[*d*]imidazol-2-yl)-2,5-dihydro-1*H*-pyrrole-3-carboxamide (ZNL-0178)

A solution of *tert*-butyl (*R*)-3-(2-amino-7-chloro-1*H*-benzo[*d*]imidazol-1-yl)azepane-1-carboxylate **23** (100 mg, 1 eq, 0.27 mmol), 1-(*tert*-butoxycarbonyl)-2,5-dihydro-1*H*-pyrrole-3-carboxylic acid **30** (58 mg, 1 eq, 0.27 mmol), EDCI (83 mg, 2 eq, 0.54 mmol) and DMAP (16 mg, 0.5 eq, 0.13 mmol) in 3 mL of DMF was stirred at room temperature for 2 hours. The reaction was poured into water, extracted with EtOAc , washed with brine, dried with Na_2SO_4. The solvent was removed under reduced pressure then the residue was purified by column chromatography eluted (30% EtOAc in hexane) to afford *tert*-butyl (*R*)-3-(2-(1-(*tert*-butoxycarbonyl)-2,5-dihydro-1*H*-pyrrole-3-carboxamido)-7-chloro-1*H*-benzo[*d*]imidazol-1-yl)azepane-1-carboxylate **31** (90 mg, 60 %). LC/MS (ESI) m/z calculated $[M+H]^+$ 560.26, found 560.16.

A mixture of *tert*-butyl (*R*)-3-(2-(1-(*tert*-butoxycarbonyl)-2,5-dihydro-1*H*-pyrrole-3-carboxamido)-7-chloro-1*H*-benzo[*d*]imidazol-1-yl)azepane-1-carboxylate **31** (90 mg, 0.16 mmol) in TFA (1 mL) and DCM (3 mL) was stirred at room temperature for 1 hour. The mixture was concentrated. The residue was disolved into DCM (3 mL), DIEA (120 µL, 0.63 mmol) and acryloyl chloride (0.5 M in DCM, 100 µL) was added at room temperature, the reaction was further stirred for 15 min. The reaction was diluted with water extracted with EtOAc. The combined organic phase was dried over Na_2SO_4, concentrated under reduce pressure. The crude product was purified through prep-HPLC (mobile phase: [H_2O (0.035% TFA)/MeOH (0.035% TFA)];

gradient:80% - 10% over 60 min) to afford (*R*)-1-acryloyl-*N*-(1-(1-acryloylazepan-3-yl)-7-chloro-1*H*-benzo[*d*]imidazol-2-yl)-2,5-dihydro-1*H*-pyrrole-3-carboxamide **ZNL-0178** (26 mg, yield 36% over 2 steps) as white solid. LC/MS (ESI) m/z calculated $[M+H]^+$ 468.95, found 468.30.

^{1}H NMR (500 MHz, DMSO) δ 12.89 (s, 1H), 7.54 (dd, *J* = 7.9, 3.0 Hz, 1H), 7.25 (dd, *J* = 25.8, 8.0 Hz, 2H), 6.97 – 6.76 (m, 1H), 6.73 – 6.56 (m, 2H), 6.24 – 6.13 (m, 2H), 5.79 – 5.64 (m, 2H), 5.50 – 5.26 (m, 1H), 4.81 – 4.55 (m, 2H), 4.49 – 4.42 (m, 1H), 4.39 – 4.28 (m, 1H), 4.17 (dd, *J* = 13.0, 3.7 Hz, 1H), 4.07 (tt, *J* = 12.2, 5.7 Hz, 1H), 3.97 – 3.74 (m, 0.5H), 3.63– 3.47 (m, 0.5H), 3.21 (ddt, *J* = 16.6, 13.7, 5.6 Hz, 0.5H), 2.57 (dd, *J* = 13.6, 10.1 Hz, 1H), 2.17 – 1.70 (m, 4H), 1.39 – 1.22 (m, 1.5H).

Reference

(1) Engelman, J. A.; Mukohara, T.; Zejnullahu, K.; Lifshits, E.; Borras, A. M.; Gale, C. M.; Naumov, G. N.; Yeap, B. Y.; Jarrell, E.; Sun, J.; et al. Allelic dilution obscures detection of a biologically significant resistance mutation in EGFR-amplified lung cancer. *J Clin Invest.* **2006**, *116*, 2695-2706.

(2) Jia, Y.; Yun, C. H.; Park, E.; Ercan, D.; Manuia, M.; Juarez, J.; Xu, C.; Rhee, K.; Chen, T.; Zhang, H.; et al. Overcoming EGFR(T790M) and EGFR(C797S) resistance with mutant-selective allosteric inhibitors. *Nature.* **2016**, *534*, 129-132.

(3) Zhang, J.; Adrian, F. J.; Jahnke, W.; Cowan-Jacob, S. W.; Li, A. G.; Iacob, R. E.; Sim, T.; Powers, J.; Dierks, C.; Sun, F.; et al. Targeting Bcr-Abl by combining allosteric with ATP-binding-site inhibitors. *Nature.* **2010**, *463*, 501-506.

(4) Patricelli, M. P.; Szardenings, A. K.; Liyanage, M.; Nomanbhoy, T. K.; Wu, M.; Weissig, H.; Aban, A.; Chun, D.; Tanner, S.; Kozarich, J. W. Functional interrogation of the kinome using nucleotide acyl phosphates. *Biochemistry.* **2007**, *46*, 350-358.

(5) Winter, G.; Waterman, D. G.; Parkhurst, J. M.; Brewster, A. S.; Gildea, R. J.; Gerstel, M.; Fuentes-Montero, L.; Vollmar, M.; Michels-Clark, T.; Young, I. D.; et al. DIALS: implementation and evaluation of a new integration package. *Acta Crystallogr D Struct Biol.* **2018**, *74*, 85-97.

(6) Gildea, R. J.; Beilsten-Edmands, J.; Axford, D.; Horrell, S.; Aller, P.; Sandy, J.; Sanchez-Weatherby, J.; Owen, C. D.; Lukacik, P.; Strain-Damerell, C.; et al. xia2.multiplex: a multi-crystal data-analysis pipeline. *Acta Crystallogr D Struct Biol.* **2022**, *78*, 752-769.

(7) Adams, P. D.; Afonine, P. V.; Bunkoczi, G.; Chen, V. B.; Davis, I. W.; Echols, N.; Headd, J. J.; Hung, L. W.; Kapral, G. J.; Grosse-Kunstleve, R. W.; et al. PHENIX: a comprehensive Python-based system for macromolecular structure solution. *Acta Crystallogr D Biol Crystallogr.* **2010**, *66*, 213-221.

(8) Beyett, T. S.; To, C.; Heppner, D. E.; Rana, J. K.; Schmoker, A. M.; Jang, J.; De Clercq, D. J. H.; Gomez, G.; Scott, D. A.; Gray, N. S.; et al. Molecular basis for cooperative binding and synergy of ATP-site and allosteric EGFR inhibitors. *Nat Commun.* **2022**, *13*, 2530.

(9) Emsley, P.; Lohkamp, B.; Scott, W. G.; Cowtan, K. Features and development of Coot. *Acta Crystallogr D Biol Crystallogr.* **2010**, *66*, 486-501.

(10) Maurer, T. S.; Tabrizi-Fard, M. A.; Fung, H. L. Impact of mechanism-based enzyme inactivation on inhibitor potency: implications for rational drug discovery. *J Pharm Sci.* **2000**, *89*, 1404-1414.
(11) Adasme, M. F.; Linnemann, K. L.; Bolz, S. N.; Kaiser, F.; Salentin, S.; Haupt, V. J.; Schroeder, M. PLIP 2021: expanding the scope of the protein-ligand interaction profiler to DNA and RNA. *Nucleic Acids Res.* **2021**, *49*, W530-W534.
(12) Ficarro, S. B.; Zhang, Y.; Lu, Y.; Moghimi, A. R.; Askenazi, M.; Hyatt, E.; Smith, E. D.; Boyer, L.; Schlaeger, T. M.; Luckey, C. J.; et al. Improved electrospray ionization efficiency compensates for diminished chromatographic resolution and enables proteomics analysis of tyrosine signaling in embryonic stem cells. *Anal Chem.* **2009**, *81*, 3440-3447.
(13) Ficarro, S. B.; Alexander, W. M.; Marto, J. A. mzStudio: A Dynamic Digital Canvas for User-Driven Interrogation of Mass Spectrometry Data. *Proteomes.* **2017**, *5*, 20-27.
(14) Alexander, W. M.; Ficarro, S. B.; Adelmant, G.; Marto, J. A. multiplierz v2.0: A Python-based ecosystem for shared access and analysis of native mass spectrometry data. *Proteomics.* **2017**, *17*, 1700091-1700099.

^{1}HNMR spectra

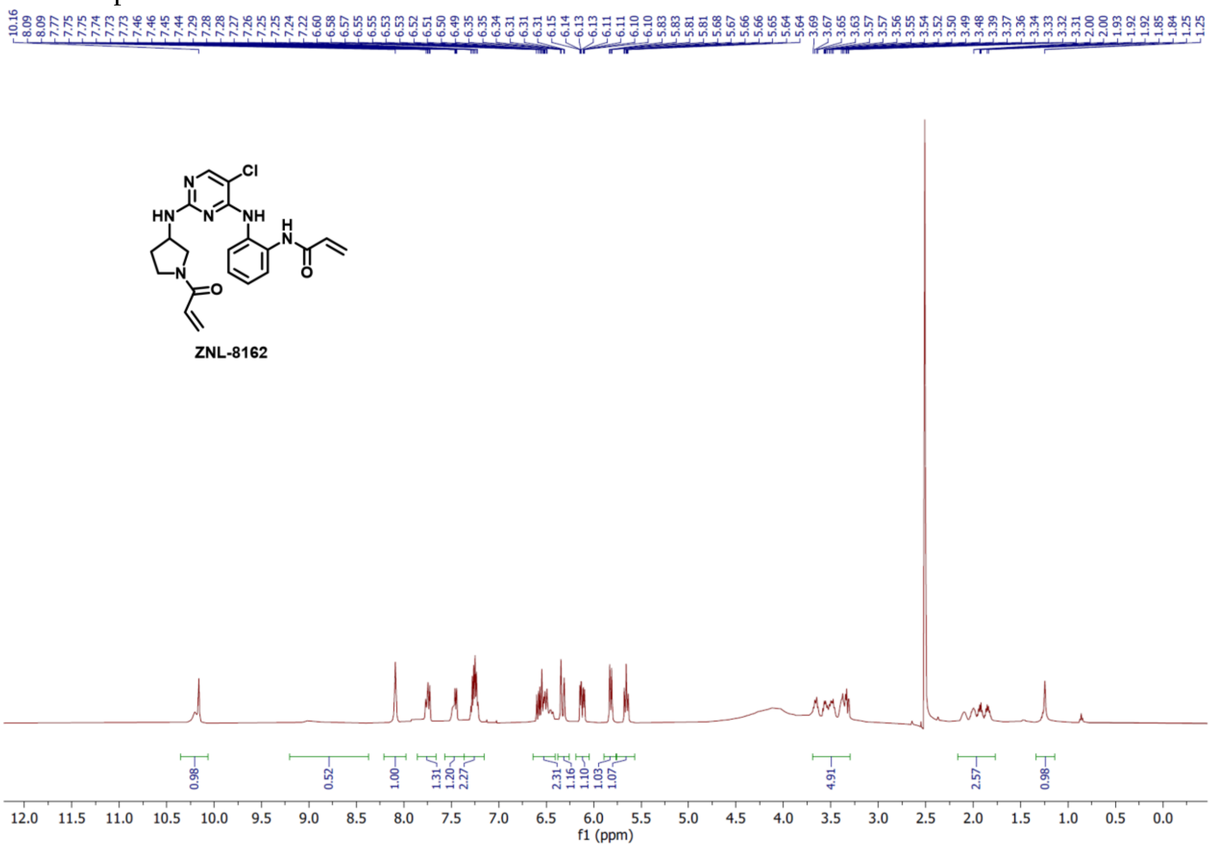
ZNL-8162

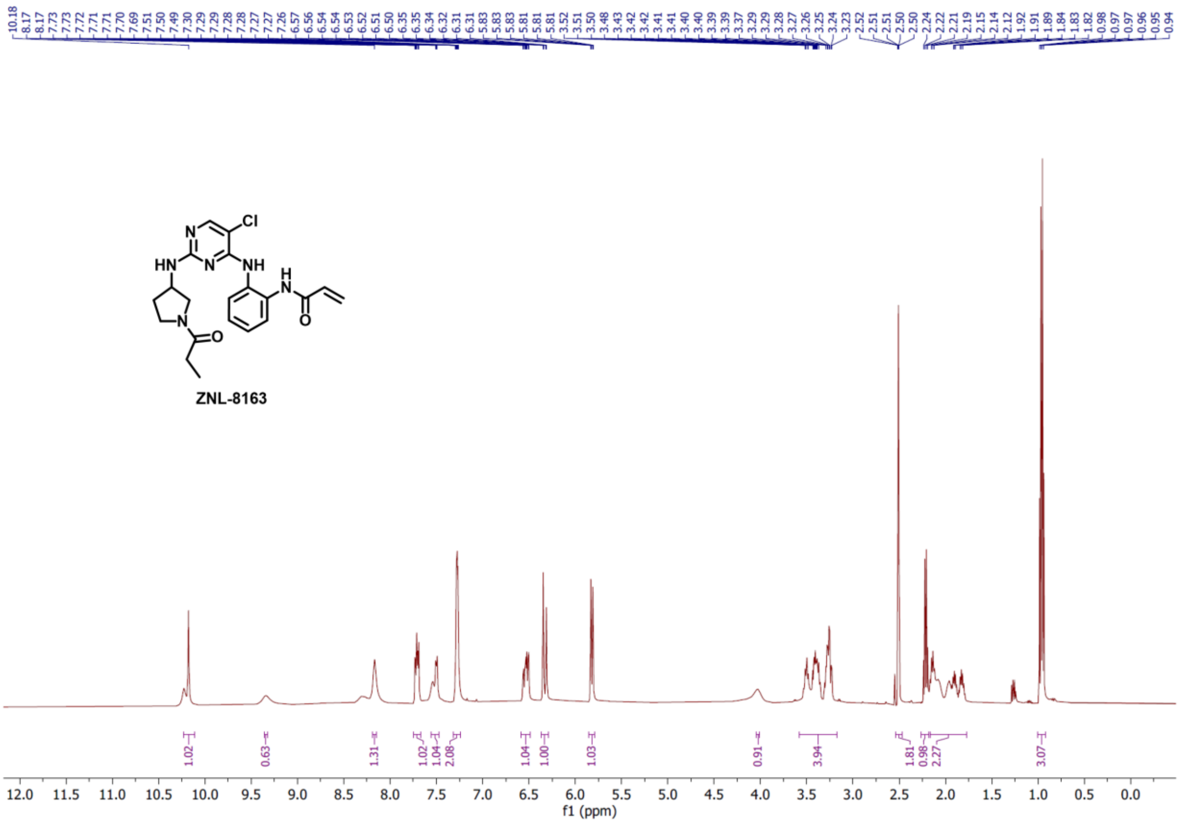
ZNL-8163

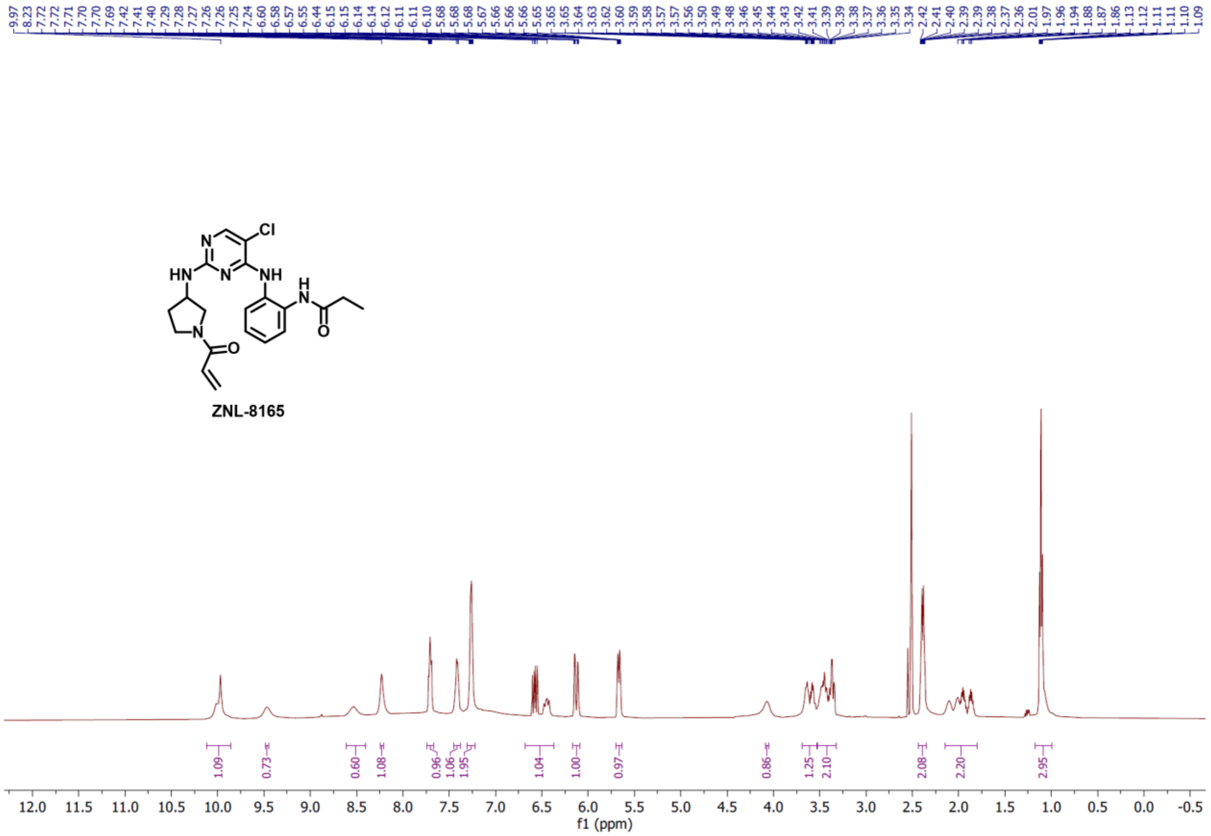
ZNL-8165
f1 (ppm)

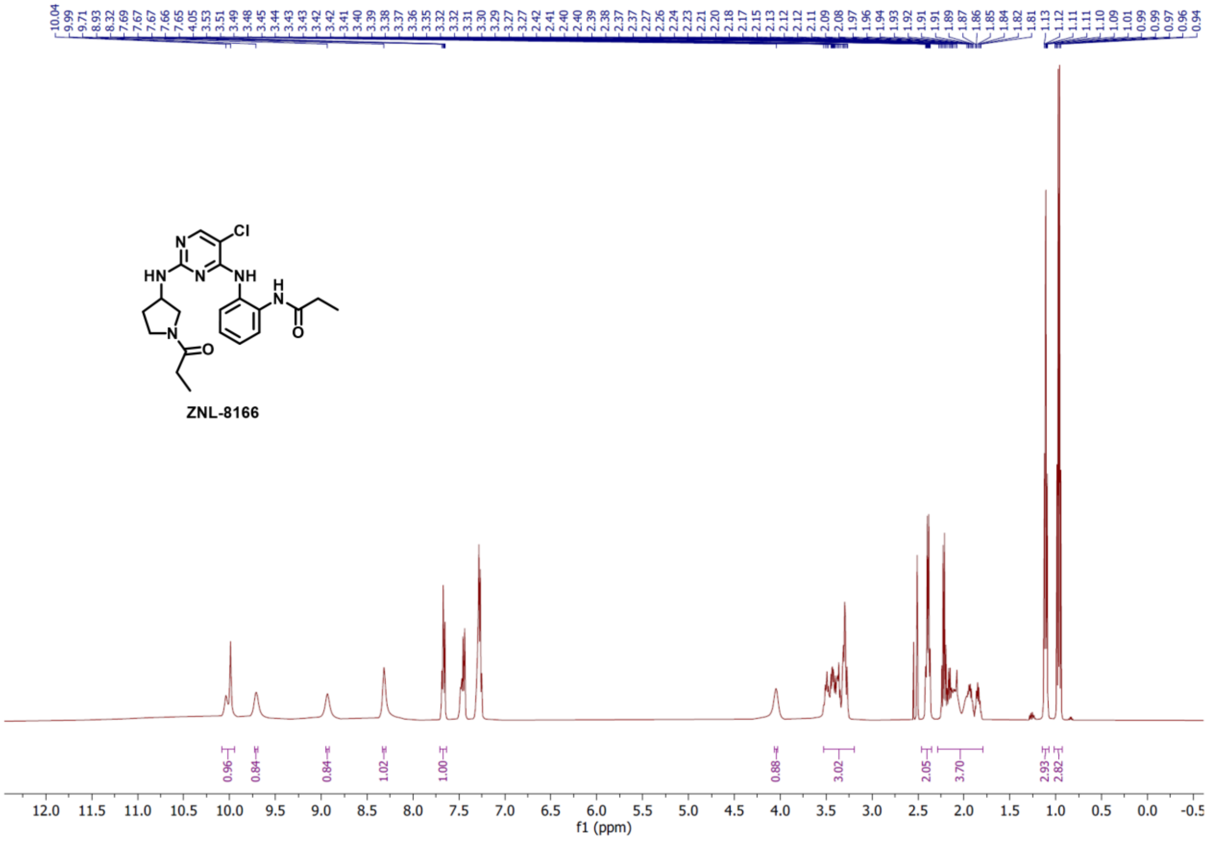
ZNL-8166
f1 (ppm)

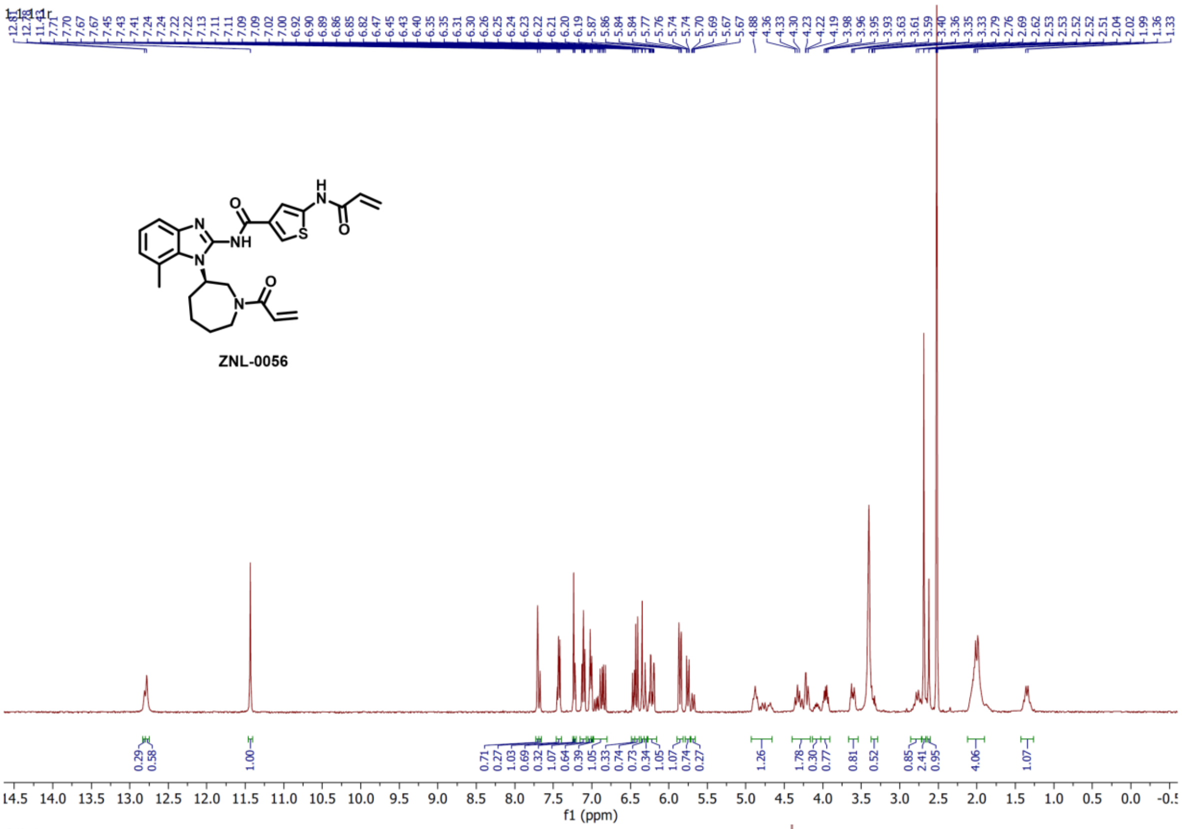
ZNL-0056
f1 (ppm)

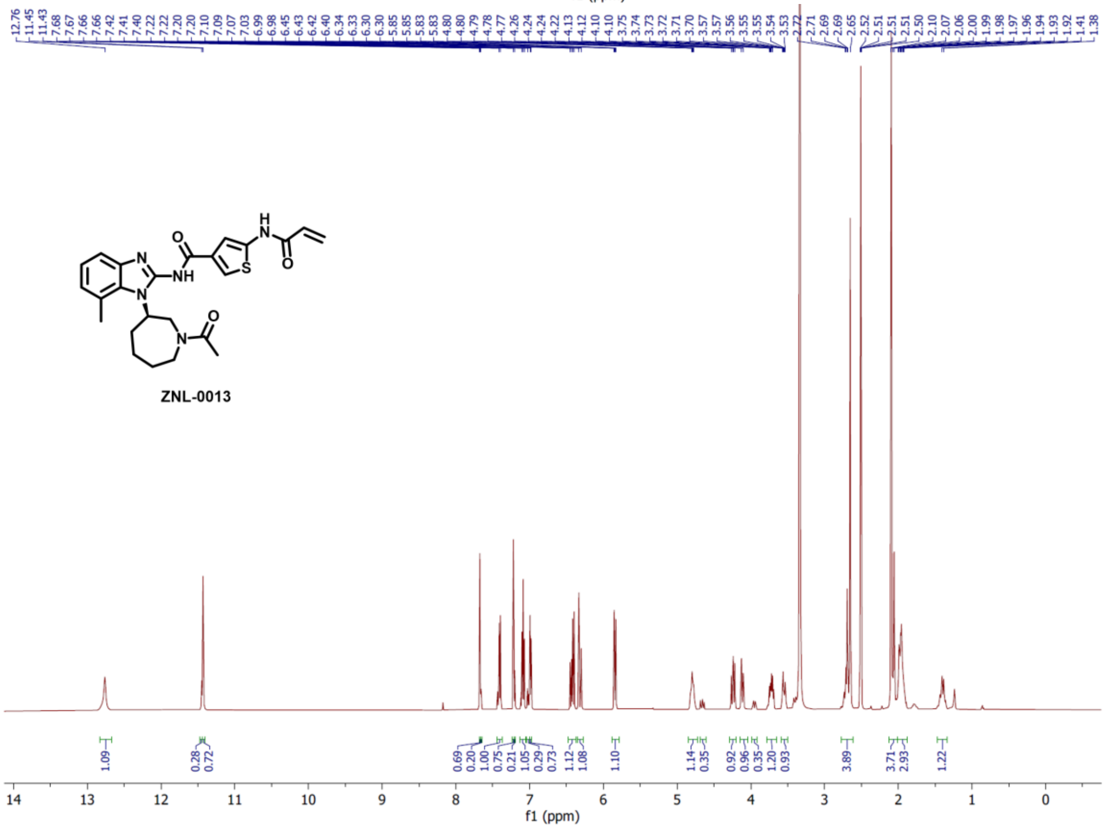
ZNL-0013
f1 (ppm)

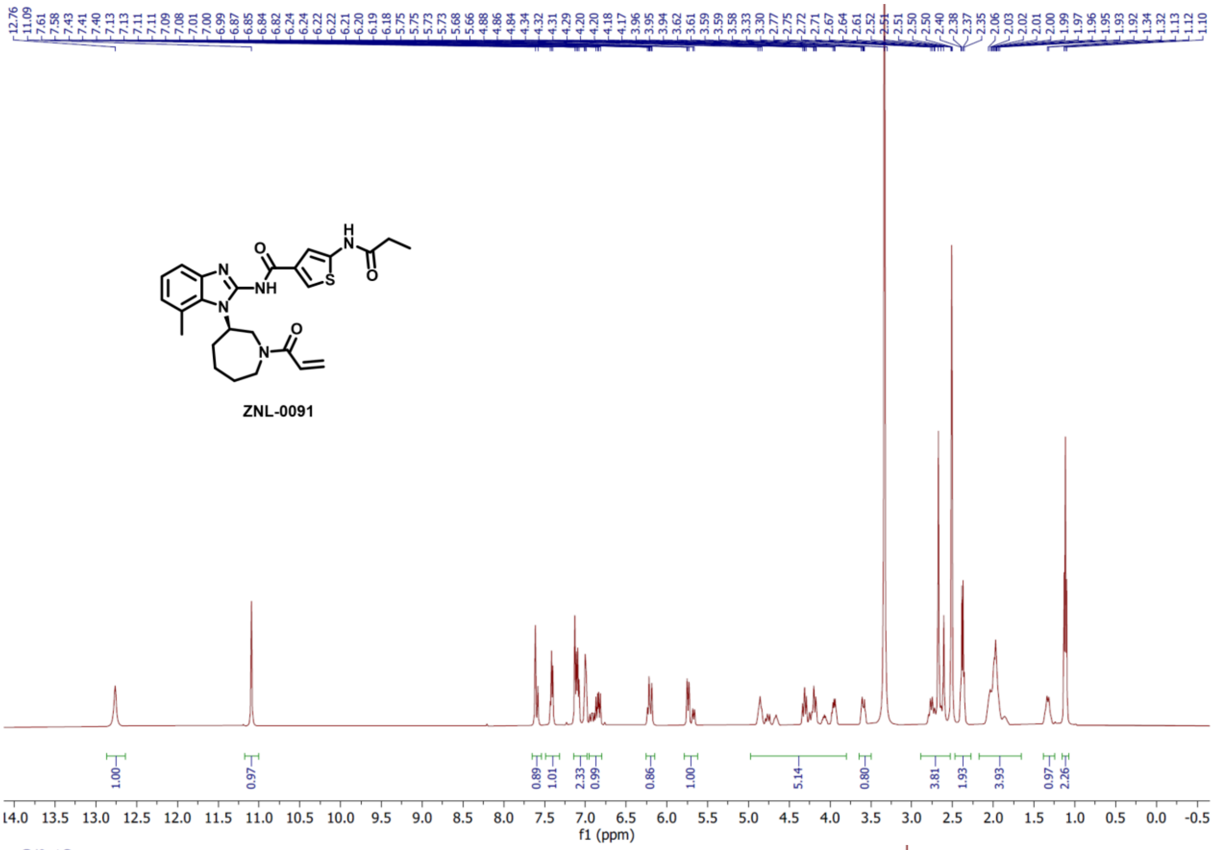

ZNL-0091

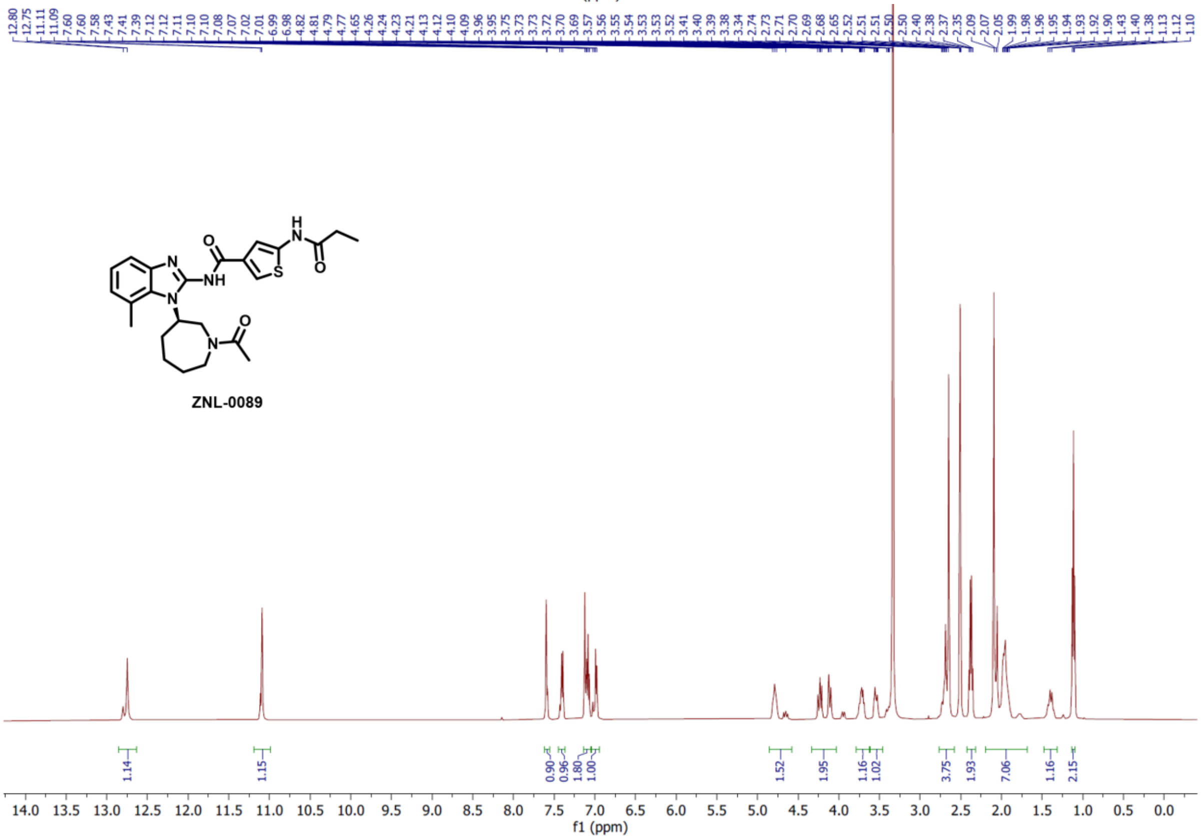

ZNL-0089

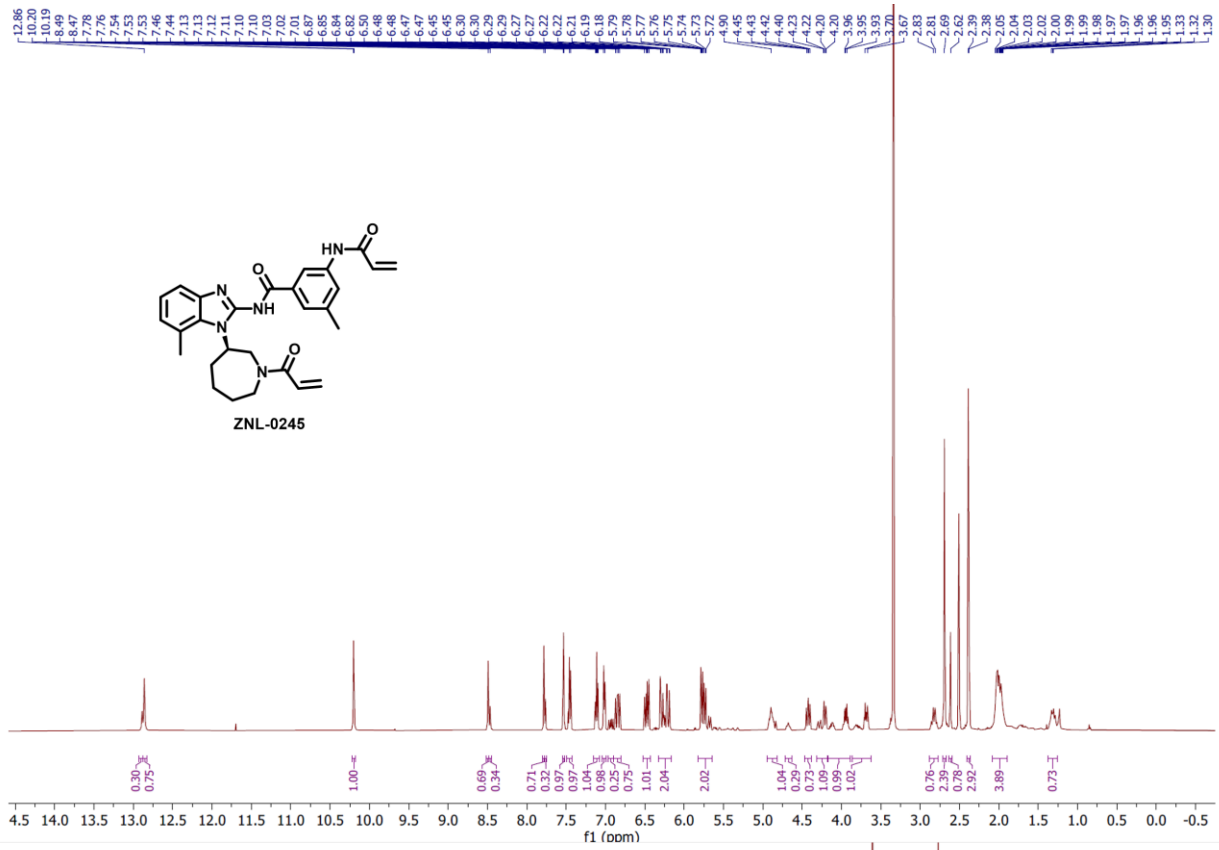
ZNL-0245
f1 (ppm)

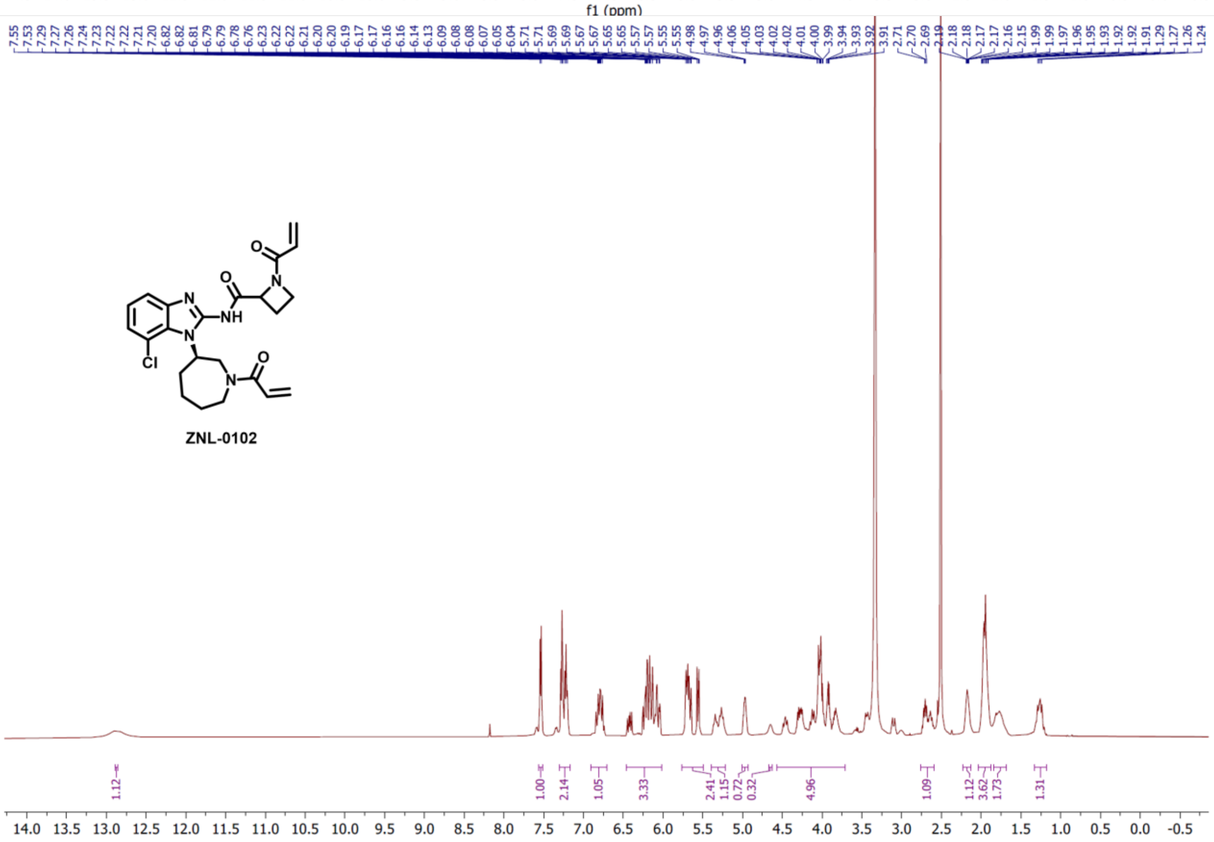
ZNL-0102

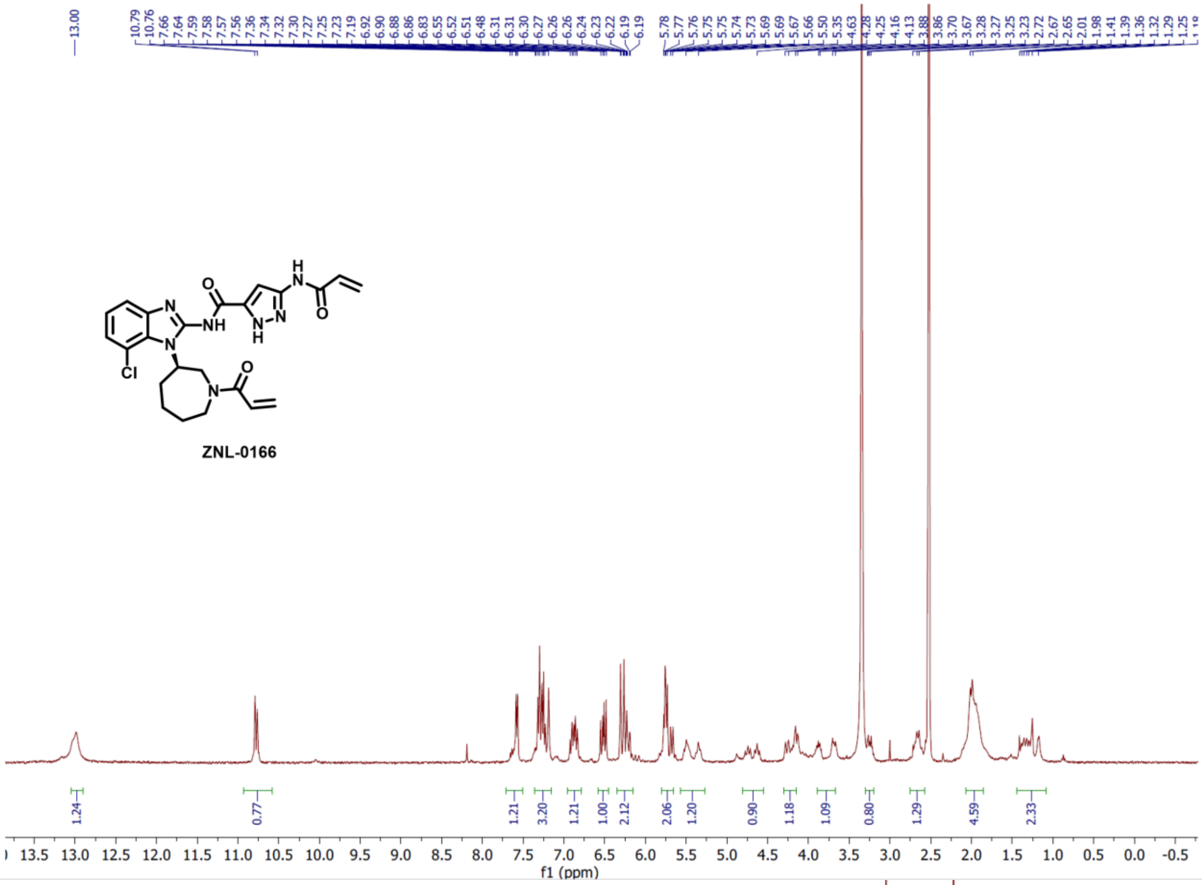
13.00
10.79 10.76 7.66 7.64 7.59 7.58 7.57 7.56 7.36 7.34 7.32 7.30 7.27 7.25 7.23 7.19 6.92 6.90 6.88 6.86 6.83 6.55 6.52 6.51 6.48 6.31 6.31 6.30 6.27 6.26 6.26 6.24 6.23 6.22 6.19 6.19 6.19
5.78 5.77 5.76 5.75 5.75 5.74 5.73 5.69 5.69 5.67 5.66 5.50 5.35 4.63 4.28 4.25 4.16 4.13 3.88 3.86 3.70 3.67 3.28 3.27 3.25 3.23 2.72 2.67 2.65 2.01 1.98 1.41 1.39 1.36 1.32 1.29 1.25
ZNL-0166
1.24 0.77 1.21 3.20 1.21 1.00 2.12 2.06 1.20 0.90 1.18 1.09 0.80 1.29 4.59 2.33
13.5 13.0 12.5 12.0 11.5 11.0 10.5 10.0 9.5 9.0 8.5 8.0 7.5 7.0 6.5 6.0 5.5 5.0 4.5 4.0 3.5 3.0 2.5 2.0 1.5 1.0 0.5 0.0 -0.5
f1 (ppm)

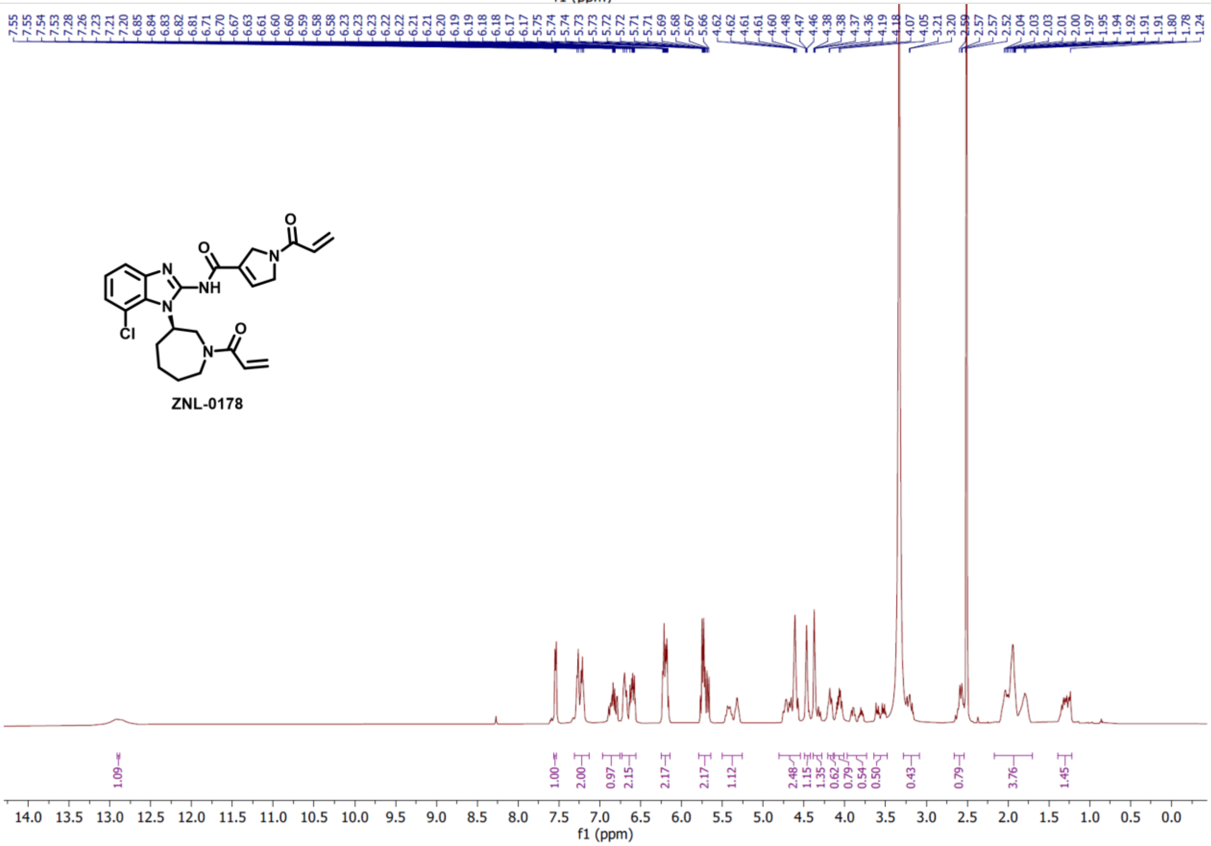
7.55 7.55 7.54 7.53 7.28 7.26 7.23 7.21 7.20 6.85 6.84 6.83 6.82 6.81 6.71 6.70 6.67 6.63 6.61 6.60 6.60 6.59 6.58 6.58 6.23 6.23 6.23 6.22 6.22 6.21 6.21 6.20 6.19 6.19 6.18 6.18 6.17 6.17 5.75 5.74 5.74 5.73 5.73 5.72 5.72 5.71 5.71 5.69 5.68 5.67 5.66 4.62 4.61 4.61 4.60 4.48 4.47 4.46 4.38 4.38 4.37 4.36 4.19 4.18 4.07 4.05 3.21 3.20 2.59 2.57 2.57 2.52 2.04 2.03 2.01 2.00 1.97 1.95 1.94 1.92 1.91 1.91 1.80 1.78 1.24
ZNL-0178
1.09 1.00 2.00 0.97 2.15 2.17 2.17 1.12 2.48 1.15 1.35 0.62 0.79 0.54 0.50 0.43 0.79 3.76 1.45
14.0 13.5 13.0 12.5 12.0 11.5 11.0 10.5 10.0 9.5 9.0 8.5 8.0 7.5 7.0 6.5 6.0 5.5 5.0 4.5 4.0 3.5 3.0 2.5 2.0 1.5 1.0 0.5 0.0
f1 (ppm)

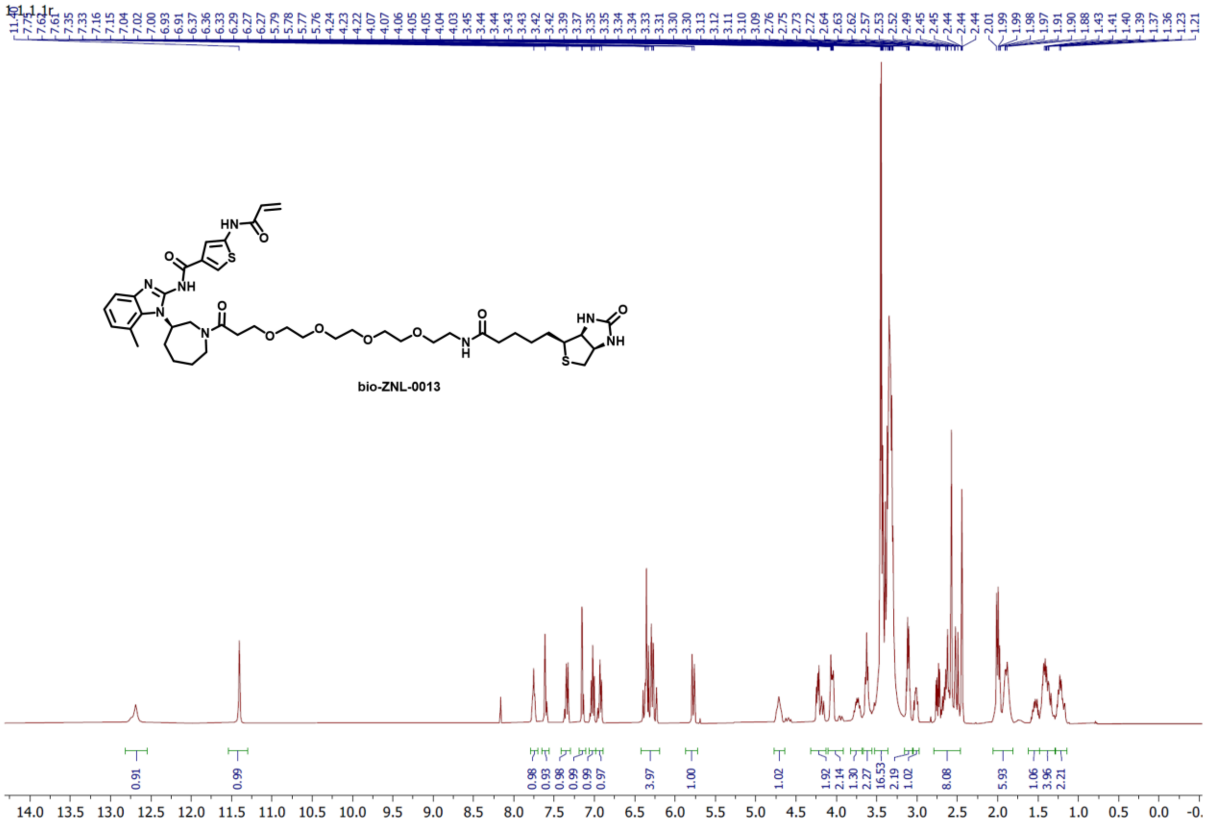
bio-ZNL-0013
