# Supplementary material for: Molecular Bidents with Two Electrophilic Warheads as a New Pharmacological Modality
Source: ACS Cent Sci. 2024 Feb 26;10(6):1156–66. doi: 10.1021/acscentsci.3c01245 (PMC11212140; doi:10.1021/acscentsci.3c01245)
Supplement: Supplementary file 5 — oc3c01245_si_005.pdf [file oc3c01245_si_005.pdf]

ccaagtcattctgagaatagtgtatgcggcgaccgagttgctcttgcccggcGTCAATACGGGATAATACCGCGCCACATA
GCAGAACTTTAAAAGTGCTCATCATTGGAAAACGTTCTTCGGGGCGAAAACTCTCAAGGATCTTACCGCT
GTTGAGATCCAGTTCGATGTAACCCACTCGTGCACCCAACTGATCTTCAGCATCTTTTACTTTCACCAGCG
TTTCTGGGTGAGCAAAAACAGGAAGGCAAAATGCCGCAAAAAAGGGAATAAGGGCGACACGGAAATG
TTGAATACTCATACTCTTCCTTTTTCAATATTATTGAAGCATTTATCAGGGTTATTGTCTCATGAGCGGATA
CATATTTGAATGTATTTAGAAAAATAAACAAATAGGGGTTCCGCGCACATTTCCCCGAAAAGTGCCACCT
GACGTCTAAGAAACCATTATTATCATGACATTAACCTATAAAAATAGGCGTATCACGAGGCCCTTTCGTC
TCGCGCGTTTCGGTGATGACGGTGAAAACCTCTGACACATGCAGCTCCCGGAGACGGTCACAGCTTGTC
TGTAAGCGGATGCCGGGAGCAGACAAGCCCGTCAGGGCGCGTCAGCGGGTGTTGGCGGGTGTCGGGG
CTGGCTTAACTATGCGGCATCAGAGCAGATTGTACTGAGAGTGCACCATATGGACATATTGTCGTTAGA
ACGCGGCTACAATTAATACATAACCTTATGTATCATACACATACGATTTAGGTGACACTATAGAACTCGA
GCAGCTGAAGCTTGCATGCCAGCTTGCATGCCTGCAGGTCGGAGTACTGTCCTCCGAGCGGAGTACTGT
CCTCCGAGCGGAGTACTGTCCTCCGAGCGGAGTACTGTCCTCCGAGCGGAGTACTGTCCTCCGAGCGGA
GACTCTAGAGGGTATATAATGGATCTAGTCTTATGCAATACTCTTGTAGTCTTGCAACATGGTAACGATG
AGTTAGCAACATGCCTTACAAGGAGAGAAAAAGCACCGTGCATGCCGATTGGTGGAAGTAAGGTGGTA
CGATCGTGCCTTATTAGGAAGGCAACAGACGGGTCTGACATGGATTGGACGAACCACTGAATTGCCGCA
TTGCAGAGATATTGTATTTAAGTGCCTAGCTCGATACATAAACGGGTCTCTCTGGTTAGACCAGATCTGA
GCCTGGGAGCTCTCTGGCTAACTAGGGAACCCACTGCTTAAGCCTCAATAAAGCTTGCCTTGAGTGCTTC
AAGTAGTGTGTGCCCGTCTGTTGTGTGACTCTGGTAACTAGAGATCCCTCAGACCCTTTTAGTCAGTGTG
GAAAATCTCTAGCAGTGGCGCCCGAACAGGGACTTGAAAGCGAAAGGGAAACCAGAGGAGCTCTCTCG
ACGCAGGACTCGGCTTGCTGAAGCGCGCACGGCAAGAGGCGAGGGGCGGCGACTGGTGAGTACGCCA
AAAATTTTGACTAGCGGAGGCTAGAAGGAGAGAGATGGGTGCGAGAGCGTCAGTATTAAGCGGGGGA
GAATTAGATCGCGATGGGAAAAAATTCGGTTAAGGCCAGGGGGAAAGAAAAAATATAAATTAAAACAT
ATAGTATGGGCAAGCAGGGAGCTAGAACGATTCGCAGTTAATCCTGGCCTGTTAGAAACATCAGAAGG
CTGTAGACAAATACTGGGACAGCTACAACCATCCCTTCAGACAGGATCAGAAGAACTTAGATCATTATAT
AATACAGTAGCAACCCTCTATTGTGTGCATCAAAGGATAGAGATAAAAGACACCAAGGAAGCTTTAGAC
AAGATAGAGGAAGAGCAAAACAAAAGTAAGACCACCGCACAGCAAGGGCCGCTGATCTTCAGACCTGG
AGGAGGAGATATGAGGGACAATTGGAGAAGTGAATTATATAAATATAAAGTAGTAAAAATTGAACCAT
TAGGAGTAGCACCCACCAAGGCAAAGAGAAGAGTGGTGCAGAGAGAAAAAAGAGCAGTGGGAATAG
GAGCTTTGTTCCTTGGGTTCTTGGGAGCAGCAGGAAGCACTATGGGCGCAGCGTCAATGACGCTGACG
GTACAGGCCAGACAATTATTGTCTGGTATAGTGCAGCAGCAGAACAATTTGCTGAGGGCTATTGAGGCG
CAACAGCATCTGTTGCAACTCACAGTCTGGGGCATCAAGCAGCTCCAGGCAAGAATCCTGGCTGTGGAA
AGATACCTAAAGGATCAACAGCTCCTGGGGATTTGGGGTTGCTCTGGAAAACTCATTTGCACCACTGCT
GTGCCTTGGAATGCTAGTTGGAGTAATAAATCTCTGGAACAGATTTGGAATCACACGACCTGGATGGAG
TGGGACAGAGAAATTAACAATTACACAAGCTTAATACACTCCTTAATTGAAGAATCGCAAAACCAGCAA
GAAAAGAATGAACAAGAATTATTGGAATTAGATAAATGGGCAAGTTTGTGGAATTGGTTTAACATAACA
AATTGGCTGTGGTATATAAAATTATTCATAATGATAGTAGGAGGCTTGGTAGGTTTAAGAATAGTTTTTG
CTGTACTTTCTATAGTGAATAGAGTTAGGCAGGGATATTCACCATTATCGTTTCAGACCCACCTCCCAACC
CCGAGGGGACCCGACAGGCCCGAAGGAATAGAAGAAGAAGGTGGAGAGAGAGACAGAGACAGATCC
ATTCGATTAGTGAACGGATCTCGACGGTATCGATAAGCTTGGGGATATCTCTCGACCTCGAGACAAATG
GCAGTATTCATCCACAATTTTAAAAGAAAAGGGGGGATTGGGGGGTACAGTGCAGGGGAAAGAATAGT
AGACATAATAGCAACAGACATACAAACTAAAGAATTACAAAAACAAAGTCGACTCCGGCCATTAGCCAT
ATTATTCATTGGTTATATAGCATAAATCAATATTGGCTATTGGCCATTGCATACGTTGTATCCATATCATA
ATATGTACATTTATATTGGCTCATGTCCAACATTACCGCCATGTTGACATTGATTATTGACTAGTTATTAAT

AGTAATCAATTACGGGGTCATTAGTTCATAGCCCATATATGGAGTTCCGCGTTACATAACTTACGGTAAA
TGGCCCGCCTGGCTGACCGCCCAACGACCCCCGCCCATTGACGTCAATAATGACGTATGTTCCCATAGTA
ACGCCAATAGGGACTTTCCATTGACGTCAATGGGTGGAGTATTTACGGTAAACTGCCCACTTGGCAGTA
CATCAAGTGTATCATATGCCAAGTACGCCCCCTATTGACGTCAATGACGGTAAATGGCCCGCCTGGCATT
ATGCCCAGTACATGACCTTATGGGACTTTCCTACTTGGCAGTACATCTACGTATTAGTCATCGCTATTACC
ATGGTGATGCGGTTTTGGCAGTACATCAATGGGCGTGGATAGCGGTTTGACTCACGGGGATTTCCAAGT
CTCCACCCCATTGACGTCAATGGGAGTTTGTTTTGGCACCAAAATCAACGGGACTTTCCAAAATGTCGTA
ACAACTCCGCCCCATTGACGCAAATGGGCGGTAGGCGTGTACGGTGGGAGGTCTATATAAGCAGAGCT
CGTTTAGTGAACCGTCAGATCGCCTGGAGACGCCATCCACGCTGTTTTGACCTCCATAGAAGACACCGG
GACCGATCCAGCCTCCGCGGCCCCAAGCTTCGATAACTTCGTATAGCATACATTATACGAAGTTATCAGT
CGACACCATGCGACCCTCCGGGACGGCCGGGGCAGCGCTCCTGGCGCTGCTGGCTGCGCTCTGCCCGG
CGAGTCGGGCTCTGGAGGAAAAGAAAGTTTGCCAAGGCACGAGTAACAAGCTCACGCAGTTGGGCACT
TTTGAAGATCATTTTCTCAGCCTCCAGAGGATGTTCAATAACTGTGAGGTGGTCCTTGGGAATTTGGAAA
TTACCTATGTGCAGAGGAATTATGATCTTTCCTTCTTAAAGACCATCCAGGAGGTGGCTGGTTATGTCCT
CATTGCCCTCAACACAGTGGAGCGAATTCCTTTGGAAAACCTGCAGATCATCAGAGGAAATATGTACTA
CGAAAATTCCTATGCCTTAGCAGTCTTATCTAACTATGATGCAAATAAAACCGGACTGAAGGAGCTGCCC
ATGAGAAATTTACAGGAAATCCTGCATGGCGCCGTGCGGTTCAGCAACAACCCTGCCCTGTGCAACGTG
GAGAGCATCCAGTGGCGGGACATAGTCAGCAGTGACTTTCTCAGCAACATGTCGATGGACTTCCAGAAC
CACCTGGGCAGCTGCCAAAAGTGTGATCCAAGCTGTCCCAATGGGAGCTGCTGGGGTGCAGGAGAGGA
GAACTGCCAGAAACTGACCAAAATCATCTGTGCCCAGCAGTGCTCCGGGCGCTGCCGTGGCAAGTCCCC
CAGTGACTGCTGCCACAACCAGTGTGCTGCAGGCTGCACAGGCCCCCGGGAGAGCGACTGCCTGGTCT
GCCGCAAATTCCGAGACGAAGCCACGTGCAAGGACACCTGCCCCCCACTCATGCTCTACAACCCCACCAC
GTACCAGATGGATGTGAACCCCGAGGGCAAATACAGCTTTGGTGCCACCTGCGTGAAGAAGTGTCCCC
GTAATTATGTGGTGACAGATCACGGCTCGTGCGTCCGAGCCTGTGGGGCCGACAGCTATGAGATGGAG
GAAGACGGCGTCCGCAAGTGTAAGAAGTGCGAAGGGCCTTGCCGCAAAGTGTGTAACGGAATAGGTAT
TGGTGAATTTAAAGACTCACTCTCCATAAATGCTACGAATATTAAACACTTCAAAAACTGCACCTCCATCA
GTGGCGATCTCCACATCCTGCCGGTGGCATTTAGGGGTGACTCCTTCACACATACTCCTCCTCTGGATCC
ACAGGAACTGGATATTCTGAAAACCGTAAAGGAAATCACAGGGTTTTTGCTGATTCAGGCTTGGCCTGA
AAACAGGACGGACCTCCATGCCTTTGAGAACCTAGAAATCATACGCGGCAGGACCAAGCAACATGGTCA
GTTTTCTCTTGCAGTCGTCAGCCTGAACATAACATCCTTGGGATTACGCTCCCTCAAGGAGATAAGTGAT
GGAGATGTGATAATTTCAGGAAACAAAAATTTGTGCTATGCAAATACAATAAACTGGAAAAAACTGTTT
GGGACCTCCGGTCAGAAAACCAAAATTATAAGCAACAGAGGTGAAAACAGCTGCAAGGCCACAGGCCA
GGTCTGCCATGCCTTGTGCTCCCCCGAGGGCTGCTGGGGCCCGGAGCCCAGGGACTGCGTCTCTTGCCG
GAATGTCAGCCGAGGCAGGGAATGCGTGGACAAGTGCAACCTTCTGGAGGGTGAGCCAAGGGAGTTT
GTGGAGAACTCTGAGTGCATACAGTGCCACCCAGAGTGCCTGCCTCAGGCCATGAACATCACCTGCACA
GGACGGGGACCAGACAACTGTATCCAGTGTGCCCACTACATTGACGGCCCCCACTGCGTCAAGACCTGC
CCGGCAGGAGTCATGGGAGAAAACAACACCCTGGTCTGGAAGTACGCAGACGCCGGCCATGTGTGCCA
CCTGTGCCATCCAAACTGCACCTACGGATGCACTGGGCCAGGTCTTGAAGGCTGTCCAACGAATGGGCC
TAAGATCCCGTCCATCGCCACTGGGATGGTGGGGGCCCTCCTCTTGCTGCTGGTGGTGGCCCTGGGGAT
CGGCCTCTTCATGCGAAGGCGCCACATCGTTCGGAAGCGCACGCTGCGGAGGCTGCTGCAGGAGAGGG
AGCTTGTGGAGCCTCTTACACCCAGTGGAGAAGCTCCCAACCAAGCTCTCTTGAGGATCTTGAAGGAAA
CTGAATTCAAAAAGATCAAAGTGCTGGGCTCCGGTGCGTTCGGCACGGTGTATAAGGGACTCTGGATCC
CAGAAGGTGAGAAAGTTAAAATTCCCGTCGCTATCAAGGAATTAAGAGAAGCAACATCTCCGAAAGCCA
ACAAGGAAATCCTCGATGAAGCCTACGTGATGGCCAGCGTGGACAACCCCCACGTGAGCCGCCTGCTG

GGCATCTGCCTCACCTCCACCGTGCAGCTCATCACGCAGCTCATGCCCTTCGGCTGCCTCCTGGACTATGT
CCGGGAACACAAAGACAATATTGGCTCCCAGTACCTGCTCAACTGGTGTGTGCAGATCGCAAAGGGCAT
GAACTACTTGGAGGACCGTCGCTTGGTGCACCGCGACCTGGCAGCCAGGAACGTACTGGTGAAAACAC
CGCAGCATGTCAAGATCACAGATTTTGGGCGGGCCAAACTGCTGGGTGCGGAAGAGAAAGAATACCAT
GCAGAAGGAGGCAAAGTGCCTATCAAGTGGATGGCATTGGAATCAATTTTACACAGAATCTATACCCAC
CAGAGTGATGTCTGGAGCTACGGGGTGACCGTTTGGGAGTTGATGACCTTTGGATCCAAGCCATATGAC
GGAATCCCTGCCAGCGAGATCTCCTCCATCCTGGAGAAAGGAGAACGCCTCCCTCAGCCACCCATATGT
ACCATCGATGTCTACATGATCATGGTCAAGTGCTGGATGATAGACGCAGATAGTCGCCCAAAGTTCCGT
GAGTTGATCATCGAATTCTCCAAAATGGCCCGAGACCCCCAGCGCTACCTTGTCATTCAGGGGGATGAA
AGAATGCATTTGCCAAGTCCTACAGACTCCAACTTCTACCGTGCCCTGATGGATGAAGAAGACATGGAC
GACGTGGTGGATGCCGACGAGTACCTCATCCCACAGCAGGGCTTCTTCAGCAGCCCCTCCACGTCACGG
ACTCCCCTCCTGAGCTCTCTGAGTGCAACCAGCAACAATTCCACCGTGGCTTGCATTGATAGAAATGGGC
TGCAAAGCTGTCCCATCAAGGAAGACAGCTTCTTGCAGCGATACAGCTCAGACCCCACAGGCGCCTTGA
CTGAGGACAGCATAGACGACACCTTCCTCCCAGTGCCTGAATACATAAACCAGTCCGTTCCCAAAAGGCC
CGCTGGCTCTGTGCAGAATCCTGTCTATCACAATCAGCCTCTGAACCCCGCGCCCAGCAGAGACCCACAC
TACCAGGACCCCCACAGCACTGCAGTGGGCAACCCCGAGTATCTCAACACTGTCCAGCCCACCTGTGTCA
ACAGCACATTCGACAGCCCTGCCCACTGGGCCCAGAAAGGCAGCCACCAAATTAGCCTGGACAACCCTG
ACTACCAGCAGGACTTCTTTCCCAAGGAAGCCAAGCCAAATGGCATCTTTAAGGGCTCCACAGCTGAAA
ATGCAGAATACCTAAGGGTCGCGCCACAAAGCAGTGAATTTATTGGAGCATAGGGAAGCTTTCTAGACC
ATTCGTTTGGCGCGCGGGCCCAGGTAAGTGGTCATAATCATAATCATAATCATAATCATAATCACAACTA
GCCTAGGAGATCCTGGTCATGACTAGTGCTTGGATTCTCACCAATAAAAAACGCCCGGCGGCAACCGAG
CGTTCTGAACAAATCCAGATGGAGTTCTGAGGTCATTACTGGATCTATCAACAGGAGTCCAAGCGAGCT
CGATATCAAATTACGCCCCGCCCTGCCACTCATCGCAGTACTGTTGTAATTCATTAAGCATTCTGCCGACA
TGGAAGCCATCACAAACGGCATGATGAACCTGAATCGCCAGCGGCATCAGCACCTTGTCGCCTTGCGTA
TAATATTTGCCCATGGTGAAAACGGGGGCGAAGAAGTTGTCCATATTGGCCACGTTTAAATCAAAACTG
GTGAAACTCACCCAGGGATTGGCTGAGACGAAAAACATATTCTCAATAAACCCTTTAGGGAAATAGGCC
AGGTTTTCACCGTAACACGCCACATCTTGCGAATATATGTGTAGAAACTGCCGGAAATCGTCGTGGTATT
CACTCCAGAGCGATGAAAACGTTTCAGTTTGCTCATGGAAAACGGTGTAACAAGGGTGAACACTATCCC
ATATCACCAGCTCACCGTCTTTCATTGCCATACGAAATTCCGGATGAGCATTCATCAGGCGGGCAAGAAT
GTGAATAAAGGCCGGATAAAACTTGTGCTTATTTTTCTTTACGGTCTTTAAAAAGGCCGTAATATCCAGC
TGAACGGTCTGGTTATAGGTACATTGAGCAACTGACTGAAATGCCTCAAAATGTTCTTTACGATGCCATT
GGGATATATCAACGGTGGTATATCCAGTGATTTTTTTCTCCATTTTAGCTTCCTTAGCTCCTGAAAGATCC
ATAACTTCGTATAGCATACATTATACGAAGTTATAGATCCAATATTATTGAAGCATTTATCAGGGTTATTG
TCTCATGAGCGGATACATATTTGAATGTATTTAGAAAAATAAACAAATAGGGGTTCCGCGCACATTTCCC
CGAAAAGTGCCACCTGACGGGCGCGCCCTACCGGGTAGGGGAGGCGCTTTTCCCAAGGCAGTCTGGAG
CATGCGCTTTAGCAGCCCCGCTGGGCACTTGGCGCTACACAAGTGGCCTCTGGCCTCGCACACATTCCAC
ATCCACCGGTAGGCGCCAACCGGCTCCGTTCTTTGGTGGCCCCTTCGCGCCACCTTCTACTCCTCCCCTAG
TCAGGAAGTTCCCCCCCGCCCCGCAGCTCGCGTCGTGCAGGACGTGACAAATGGAAGTAGCACGTCTCA
CTAGTCTCGTGCAGATGGACAGCACCGCTGAGCAATGGAAGCGGGTAGGCCTTTGGGGCAGCGGCCAA
TAGCAGCTTTGCTCCTTCGCTTTCTGGGCTCAGAGGCTGGGAAGGGGTGGGTCCGGGGGCGGGCTCAG
GGGCGGGCTCAGGGGCGGGGCGGGCGCCCGAAGGTCCTCCGGAGGCCCGGCATTCTGCACGCTTCAA
AAGCGCACGTCTGCCGCGCTGTTCTCCTCTTCCTCATCTCCGGGCCTTTCGACTCTAGACACGTGTTGACA
ATTAATCATCGGCATAGTATATCGGCATAGTATAATACGACAAGGTGAGGAACTAAACCATGGCCGAGT
ACAAGCCCACGGTGCGCCTCGCCACCCGCGACGACGTCCCCCGGGCCGTACGCACCCTCGCCGCCGCGT

TCGCCGACTACCCCGCCACGCGCCACACCGTCGACCCGGACCGCCACATCGAGCGGGTCACCGAGCTGC
AAGAACTCTTCCTCACGCGCGTCGGGCTCGACATCGGCAAGGTGTGGGTCGCGGACGACGGCGCCGCG
GTGGCGGTCTGGACCACGCCGGAGAGCGTCGAAGCGGGGGCGGTGTTCGCCGAGATCGGCCCGCGCA
TGGCCGAGTTGAGCGGTTCCCGGCTGGCCGCGCAGCAACAGATGGAAGGCCTCCTGGCGCCGCACCGG
CCCAAGGAGCCCGCGTGGTTCCTGGCCACCGTCGGCGTCTCGCCCGACCACCAGGGCAAGGGTCTGGG
CAGCGCCGTCGTGCTCCCCGGAGTGGAGGCGGCCGAGCGCGCCGGGGTGCCCGCCTTCCTGGAGACCT
CCGCGCCCCGCAACCTCCCCTTCTACGAGCGGCTCGGCTTCACCGTCACCGCCGACGTCGAGGTGCCCG
AAGGACCGCGCACCTGGTGCATGACCCGCAAGCCCGGTGCCTGAGGATCCGTATTCTTAACTATGTTGC
TCCTTTTACGCTATGTGGATACGCTGCTTTAATGCCTTTGTATCATGCTATTGCTTCCCGTATGGCTTTCAT
TTTCTCCTCCTTGTATAAATCCTGGTTGCTGTCTCTTTATGAGGAGTTGTGGCCCGTTGTCAGGCAACGTG
GCGTGGTGTGCACTGTGTTTGCTGACGCAACCCCCACTGGTTGGGGCATTGCCACCACCTGTCAGCTCCT
TTCCGGGACTTTCGCTTTCCCCCTCCCTATTGCCACGGCGGAACTCATCGCCGCCTGCCTTGCCCGCTGCT
GGACAGGGGCTCGGCTGTTGGGCACTGACAATTCCGTGGTGTTGTCGGGGAAGCTGACGTCCTTTCCAT
GGCTGCTCGCCTGTGTTGCCACCTGGATTCTGCGCGGGACGTCCTTCTGCTACGTCCCTTCGGCCCTCAA
TCCAGCGGACCTTCCTTCCCGCGGCCTGCTGCCGGCTCTGCGGCCTCTTCCGCGTCTTCGCCTTCGCCCTC
AGACGAGTCGGATCTCCCTTTGGGCCGCCTCCCCGCCTGTTTCGCCTCGGCGTCCGGCCCGGGCACAATT
CGAGCTCGGTACCTTTAAGACCAATGACTTACAAGGCAGCTGTAGATCTTAGCCACTTTTTAAAAGAAAA
GGGGGGACTGGAAGGGCTAATTCACTCCCAACGAAGACAAGATCTGCTTTTTGCTTGTACTTTTATTTGT
GAAATTTGTGATGCTATTGCTTTATTTGTAACCATGGGTCTCTCTGGTTAGACCAGATCTGAGCCTGGGA
GCTCTCTGGCTAACTAGGGAACCCACTGCTTAAGCCTCAATAAAGCTTGCCTTGAGTGCTTCAAGTAGTG
TGTGCCCGTCTGTTGTGTGACTCTGGTAACTAGAGATCCCTCAGACCCTTTTAGTCAGTGTGGAAAATCT
CTAGCAGTAGTAGGCTAGCGGTGTGGAAATCCCCAGGCTCCCCAGCAGGCAGAAGTATGCAAAGCATG
CATCTCAATTAGTCAGCAACCATAGTCCCGCCCCTAACTCCGCCCATCCCGCCCCTAACTCCGCCCAGTTC
CGCCCATTCTCCGCCCCATGGCTGACTAATTTTTTTTATTTATGCAGAGGCCGAGGCCGCCTCGGCCTCTG
AGCTATTCCAGAAGTAGTGAGGAGGCTTTTTTGGAGGCCTAGGCTTTTGCAAAGATCGATCAAGAGACA
GGATGAGGATCGTTTCGCATGGAATTCATCGATGATATCAGATCTGCCGGTCTCCCTATAGTGAGTCGTA
TTAATTTCGATAAGCCAGGTTAACCTGCATTAATGAATCGGCCAACGCGCGGGGAGAGGCGGTTTGCGT
ATTGGGCGCTCTTCCGCTTCCTCGCTCACTGACTCGCTGCGCTCGGTCGTTCGGCTGCGGCGAGCGGTAT
CAGCTCACTCAAAGGCGGTAATACGGTTATCCACAGAATCAGGGGATAACGCAGGAAAGAACATGTGA
GCAAAAGGCCAGCAAAAGGCCAGGAACCGTAAAAAGGCCGCGTTGCTGGCGTTTTTCCATAGGCTCCG
CCCCCCTGACGAGCATCACAAAAATCGACGCTCAAGTCAGAGGTGGCGAAACCCGACAGGACTATAAA
GATACCAGGCGTTTCCCCCTGGAAGCTCCCTCGTGCGCTCTCCTGTTCCGACCCTGCCGCTTACCGGATA
CCTGTCCGCCTTTCTCCCTTCGGGAAGCGTGGCGCTTTCTCATAGCTCACGCTGTAGGTATCTCAGTTCGG
TGTAGGTCGTTCGCTCCAAGCTGGGCTGTGTGCACGAACCCCCCGTTCAGCCCGACCGCTGCGCCTTATC
CGGTAACTATCGTCTTGAGTCCAACCCGGTAAGACACGACTTATCGCCACTGGCAGCAGCCACTGGTAA
CAGGATTAGCAGAGCGAGGTATGTAGGCGGTGCTACAGAGTTCTTGAAGTGGTGGCCTAACTACGGCT
ACACTAGAAGAACAGTATTTGGTATCTGCGCTCTGCTGAAGCCAGTTACCTTCGGAAAAAGAGTTGGTA
GCTCTTGATCCGGCAAACAAACCACCGCTGGTAGCGGTGGTTTTTTTGTTTGCAAGCAGCAGATTACGCG
CAGAAAAAAAGGATCTCAAGAAGATCCTTTGATCTTTTCTACGGGGTCTGACGCTCAGTGGAACGAAAA
CTCACGTTAAGGGATTTTGGTCATGAGATTATCAAAAAGGATCTTCACCTAGATCCTTTTAAATTAAAAAT
GAAGTTTTAAATCAATCTAAAGTATATATGAGTAAACTTGGTCTGACAGTTACCAATGCTTAATCAGTGA
GGCACCTATCTCAGCGATCTGTCTATTTCGTTCATCCATAGTTGCCTGACTCCCCGTCGTGTAGATAACTA
CGATACGGGAGGGCTTACCATCTGGCCCCAGTGCTGCAATGATACCGCGAGACCCACGCTCACCGGCTC
CAGATTTATCAGCAATAAACCAGCCAGCCGGAAGGGCCGAGCGCAGAAGTGGTCCTGCAACTTTATCCG

CCTCCATCCAGTCTATTAATTGTTGCCGGGAAGCTAGAGTAAGTAGTTCGCCAGTTAATAGTTTGCGCAA
CGTTGTTGCCATTGCTACAGGCATCGTGGTGTCACGCTCGTCGTTTGGTATGGCTTCATTCAGCTCCGGT
TCCCAACGATCAAGGCGAGTTACATGATCCCCCATGTTGTGCAAAAAAGCGGTTAGCTCCTTCGGTCCTC
CGATCGTTGTCAGAAGTAAGTTGGCCGCAGTGTTATCACTCATGGTTATGGCAGCACTGCATAATTCTCT
TACTGTCATGCCATCCGTAAGATGCTTTTCTGTGACTGGTGAGTACTCAA
